# Afann: bias adjustment for alignment-free sequence comparison based on sequencing data using neural network regression

**DOI:** 10.1186/s13059-019-1872-3

**Published:** 2019-12-04

**Authors:** Kujin Tang, Jie Ren, Fengzhu Sun

**Affiliations:** Quantitative and Computational Biology Program, Department of Biological Sciences, University of Southern California, Los Angeles, CA USA

**Keywords:** Alignment-free, Neural network regression, *k*mer, $d_{2}^{*}, \protect d_{2}^{s}$, NGS, Bias adjustment

## Abstract

Alignment-free methods, more time and memory efficient than alignment-based methods, have been widely used for comparing genome sequences or raw sequencing samples without assembly. However, in this study, we show that alignment-free dissimilarity calculated based on sequencing samples can be overestimated compared with the dissimilarity calculated based on their genomes, and this bias can significantly decrease the performance of the alignment-free analysis. Here, we introduce a new alignment-free tool, Alignment-Free methods Adjusted by Neural Network (Afann) that successfully adjusts this bias and achieves excellent performance on various independent datasets. Afann is freely available at https://github.com/GeniusTang/Afann.

## Background

With the advent of next-generation sequencing (NGS) technologies, enormous amounts of sequence data are emerging rapidly. Although alignment-based approaches for sequence comparison are generally accurate and powerful, their applications are being challenged by the size of sequence data that increases at an exponential rate. More importantly, the application of alignment-based methods in NGS analysis could also be limited when the sequencing depth is low so that assembled contigs might not share long homologous regions that could be aligned. Throughout the paper, the sequencing depth (fold coverage) is measured by the total number of sequenced bases divided by the genome length. Therefore, alignment-free methods, alternatives over alignment-based methods, have recently received increasing attention because they are generally more memory and time efficient [1–9]. Moreover, alignment-free methods, especially *k*mer-based approaches that use the frequencies of *k*mers (*k*-words or *k*-grams) for sequence comparison can be naturally adapted to shotgun NGS sequencing data without assembly [4, 5, 8–12]. Recently, Zielezinski et al. [9] published a comprehensive comparison over 74 alignment-free methods for 5 research applications including *cis*-regulatory module detection, protein sequence classification, gene tree inference, genome-based phylogeny, and reconstruction of species trees under sequence rearrangements.

Based on the rationale that similar sequences share similar *k*mer frequency profile, also known as genomic signature [13], *k*mer-based alignment-free methods first count the number of occurrences of *k*mers along a sequence or in an NGS sample and characterize each sequence or an NGS sample as a feature vector of length 4^*K*^. Second, transformation can be applied to normalize the *k*mer count vector or to remove the random background of *k*mer counts using a Markov model [1, 2]. Alignment-free methods that remove the random background are also known as background-adjusted methods such as *CVTree*[1], $d_{2}^{s}$[2], and $d_{2}^{*}$[2]. In addition, dissimilarity measures such as Manhattan distance, Euclidean distance, Mash (Jaccard distance) [5], and Cosine distance are used to compare any pair of sequence-representing feature vectors.

Since *k*mer frequency can be counted directly from raw NGS samples, *k*mer-based alignment-free methods can be easily adapted to compare NGS samples without assembly. This adaptation relies on a strong assumption that the sequence-representing feature vectors of NGS samples can be used as alternatives of sequence-representing feature vectors of their genomes, and thus, the alignment-free dissimilarity calculated based on the NGS samples should be close to the dissimilarity calculated based on their genomes. While this assumption is reasonable when sequencing depth is high because of the law of large numbers, it can nevertheless be compromised by low sequencing depth, sequencing error, and sequencing bias. For example, for any alignment-free method, the dissimilarity between a genome and itself should be 0 because their feature vectors should be exactly the same whereas the dissimilarity between two NGS samples sampled from the same genome will be greater than 0 since their feature vectors will be different due to the stochastic distribution of reads along the genomes. Therefore, it is expected that the dissimilarity calculated based on the NGS samples will most likely be overestimated than the dissimilarity calculated based on their genomes, and the overestimation will increase as the sequencing depth decreases, which has also been revealed in several studies based on various alignment-free methods [4, 8, 12]. This bias, which refers to the overestimated dissimilarity based on NGS samples, is a common problem for all alignment-free methods since it results from the intrinsic stochastic distribution of short reads regardless of the choice of dissimilarity measures.

The alignment-free dissimilarity between two NGS samples A and B is determined by three factors which are alignment-free dissimilarity estimated based on their genomes, the bias caused by random sampling of NGS sample A, and the bias caused by random sampling of NGS sample B. Comparing NGS samples without bias adjustment may thus be misguided and be prone to drawing conclusions that are inconsistent with analysis based on their genomes. This can be explained by the fact that the high dissimilarity between two NGS samples does not necessarily imply the high dissimilarity between their genomes. It could also result from the large bias caused by low sequencing depth. Therefore, the relative order of pairwise dissimilarity between NGS samples and dissimilarity between genomes will be different if the sequencing depths of NGS samples are different. For example, suppose genome A is closer to genome B than to genome C based on their complete genomes. All three genomes are sequenced using NGS, and the sequencing depth of genome B is lower than that of genome C. Since the dissimilarity between two genomes using NGS data increases as the sequencing depth decreases, it is possible that the dissimilarity between A and B is higher than that between A and C based on NGS data, resulting in incorrect relationships among the genomes A, B, and C.

One feasible solution is to downsample all NGS samples to the same number of reads or the same total number of sequenced bases if the lengths of reads are different [12]. While biases are not adjusted, they can nevertheless be controlled at the same level after downsampling. As a result, the dissimilarity between NGS samples is affected by the same level of bias, and the relative order of pairwise dissimilarity between NGS samples should be determined only by their genome dissimilarity. However, this method causes a huge waste of reads since all samples will be downsampled to the same sequencing quantity as the smallest sample, and thus, a vast majority of informative reads in other samples will be discarded, which could have been included to improve the performance.

Another solution is to modify the formula of alignment-free dissimilarity by considering sequencing depth and sequencing error. To the best of our knowledge, AAF [4] and Skmer [8] are the only existing methods that account for sequencing depth and sequencing error and adjust the alignment-free dissimilarity accordingly. AAF first infers a phylogenetic tree of a group of genomes and then corrects all branch lengths (tip correction) based on the average fold coverage of all NGS samples. However, since samples of high sequencing depth tend to group together as aforementioned, tip correction after phylogeny inference is not capable of correcting the structure of the misleading phylogeny. In addition, AAF corrects every branch length by the same amount, which does not solve the problem caused by samples of different biases. Moreover, this correction depends on the estimation of sequencing depth and sequencing error rate, which complicates the problem. On the other hand, Skmer is able to adjust the bias between any pair of NGS samples differently, but it also requires to estimate sequencing depth and sequencing error rate first and then adjust the formula of Mash (Jaccard distance) [5] accordingly. Although this bias adjustment method works for simple dissimilarity measures such as Jaccard distance, adjusting the formula of more complicated background-adjusted methods such as *CVTree*[1], $d_{2}^{s}$[2], and $d_{2}^{*}$[2] can be a daunting, if not impossible, task.

Therefore, a method that can adjust the bias for alignment-free dissimilarity based on NGS samples without downsampling and without introducing new estimations such as sequencing depth or sequencing error rate is necessary. Since background-adjusted dissimilarity measures have been shown to outperform other methods for solving different problems ranging from evolutionary distance estimation [14] to virus-host interaction prediction [15], geographic location prediction [12], horizontal gene transfer detection [16], and metagenome and metatranscriptome comparison [10, 17], we focused on the bias adjustment for two background-adjusted dissimilarity measures $d_{2}^{s}$ and $d_{2}^{*}$ in this study. Nevertheless, our method can be naturally generalized to adjust the bias for other alignment-free methods.

## Results

### Alignemt-free methods overestimate distance between NGS samples

**Fig. 1:**
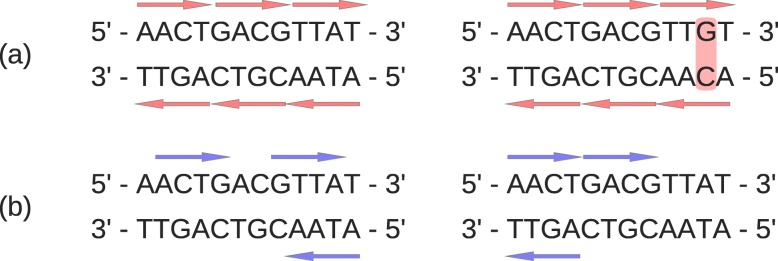
Bias caused by NGS sampling. **a** Two genomes that differ by only 1 bp, marked in red. Both of their NGS samples (red arrows) perfectly cover their genomes. **b** Two genomes that are exactly the same. Their NGS samples (blue arrows) only partially cover their genomes

The bias caused by NGS samples can be illustrated by a simplified example in Fig. 1. Figure 1a shows two fictitious 12-bp genomes that differ by 1 bp (A-T ⇔ G-C), and Fig. 1b shows two 12-bp genomes that are exactly the same. The dissimilarity measured by any reasonable alignment-free method between two genomes in Fig. 1b should be 0 and is thus smaller than the dissimilarity between two genomes in Fig. 1a. However, the dissimilarity between their NGS samples can show opposite results. For example, if the short reads (red arrows) in NGS samples fully cover the two genomes in Fig. 1a whereas the short reads (blue arrows) only partially cover the two genomes in Fig. 1b, it is clear that the dissimilarity based on the two NGS samples in Fig. 1b is greater than the dissimilarity based on the two NGS samples in Fig. 1a. This apparent contradiction can be explained by different biases of NGS samples caused by different sequencing depths.

Although Fig. 1 illustrates this bias by a simplified and extreme example, we used a real dataset of 21 primates from [18] and simulated NGS samples to show this bias. In our previous study [14], we calculated pairwise $d_{2}^{s}$ and $d_{2}^{*}$ using *K*=5 to *K*=14 where *K* is the length of the *k*mer with Markovian order *M*=*K*−2 for the background sequences between these 21 primate genomes and compared them with their pairwise evolutionary distances estimated by alignment-based methods. Our results showed that pairwise $d_{2}^{s}$ and $d_{2}^{*}$ with *K*=14 and *M*=12 are highly correlated with their evolutionary distances based on the alignments with Spearman correlation coefficients 0.979 for $d_{2}^{s}$ and 0.970 for $d_{2}^{*}$ (Additional file 1: Figures S1–S4).

**Fig. 2:**
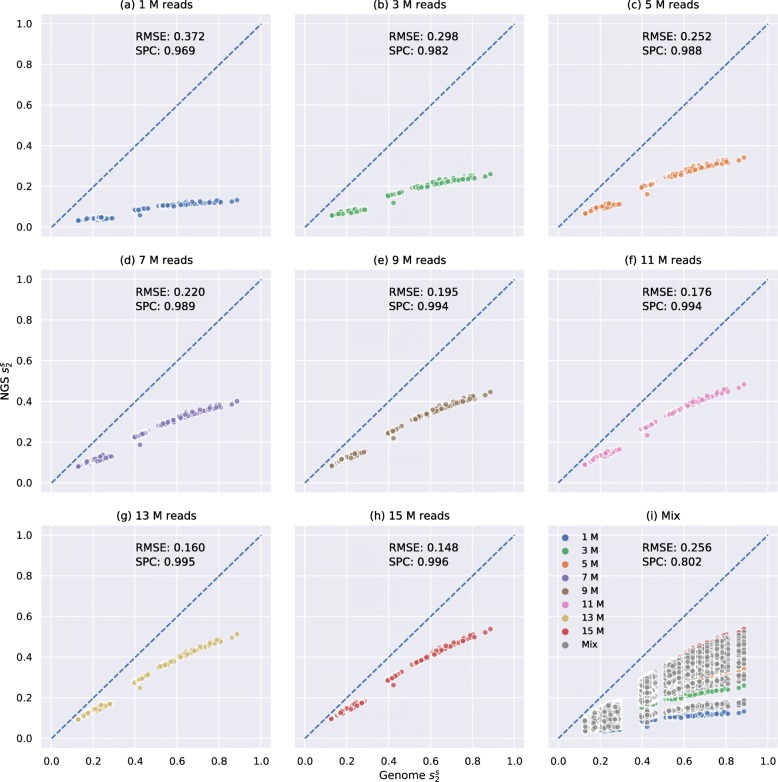
Relationship between pairwise $s_{2}^{s}$ estimated by primate genomes and NGS samples using *K*=14 and *M*=12 of different numbers of reads without bias adjustment. *X*-axis is the pairwise $s_{2}^{s}$ estimated by genomes, and *Y*-axis is the pairwise $s_{2}^{s}$ estimated based on NGS samples. **a**–**h** Relationship between $s_{2}^{s}$ estimated by primate genomes and $s_{2}^{s}$ estimated based on NGS samples of only 1 M, 3 M, 5 M, 7 M, 9 M, 11 M, 13 M, or 15 M reads, respectively. **i** Pairwise $s_{2}^{s}$ estimated based on mixed NGS samples. NGS samples of different numbers of reads are colored accordingly. “Mix” means two NGS samples have different numbers of reads (e.g., between 1 and 5 M or between 7 and 11 M) and is colored in gray. The root mean squared error (RMSE) and Spearman correlation coefficients (SPC) between pairwise $s_{2}^{s}$ estimated based on NGS samples and genomes are shown on each subplot

To study the influence of sequencing depths on $d_{2}^{s}$ and $d_{2}^{*}$, we simulated 8 NGS samples of different numbers of 150-bp Illumina reads (1 M, 3 M, 5 M, 7 M, 9 M, 11 M, 13 M, and 15 M) for each primate genome, corresponding to sequencing depths from 0.05× to 0.75× (see the “Methods” section). A total 8×21=168 NGS samples with different sequencing depths were generated and mixed together. We then calculated their pairwise $d_{2}^{s}$ and $d_{2}^{*}$ values and compared them with the pairwise $d_{2}^{s}$ and $d_{2}^{*}$ calculated based on their complete genomes. The result of $d_{2}^{s}$ using *K*=14 and *M*=12 is shown in Fig. 2. The results of $d_{2}^{s}$ using other *k*mer lengths and Markovian orders are shown in Additional file 1: Figure S5. The results of $d_{2}^{*}$ using different *k*mer lengths are shown in Additional file 1: Figures S6–S7. Both $d_{2}^{s}$ and $d_{2}^{*}$ have been transformed to their corresponding similarity measures where $s_{2}^{s} = 1 - 2 \times d_{2}^{s}$ and $s_{2}^{*} = 1 - 2 \times d_{2}^{*}$.

As shown in Fig. 2a–h, it is clear that $s_{2}^{s}$ estimated from NGS samples is lower than $s_{2}^{s}$ estimated from genomes as all scatter points are below the dashed blue line across the diagonal and the bias, visualized as the gap between the scatter points and the diagonal, decreases when the sequencing depth increases. In addition, Fig. 2a–h clearly illustrate that $s_{2}^{s}$ calculated based on NGS strongly correlates with $s_{2}^{s}$ calculated based on the whole genomes if all samples have the same sequencing depth even when the sequencing depth is as low as 0.05× (1 M). The Spearman correlation coefficients between $s_{2}^{s}$ based on NGS samples of 1 M reads and $s_{2}^{s}$ based on genomes is as high as 0.969. However, if not all samples have the same sequencing depth, the Spearman correlation coefficient dropped significantly even when we analyzed more number of reads in total, supported by comparing Figs. 2a and i. In Fig. 2a, all samples have only 1 M reads whereas in Fig. 2i, each sample has a different number of reads ranging from 1 to 15 M. The most likely reason is that if $s_{2}^{s}$ calculated between two NGS samples A and B of 15 M reads (Fig. 1h) is greater than $s_{2}^{s}$ calculated between two NGS samples C and D of 1 M reads (Fig. 1a), it does not necessarily mean the genome $s_{2}^{s}$ between A and B is greater than that between C and D. The reason is that samples of 15 M reads have smaller bias than samples of 1 M reads and thereby $s_{2}^{s}$ calculated from samples of 15 M reads will be generally greater than samples of 1 M reads regardless of their genome $s_{2}^{s}$. This observation supports our argument that bias caused by different sequencing depths markedly decrease the performance of alignment-free analysis based on NGS sequencing data. The same observation can be made for $d_{2}^{s}$ using different *k*mer lengths (Additional file 1: Figure S5) and for $d_{2}^{*}$ (Additional file 1: Figures S6–S7). A more detailed results of the “Mix” label in Fig. 2i was reported in Additional file 1: Figure S8a in which “Mix” was divided into more specific labels such as “1 M and 5 M”, “1 M and 15 M,” and “5 M and 15 M.”

To show that this bias is a common problem for all alignment-free methods, we did the same analysis for another state-of-the-art alignment-free method Mash [5] which is based on Jaccard distance. We first calculated pairwise Mash distances based on 21 primate genomes using *K*=14 (the same *k*mer length as we used for $d_{2}^{s}$ and $d_{2}^{*}$), *K*=21 (default *k*mer length for Mash), *K*=31 (maximum *k*mer length allowed by Mash), and sketch sizes *s*=10^3^,*s*=10^5^, and *s*=10^7^ and compared them with the pairwise evolutionary distances estimated by alignment-based methods. Additional file 1: Figure S9 shows that the pairwise Mash distances and the evolutionary distances have the highest Spearman correlation coefficient of 0.984 when using *K*=21 and *s*=10^7^.

We then chose the *k*mer length *K*=21 and sketch size *s*=10^7^ and compared Mash distances estimated from primate genomes and Mash distances estimated from primate NGS samples. The results are shown in Additional file 1: Figure S10, and Mash distance has been transformed to the corresponding Mash similarity that equals to 1 - Mash distance. Similar to $s_{2}^{*}$ and $s_{2}^{s}$, Mash similarity estimated from NGS samples is also lower than Mash similarity estimated from genomes, and this bias increases as the sequencing depth decreases as shown in Additional file 1: Figure S10a–h. As a consequence, the Spearman correlation coefficient (0.860) between Mash similarity based on genomes and Mash similarity based on NGS samples of 1 M to 15 M reads (Additional file 1: Figure S10i) is even lower than the corresponding Spearman correlation coefficient (0.943) based on NGS samples of only 1 M reads (Additional file 1: Figure S10a).

As aforementioned, one solution is to downsample all NGS samples to have the same number of reads as the smallest sample, which is 1 M reads in this example, as shown in Fig. 2a. This method does not adjust the bias of $s_{2}^{s}$ calculated based on NGS samples, but it controls that all samples have similar biases. The performance after downsampling is acceptable with Spearman correlation coefficient 0.969 (Fig. 2a) and is better than the performance without bias adjustment or downsampling (Fig. 2i). However, the vast majority of reads are discarded by downsampling, and thereby, much information is lost. For instance, in order to downsample a sample of 15 M reads to 1 M reads, we need to discard 93.3% of the reads in this sample.

### Bias adjustment by a neural network regression model

We characterize the bias adjustment process as a regression problem that predicts the dissimilarity based on genomes from the dissimilarity based on NGS samples and their biases. It can be clearly seen in Fig. 2 and Additional file 1: Figures S8a and S10 that the alignment-free dissimilarity between any pair of NGS samples *d*(*A*_NGS_,*B*_NGS_) is determined by the alignment-free dissimilarity based on their genomes *d*(*A*_*G*_,*B*_*G*_) and the bias caused by each NGS sample Bias(*A*_NGS_) and Bias(*B*_NGS_):

$$ d(A_{\text{NGS}}, B_{\text{NGS}}) = F(d(A_{G}, B_{G}), \text{Bias}(A_{\text{NGS}}), \text{Bias}(B_{\text{NGS}})) $$

In other words, if we know the function *F*, alignment-free dissimilarity between a pair of NGS samples, and their corresponding biases, then the alignment-free dissimilarity based on their genomes which is not biased by the sequencing depths in NGS samples can be predicted. Although it is hard to infer a closed-form formula for function *F* for background-adjusted methods such as *CVTree*[1], $d_{2}^{s}$[2], and $d_{2}^{*}$[2], a neural network regression model can be trained to approximate it, see the “Methods” section for more details about the definition of Bias(*A*_NGS_),Bias(*B*_NGS_), and model training and evaluation.

### The correlation between the adjusted dissimilarity measures based on NGS samples and genomes of 21 primates is markedly increased

**Fig. 3:**
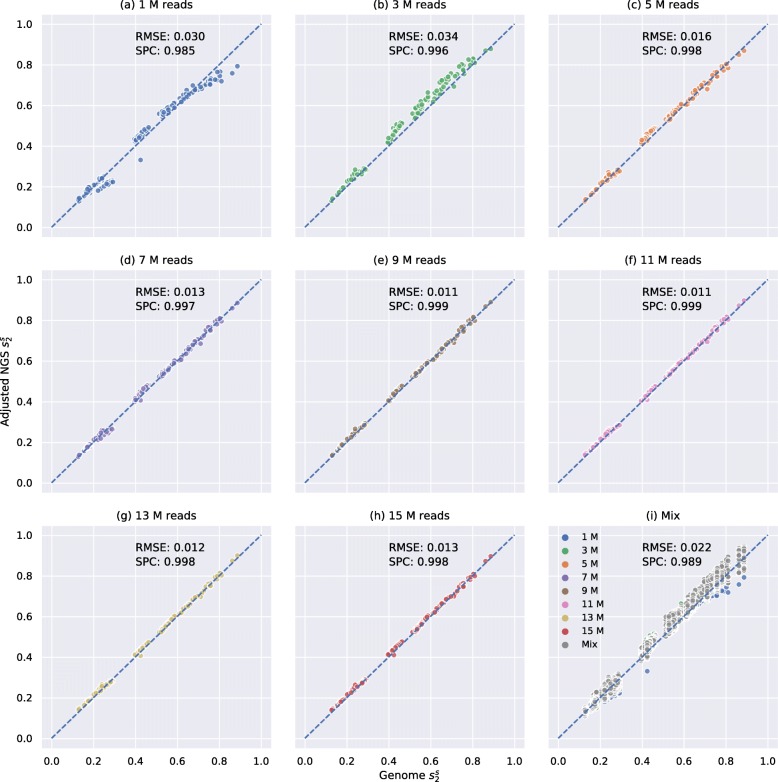
Relationship between pairwise $s_{2}^{s}$ estimated by primate genomes and NGS samples using *K*=14 and *M*=12 of different numbers of reads with bias adjustment. X-axis is the pairwise $s_{2}^{s}$ estimated by genomes and Y-axis is the pairwise $s_{2}^{s}$ estimated based on NGS samples after bias adjustment. **a**–**h** show the relationship between $s_{2}^{s}$ estimated by primate genomes and adjusted $s_{2}^{s}$ based on NGS samples of only 1 M, 3M, 5 M, 7M, 9 M, 11 M, 13 M or 15 M reads, respectively. **i** shows pairwise adjusted $s_{2}^{s}$ based on mixed NGS samples. NGS samples of different numbers of reads are colored accordingly. ‘Mix’ means two NGS samples have different numbers of reads (e.g., between 1 M and 5 M or between 7 M and 11 M) and is colored in gray. The root mean squared error (RMSE) and Spearman correlation coefficients (SPC) between pairwise $s_{2}^{s}$ estimated based on NGS samples and genomes are shown on each subplot

Two neural network regression models were trained using the 21 primate dataset for $d_{2}^{s}$ and $d_{2}^{*}$ separately and used to adjust the bias of primate NGS samples (see the “Methods” section). Using the resulting neural network model, we adjusted the pairwise $d_{2}^{s}$ and $d_{2}^{*}$ dissimilarity measures described in the above section. We then calculated the Spearman correlation between the adjusted dissimilarity measures with the corresponding values using the whole genomes. The correlations between adjusted $d_{2}^{s}$ (adjusted $d_{2}^{*}$) and their genome $d_{2}^{s}$ ($d_{2}^{*}$) were calculated, and the results of $d_{2}^{s}$ using *K*=14 and *M*=12 were transformed to $s_{2}^{s}$ and shown in Fig. 3 and Additional file 1: Figure S8b in which “Mix” was divided into more specific labels such as “1 M and 5 M.” The results of bias adjustment for $d_{2}^{s}$ using other *k*mer lengths and Markovian orders were shown in Additional file 1: Figure S11. The results of bias adjustment for $d_{2}^{*}$ using different *k*mer lengths were transformed to $s_{2}^{*}$ and shown in Additional file 1: Figures S12–S13. By comparing Figs. 2 and 3, we can conclude that our model successfully adjusted the bias between NGS $s_{2}^{s}$ and genome $s_{2}^{s}$, supported by the observation that most scatter points fall on the diagonal in Fig. 3. In addition, the root mean squared error was decreased, and the Spearman correlation coefficient was increased after bias adjustment. More importantly, Fig. 3 and Additional file 1: Figures S8 and S11–13 revealed that our bias adjustment method works for both $d_{2}^{s}$ and $d_{2}^{*}$, regardless of the chosen *k*mer length, Markovian order, or sequencing depth. It should be noticed that our bias adjustment model is capable of increasing the Spearman correlation coefficients even when all samples have the same number of reads by comparing Fig. 3a–h to the corresponding Fig. 2a–h. A possible explanation could be that the same number of reads cannot guarantee the same sequencing depth if genome lengths are different. Moreover, the bias might also be caused by other factors such as sequencing errors and sequencing bias that cannot be controlled by downsampling. Therefore, we suggest always using our model to adjust the bias in the alignment-free analysis based on NGS sequencing data even when each sample has a similar number of reads to achieve better performance.

We also evaluated the performance of Skmer [8] on the same primate dataset using *k*mer length *K*=21 and sketch size *s*=10^7^, which is a recent alignment-free method that corrects the formula of Mash distance based on NGS samples by estimating the sequencing depth and sequencing error rate. The relationship between the Skemr distances using the whole genomes and the Skmer distances using the NGS samples are shown in Additional file 1: Figure S14, and Skmer distance has been transformed to the corresponding Skmer similarity that equals to 1 - Skmer distance. By comparing Additional file 1: Figure S10a–h to the corresponding Additional file 1: Figure S14a–h, we can see that Skmer adjusted Mash similarity by increasing its value estimated from NGS samples to compensate for the low sequencing depths and sequencing errors as more points fall on diagonals in Additional file 1: Figure S14. However, Skmer decreased the Spearman correlation coefficients, especially when NGS samples have different sequencing depths by comparing the coefficient of Mash (0.860) in Additional file 1: Figure S10i and the coefficient of Skmer (0.766) in Additional file 1: Figure S14i. A possible explanation could be that the formula that Skmer used in [8] to correct Mash distance by estimating sequencing depth and sequencing error rate is not accurate when two NGS samples have different sequencing depths. As a comparison, Fig. 3 and Additional file 1: Figures S10 and S12 demonstrated that adjusted $d_{2}^{s}$ and $d_{2}^{*}$ outperform Mash and Skmer in all circumstances, especially when the sequencing depth is low (< 9 M reads) or samples have different sequencing depths.

### The correlation between the adjusted dissimilarity measures based on NGS samples and genomes of 28 mammals is markedly increased

**Fig. 4:**
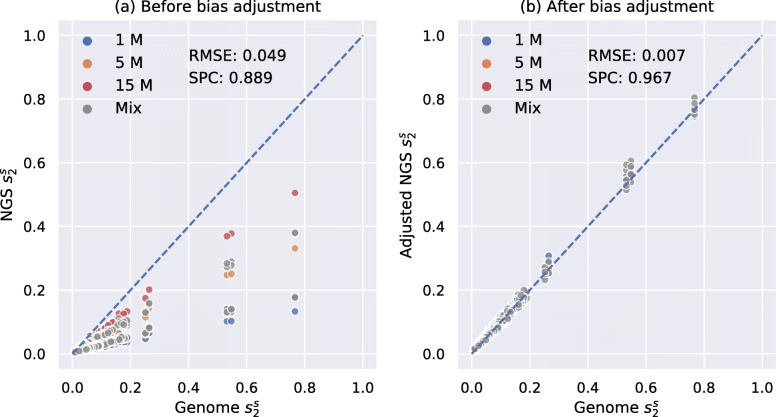
Relationship between pairwise $s_{2}^{s}$ estimated using *K*=14 and *M*=12 based on 28 mammalian genomes and NGS samples of different numbers of reads. **a** shows the relationship before bias adjustment. **b** shows the relationship after bias adjustment for NGS $s_{2}^{s}$. The root mean squared error (RMSE) was decreased and the Spearman correlation coeffient (SPC) between pairwise genome $s_{2}^{s}$ and NGS $s_{2}^{s}$ was increased after bias adjustment

We tested our model for $d_{2}^{s}$ bias adjustment on an independent dataset of 28 mammals from [19]. In our previous study [14], we have calculated pairwise $d_{2}^{s}$ using *K*=14 and *M*=12 between these 28 mammalian genomes and showed that their pairwise $d_{2}^{s}$ are highly correlated with their pairwise evolutionary distances estimated by alignment-based methods with Spearman correlation coefficient of 0.927, and the result is shown in Additional file 1: Figure S15. We simulated 3 NGS samples of different numbers of 150-bp Illumina reads (1 M, 5 M, and 15 M) for each mammalian genome, corresponding to sequencing depths from 0.05× to 0.75×, resulting in a total of 28×3=84 samples (see the “Methods” section). We then calculated pairwise $d_{2}^{s}$ between all 84 NGS samples, adjusted them using our neural network model and then compared them with the pairwise $d_{2}^{s}$ calculated from their complete genomes. The result was transformed to $s_{2}^{s}$ and shown in Fig. 4. It can be clearly seen in Fig. 4a that pairwise NGS $d_{2}^{s}$ was overestimated before adjustment since all scatter points were below the diagonal whereas most scatter points after bias adjustment in Fig. 4b fall on the diagonal, which proved that our model has successfully adjusted the bias of $d_{2}^{s}$. In addition, the root mean squared error was decreased, and the Spearman correlation coefficient was increased after bias adjustment.

We next tested the performance of Mash and Skmer on the same mammalian dataset. We first calculated pairwise Mash distances based on the 28 mammalian genomes using *K*=14,*K*=21, and *K*=31 and sketch size *s*=10^3^,*s*=10^5^, and *s*=10^7^ and compared them with the pairwise evolutionary distances estimated by alignment-based methods. Additional file 1: Figure S16 shows that pairwise Mash distances and the evolutionary distances have the highest Spearman correlation coefficient of 0.943 when using *K*=31 and *s*=10^7^. We then chose *k*mer length *K*=31 and sketch size *s*=10^7^ and compared Mash distance and Skmer distance estimated from mammalian genomes and estimated from NGS samples. The results are shown in Additional file 1: Figure S17. The Spearman correlation coefficient (0.967) between adjusted $d_{2}^{s}$ based on NGS samples and genomes is significantly higher than that for Mash (0.789) and Skmer (0.688).

### The accuracy on predicting continental origins of white oak NGS samples using *k*-NN is markedly increased

We tested our model for $d_{2}^{*}$ bias adjustment on a dataset of 92 white oak NGS samples collected from 3 continents (North America, Asia, and Europe). In our previous study [12], we downsampled each sample to 3 different sequencing quantities (50 Mbp, 100 Mbp, and 300 Mbp), corresponding to sequencing depths from 0.07× to 0.42×. At each sequencing quantity, samples were randomly divided into reference and query set, and for each sample in the query set, we found its *k*-nearest neighbors (*k*-NN) measured by $d_{2}^{*}$ with *K*=12 and *M*=10 in the reference set and predicted its continental origin by a majority vote approach (see the “Methods” section). *k*-NN accuracy at all these 3 sequencing quantities is shown in Additional file 1: Table S1, and it can be clearly seen that the accuracy increases with sequencing quantity.

We randomly selected 30 samples from 50 Mbp dataset, 31 samples from 100 Mbp dataset, and 31 samples from 300 Mbp dataset and mixed them together to build a new dataset of NGS samples with different sequencing quantities. We predicted the continental origins of the samples in the query set using the same method, and results are shown at the top of Table 1. Unsurprisingly, the accuracy was lower than even when we downsampled all samples to 50 Mbp (Additional file 1: Table S1) because a sample from Asia might have smaller $d_{2}^{*}$ to a sample from Europe of 300 Mbp than another sample from Asia of 50 Mbp, and it is likely to be misclassified. We used our model for $d_{2}^{*}$ to adjust the bias and predicted their continental origins again based on the dissimilarity after bias adjustment, and the prediction accuracy is shown at the bottom of Table 1. It is clear that our bias adjustment model was capable of increasing the accuracy markedly, especially when the reference size is small. It should be noticed that the accuracy after bias adjustment is higher than the accuracy by downsampling all samples to 50 Mbp or 100 Mbp, and it is comparable to the accuracy when all samples are of 300 Mbp, which shows that bias adjustment can achieve better performance than downsampling since the vast majority of reads are discarded by downsampling whereas bias adjustment still analyzes all the reads.

**Table 1:** Prediction accuracy using *k*-NN on 92 white oak datasets of mixed sequence quantity based on $d_{2}^{*}$ before and after bias adjustment for different query sizes, reference sizes, and different numbers of neighbors *k* used

Query size	Reference size	*k* = 1	*k* = 2	*k* = 3	*k* = 4	*k* =5	*k* = 6	*k* = 7	*k* = 8	*k* = 9	*k* = 10
Before bias adjustment											
1	91	0.97	0.97	0.97	1.00	1.00	1.00	0.98	0.95	0.91	0.91
17	75	0.98	0.98	0.96	0.99	0.96	0.98	0.96	0.95	0.91	0.91
32	60	0.97	0.97	0.94	0.96	0.94	0.95	0.91	0.92	0.88	0.89
47	45	0.95	0.95	0.93	0.94	0.91	0.91	0.88	0.89	0.87	0.88
62	30	0.93	0.93	0.88	0.89	0.85	0.87	0.83	0.84	0.82	0.81
77	15	0.84	0.84	0.77	0.78	0.75	0.74	0.69	0.70	0.67	0.65
After bias adjustment											
1	91	1.00	1.00	1.00	1.00	1.00	1.00	1.00	1.00	1.00	1.00
17	75	1.00	1.00	1.00	1.00	1.00	1.00	0.99	1.00	1.00	1.00
32	60	1.00	1.00	1.00	1.00	0.99	1.00	0.99	1.00	0.99	0.99
47	45	1.00	1.00	0.99	1.00	0.99	0.99	0.98	0.99	0.97	0.98
62	30	0.99	0.99	0.97	0.97	0.94	0.95	0.92	0.93	0.90	0.91
77	15	0.96	0.96	0.92	0.93	0.87	0.87	0.81	0.79	0.74	0.70

For each query sizes and reference sizes, the dataset was randomly split 100 times and an average prediction accuracy was calculated over 100 splits

The prediction accuracy of Mash and Skmer was tested on the same oak dataset using *K*=12,*K*=21, and *K*=31 and sketch size *s*=10^3^,*s*=10^5^, and *s*=10^7^, and results are shown in Additional file 1: Tables S2 and S3, respectively. It is clear that the adjusted $d_{2}^{*}$ has higher prediction accuracy than Mash and Skmer, especially when the reference size is small. For instance, when there are only 15 samples in the reference set, the adjusted $d_{2}^{*}$ can still achieve an average prediction accuracy of 0.96 whereas the highest average prediction accuracies of Mash and Skmer using *K*=31 and *s*=10^7^ are 0.64 and 0.73, respectively.

### The prediction accuracy of geographic origin at finer scales for white oak NGS samples is markedly increased

**Fig. 5:**
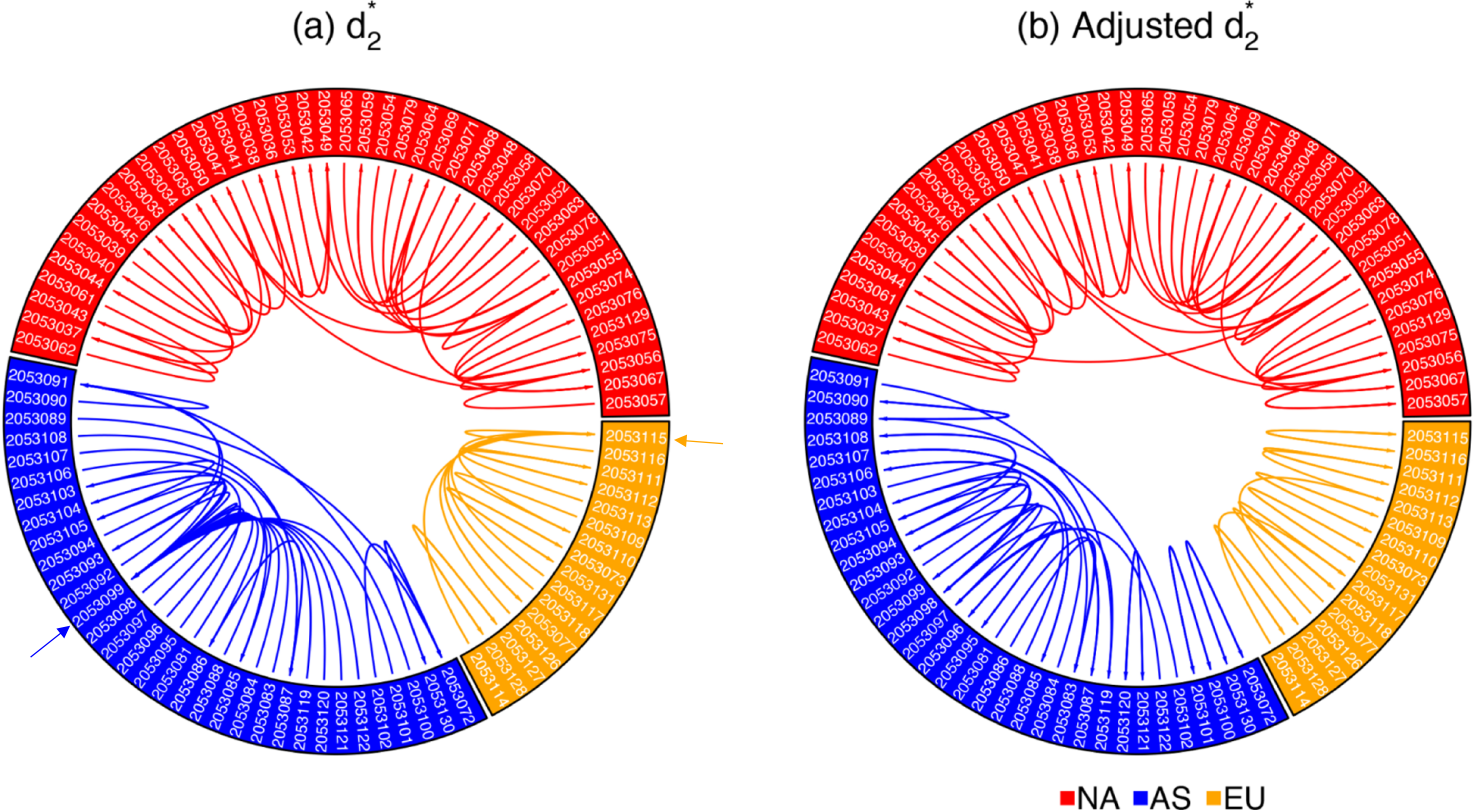
The circular plots of 92 white oak tree samples based on $d_{2}^{*}$ with *K*=12 and *M*=10 before and after bias adjustment. Different sectors correspond to different continents, with North America (NA) in red, Europe (EU) in orange, and Asia (AS) in blue. Within each sector, samples are sorted by their longitude, so that samples that are geographically close are also close to each other in the figure. The most similar tree sample to each sample is linked

Without downsampling, we calculated the pairwise $d_{2}^{*}$ using *K*=12 and Markovian order *M*=10 between all 92 white oak tree NGS samples with sequencing quantity ranging from 379 to 1852 Mbp, corresponding to sequencing depths from 0.53× to 2.59×. For each sample, we first found its closest sample according to $d_{2}^{*}$ and linked them together as shown in Fig. 5a. Although the most similar sample to each sample according to $d_{2}^{*}$ is from the same continent-of-origin, it does not perform well at finer geographic scales. It is clear that there are 2 sink nodes SRR2053099 (blue arrow) and SRR2053115 (orange arrow) in Fig. 5a. According to $d_{2}^{*}$, SRR2053099 was predicted as the most similar sample to 19 out of 31 Asian samples, and SRR2053115 was predicted as the most similar sample to 8 out of 16 European samples. The reason is that SRR2053099 (1414 Mbp) is one of the largest samples among all samples from Asia and SRR2053115 (1852 Mbp) is the largest sample among all samples from Europe, so they have the smallest biases in the samples from Asia and Europe, respectively. Therefore, they are more likely to be predicted as the closest samples to other samples according to $d_{2}^{*}$.

We adjusted the biases of $d_{2}^{*}$ using Afann, and the results are shown in Fig. 5b. It can be clearly seen that there is no sink node such as SRR2053099 and SRR2053115 in Fig. 5a, which proves that the adjusted $d_{2}^{*}$ is not biased by sequencing depth. In order to show that bias adjustment can improve the prediction accuracy at finer geographic scales, we calculated the average distance between each sample and its closest sample according to $d_{2}^{*}$ before and after bias adjustment. In Fig. 5, all samples are sorted by their longitude, so we define the distance between each sample and its closest sample based on their distance in the circular plots. For each sample, the minimum distance should be 1 if and only if its closest sample according to $d_{2}^{*}$ is next to it in the circular plots. The average distances between all 92 samples and their closest samples are 7.42 before bias adjustment and 5.85 after bias adjustment. A paired sample *t* test showed that the average distance after bias adjustment is significantly lower than before adjustment with a *p* value of 0.023. Therefore, although $d_{2}^{*}$ based on NGS samples without downsampling or bias adjustment can successfully predict their continental origins, we proved that bias adjustment can further increase the prediction accuracy at finer geographic scales.

### The correlation between the adjusted dissimilarity measures based on NGS samples and genomes of 67 vertebrates is markedly increased

Since our previous datasets all consist of sequencing reads coming from closely related species, we constructed a dataset that contains samples from 67 vertebrates to evaluate the performance of our method on diverse datasets. It contains vertebrate genomes of 67 species from 5 different classes including fish, amphibians, reptiles, birds, and mammals. Among these 67 vertebrate genomes, we randomly selected 23, 22, and 22 genomes and simulated their NGS samples of 1 M, 5 M, and 15 M 150-bp Illumina reads, respectively, and mixed all 67 NGS samples together. The sequencing depths of all NGS samples range from 0.024× to 3.49×.

**Fig. 6:**
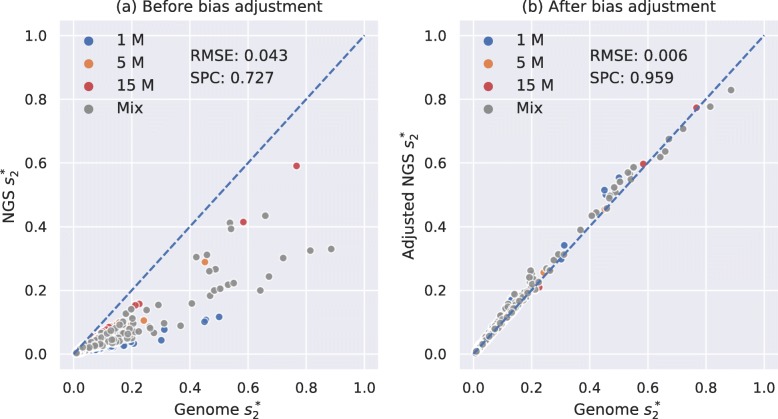
Relationship between pairwise $s_{2}^{*}$ estimated using *K*=14 and *M*=12 based on 67 vertebrate genomes and NGS samples of different numbers of reads. **a** The relationship before bias adjustment. **b** The relationship after bias adjustment for NGS $s_{2}^{*}$. The root mean squared error was decreased, and the Spearman correlation coefficient between pairwise genome $s_{2}^{*}$ and NGS $s_{2}^{*}$ was increased after bias adjustment

We then calculated pairwise $d_{2}^{*}$ and $d_{2}^{s}$ using *K*=14 and *M*=12 between all 67 NGS samples with and without bias adjustment and compared them with the pairwise $d_{2}^{*}$ and $d_{2}^{s}$ calculated from their complete genomes. The result of $d_{2}^{*}$ was transformed to $s_{2}^{*}$ and shown in Fig 6. It can be demonstrated from Fig. 6 that our method markedly decreased the root mean squared error and increased the Spearman correlation coefficient from 0.727 to 0.959. The result of $d_{2}^{s}$ was transformed to $s_{2}^{s}$ and shown in Additional file 1: Figure S18, and the bias adjustment of $d_{2}^{s}$ increased the Spearman correlation coefficient from 0.701 to 0.935.

The performance of Mash and Skmer using *K*=31 and *s*=10^7^ was tested on the same vertebrate dataset and shown in Additional file 1: Figure S19. The Spearman correlation coefficients between adjusted $d_{2}^{*}$ (0.959) and adjusted $d_{2}^{s}$ (0.935) based on vertebrate NGS samples and genomes are significantly higher than that for Mash (0.747) and Skmer (0.735).

### Running time and memory

Although background-adjusted alignment-free methods such as *CVTree* [1], $d_{2}^{s}$ [2], and $d_{2}^{*}$ [2] have been shown to achieve better performance than simple *Manhattan* and *Euclidean* distances [10, 12, 14, 16, 17], their applications have been limited due to the high time and memory cost in the random background removing step. To overcome this bottleneck, we improved the speed and memory usage of background-adjusted methods in Afann by hashing, multi-threading, and vectorization and compared it with our previous program Cafe [14].

Both tools were used to calculate the pairwise $d_{2}^{s}, d_{2}^{*}$, and *CVTree* among the white oak datasets of 92 NGS samples with 300 Mbp sequencing quantity using *K*=12 and *M*=10 and among the primate dataset of 21 genomes using *K*=14 and *M*=12. Comparisons of time and memory based on white oak datasets and primate dataset were shown in Table 2 and Additional file 1: Table S4, respectively. The total time was divided into *k*mer counting time and dissimilarity calculation time. It can be clearly seen in Table 2 that the total speedup ratio of Afann is around 100× for all 3 background-adjusted methods whereas the memory usage is only one fifth of the memory of Cafe. Afann also supports fast calculation mode for $d_{2}^{*}$ and *CVTree* which further increases the calculation speed by using more memory. The memory usage is *O*(4^*K*^) for normal mode and *O*(*N*×4^*K*^) for the fast mode where *K* is the *k*mer length and *N* is the number of samples. It should be noticed that the counting time is *O*(*N*×4^*K*^) whereas the calculation time is *O*(*N*^2^×4^*K*^); the total speedup ratio will thereby be close to the speedup ratio of dissimilarity calculation as the number of samples *N* increases. We suggest using fast mode when memory allows. For example, it is common to compare the pairwise dissimilarity among thousands of bacterial genomes using small *k*mer length 5 or 6 which does not require much memory, then the speedup ratio of fast mode can be more than 5000×.

**Table 2:** *k*mer counting time, dissimilarity calculation time, and total time as well as memory usage used by Cafe and Afann to calculate the pairwise $d_{2}^{s}, d_{2}^{*}$, and *CVTree* using *K*=12 and *M*=10 among a dataset of 92 white oak NGS samples of 300 Mbp

	Counting (min)	Calculation (min)	Total time (min)	Memory (Mb)
Cafe-$d_{2}^{s}$	450.2	4260.2	4710.4	1916
Afann-$d_{2}^{s}$	21.9	31.2	53.1	449
Cafe-$d_{2}^{*}$	450.2	4224.1	4764.3	1928
Afann-$d_{2}^{*}$	21.9	14.2	36.1	304
Afann-$d_{2}^{*}$-fast	21.9	0.3	22.2	11953
Cafe-*CVTree*	450.2	4295.4	4745.6	1960
Afann-*CVTree*	21.9	14.1	36.0	304
Afann-*CVTree*-fast	21.9	0.3	22.2	11953
Mash ^min^	21.5	0.1	21.5	3
Mash ^opt^	125.6	25.5	151.1	20830
Skmer ^min^	NA	NA	111.9	565
Skmer ^opt^	NA	NA	656.9	2556

Afann-$d_{2}^{*}$-fast and Afann-*CVTree*-fast stand for the fast mode of $d_{2}^{*}$ and *CVTree* supported in Afann. Running time and memory usage of Mash and Skmer were also included. Mash ^min^ and Skmer ^min^ used *K*=12 and *s*=10^3^ which require the minimum computing power. Mash ^opt^ and Skmer ^opt^ used *K*=31 and *s*=10^7^ which have the optimal performance among Mash and Skmer using different combinations of *k*mer lengths and sketch sizes as shown in Additional file 1: Table S2 and Table S3

The running time and memory usage of other fast alignment-free tools Mash [5] and Skmer [8] on the same oak NGS and primate genome datasets were also calculated and reported in Table 2 and Additional file 1: Table S4, respectively. The running time and memory usage of an alignment-free genome comparison tool FFP [3] on the primate genome dataset using *K*=16 as suggested in [3] were reported in Additional file 1: Table S4. It should be noticed that the running time of Mash and Skmer using *K*=12 nad *s*=10^3^ for oak NGS dataset and *K*=14 and *s*=10^3^ for primate genome dataset was only included to test their speed when using the same *k*mer length as $d_{2}^{*}$ and $d_{2}^{s}$. In practice, *k*mer length shorter than 21 is not recommended for Mash and Skmer [8]. We can see in Table 2 and Additional file 1: Table S4 that Cafe calculates $d_{2}^{s}, d_{2}^{*}$, and *CVTree* much slower than Mash, Skmer, and FFP whereas Afann is capable of calculating $d_{2}^{s}, d_{2}^{*}$, and *CVTree* in a comparable amount of time as Mash, Skmer, and FFP. The pairwise dissimilarity measures among primate genomes calculated by different methods were compared with their evolutionary distances estimated by alignment-based methods in [18] and shown in Additional file 1: Figure S20. All dissimilarity measures except FFP are highly correlated with the evolutionary distance with Spearman correlation coefficients higher than 0.95 which demonstrated the applicability of alignment-free methods in genome comparisons. However, our evaluations based on different independent datasets in previous sections showed that Afann significantly outperforms others in comparing NGS samples.

## Discussion

Alignment-free sequence comparison methods, especially *k*mer-based methods have been widely used in NGS analysis without assembly or alignment. However, several studies have revealed that the alignment-free dissimilarity calculated based on NGS samples tends to be over-estimated compared to the alignment-free dissimilarity calculated based on their genomes caused by the stochastic distribution of short reads [4, 8, 12]. In this study, we showed that this bias could significantly decrease the performance of alignment-free analysis based on NGS samples of different sequencing depths by investigating four independent datasets. For the primate, mammalian, and vertebrate datasets, the correlation between pairwise NGS dissimilarity and pairwise genome dissimilarity dropped markedly if NGS samples of different numbers of reads were mixed together. For the white oak dataset, the *k*-NN prediction accuracy of their continental origins based on a dataset of samples with 50 M, 100 M, and 300 M sequencing quantity is even much lower than the accuracy based on a dataset of all samples with only 50 M sequencing quantity.

This problem was previously solved by downsampling [12] or modifying the specific dissimilarity formula by estimating the sequencing depth and sequencing error rate [4, 8]. However, the first method discards the vast majority of reads that could have been informative, and the second method depends on the estimation of sequencing depth and sequencing error rate and cannot be generalized to adjust the bias for other alignment-free methods calculated by a different formula. In addition, it can be extremely hard to adjust the formula of several complicated background-adjusted methods such as *CVTree*[1], $d_{2}^{s}$ [2], and $d_{2}^{*}$[2].

Therefore, we introduced a de novo method in this study to adjust bias without estimating the sequencing depth or sequencing error rate explicitly by defining $\text {Bias}(A_{\text {NGS}}) = d(A_{\text {NGS}}, A_{\text {NGS}}^{R})$. This bias estimator will increase as the sequencing depth decreases or sequencing error rate increases and thus implicitly capture information from sequencing depth and sequencing error rate. Therefore, bias adjustment could be characterized as a regression problem that uses *d*(*A*_NGS_,*B*_NGS_),Bias(*A*_NGS_), and Bias(*B*_NGS_) to predict *d*(*A*_*G*_,*B*_*G*_). Two neural network regression models were trained for $d_{2}^{s}$ and $d_{2}^{*}$ separately using the primate dataset. Our results showed that bias was successfully adjusted for NGS samples of different sequencing depth and calculated by using different *k*mer length, supported by the large improvement of root mean squared error and Spearman correlation coefficient between dissimilarity based on NGS samples after bias adjustment and dissimilarity based on genomes.

Without changing any parameters, the performance of our models was tested on 3 independent datasets. A 28 mammalian dataset was used to test our bias adjustment model for $d_{2}^{s}$. Each genome was simulated to 3 NGS samples of 1 M, 5 M, and 15 M Illumina 150-bp reads and mixed together. Pairwise $d_{2}^{s}$ values using *K*=14 and *M*=12 between all 84 samples were calculated without and with bias adjustment and compared with pairwise genome $d_{2}^{s}$. The results showed that our method successfully adjusted the bias and greatly improved both root mean squared error and Spearman correlation coefficient. A 92 white oak dataset was used to test our bias adjustment model for $d_{2}^{*}$. We randomly selected 30 samples from the 50 Mbp dataset, 31 samples from the 100 Mbp dataset, and 31 samples from the 300 Mbp dataset and mixed them together. Pairwise $d_{2}^{*}$ values using *K*=12 and *M*=10 between all 92 samples were calculated without and with bias adjustment, and *k*-NN was used to predict the continental origins of test samples based on $d_{2}^{*}$. Our result showed that the prediction accuracy of the mixed dataset using *k*-NN is even lower than that using a dataset of all 50 Mbp samples before bias adjustment. After bias adjustment, the *k*-NN accuracy markedly increased and was comparable to the accuracy based on a dataset of all 300 Mbp samples. In addition, we proved that bias adjustment could increase the accuracy of prediction not only at continental level but also at finer geographic scales. At last, a 67 vertebrate dataset consisting of species from 5 different classes was used to demonstrate the reliability of our method on datasets composed of diverse species. In all datasets, our method outperformed other alignment-free methods including Mash [5] and Skmer [8] in terms of the Spearman correlation coefficient and prediction accuracy.

It should be noticed that while our bias adjustment method is capable of successfully predicting the alignment-free dissimilarity based on genomes regardless of the chosen *k*mer length, it is, nevertheless, important to choose a proper *k*mer length so that the alignment-free dissimilarity based on genomes is highly correlated with their evolutionary distance. For instance, while Additional file 1: Figure S11 shows that our model successfully adjusted the bias of $d_{2}^{s}$ using *K*=5 to *K*=13 based on primate NGS samples, it can be clearly seen in Additional file 1: Figure S4 that the correlation between $d_{2}^{s}$ based on primate genomes and their evolutionary distance is lower than 0.90 when *K*<10 is used. Therefore, even if our model can adjust the bias of *K*<10, the performance might not increase as we expected. There have been several investigations into the choice of optimal *k*mer length [2, 3, 20, 21]. In practice, shorter *k*mers are optimal when sequences are short or obviously different, whereas longer *k*mers should be used when sequences are from very closely related species in order to reduce the probability that a *k*mer commonly appear in a sequence by chance [2, 3, 5, 20].

In conclusion, our study showed that bias adjustment is a necessary step to increase the performance of alignment-free methods based on NGS samples. Although our models were trained only on the primate dataset, it was able to adjust the bias for independent mammalian, vertebrate, and white oak datasets, which proved that our model is generalizable to adjust the bias of NGS samples from different species, sequencing depths using different *k*mer lengths. Future work could train our models using more samples from different species with more variant sequencing depths to further improve the performance. In addition, since our bias adjustment method only relies on the alignment-free dissimilarity calculated between *A*_NGS_ and $A_{\text {NGS}}^{R}$ without estimating sequencing depths and sequencing error rates or considering the actual formula of the dissimilarity measures, it can be easily generalized to adjust the bias of all alignment-free dissimilarity measures by training their own regression models. In this paper, we showed the success of our bias adjustment model for two background-adjusted methods $d_{2}^{s}$ and $d_{2}^{*}$; the framework developed in this paper can be easily adapted to adjust bias in other alignment-free dissimilarity measures.

## Conclusion

Afann is a fast tool to calculate background-adjusted alignment-free dissimilarity measures *CVTree*, $d_{2}^{*}$, and $d_{2}^{s}$ between genome sequences and NGS samples. In addition, it can adjust the biases caused by NGS samples of different sequencing depths for $d_{2}^{*}$ and $d_{2}^{s}$ without downsampling or estimating the sequencing depth. Our results showed that the adjusted $d_{2}^{*}$ and $d_{2}^{s}$ are not biased by sequencing depth and can significantly increase the performance of studies based on NGS samples.

## Methods

See Appendix A of Additional file 1 for more details about different alignment-free dissimilarity measures including *CVTree*[1], $d_{2}^{s}$ [2], and $d_{2}^{*}$[2].

### Genomic datasets and simulation of NGS samples

The primate dataset consists of 21 complete primate genome sequences downloaded from NCBI. In [18], the author estimated the evolutionary distances among 186 primates based on the alignment of 54 nuclear gene regions. In our previous study [14], we found 21 complete representative genomes on NCBI among these 186 primates and demonstrated that their pairwise $d_{2}^{s}$ and $d_{2}^{*}$ with *K*=14 and *M*=12 are highly correlated with their evolutionary distances estimated in [18]. The species names, assembly accession numbers, and total sequence lengths of these 21 primate genomes are shown in Additional file 1: Table S5. For each genome, we used ART [22] to simulate different numbers of Illumina HiSeq 2500 reads of length 150 bp with default sequencing error profile. We produced 8 different datasets with 1 M, 3 M, 5 M, 7 M, 9 M, 11 M, 13 M, and 15 M reads for each NGS sample. We then mixed all 21×8=168 NGS samples to generate a new dataset of primate NGS samples.

Similarly, the mammalian dataset consists of 28 complete vertebrate genome sequences downloaded from NCBI, with evolutionary distances calculated by the alignment-based method in [19]. The species names, assembly accession numbers, and total sequence lengths of these 28 mammalian genomes are shown in Additional file 1: Table S6. For each genome, we used ART [22] to simulate different numbers of Illumina HiSeq 2500 reads of length 150 bp with default sequencing error profile. We produced 3 different datasets with 1 M, 5 M, and 15 M reads for each NGS sample. We then mixed all 28×3=84 NGS samples to generate a new dataset of mammalian NGS samples.

The white oak tree dataset consists of whole-genome shotgun (WGS) sequencing data of 92 white oaks from North America, Europe, and Asia with sequencing quantity ranging from 379 to 1852 Mbp from NCBI BioProject PRJNA269970 [23]. The run accession numbers, number of bases, and continental origins for these 92 samples are shown in Additional file 1: Table S7. We downsampled all 92 samples to produce 3 different datasets with 50 Mbp, 100 Mbp, and 300 Mbp, for each sample, respectively. Then, we randomly chose 30 samples from the 50 Mbp dataset, 31 samples from the 100 Mbp dataset, and 31 samples from the 300 Mbp and mixed them together to generate a new dataset of 92 NGS samples with different sequencing quantities. All samples were divided into 3 geographic categories (North America, Europe, and Asia) based on their continental origins.

The vertebrate dataset consists of 67 complete vertebrate genome sequences downloaded from NCBI. The species are from 5 different classes, including 15 fish, 7 amphibians, 15 reptiles, 15 birds, and 15 mammals. All 15 species were randomly selected from the corresponding classes except for amphibian where there are only 7 amphibian complete genome sequences available on NCBI, and thereby, they were all included in the dataset. The species names, classes, assembly accession numbers, and total sequence lengths of these 67 vertebrate genomes are shown in Additional file 1: Table S8. Among these 67 vertebrate genomes, we randomly selected 23, 22, and 22 genomes and simulated their NGS samples of 1 M, 5 M, and 15 M 150 bp Illumina reads, respectively, by ART [22] and mixed them together to generate a dataset of 67 vertebrate NGS samples.

### Developing a bias adjustment model

For any pair of NGS samples, their alignment-free dissimilairty *d*(*A*_NGS_,*B*_NGS_) is determined by three variables, which are the alignment-free dissimilairty based on their genomes *d*(*A*_*G*_,*B*_*G*_) and the bias caused by each sample Bias(*A*_NGS_) and Bias(*B*_NGS_):

1 $$ d(A_{\text{NGS}}, B_{\text{NGS}}) = F(d(A_{G}, B_{G}), \text{Bias}(A_{\text{NGS}}), \text{Bias}(B_{\text{NGS}}))  $$

We define the bias of an NGS sample A by the following equation:

2 $$ \text{Bias}(A_{\text{NGS}}) = d\left(A_{\text{NGS}}, A_{\text{NGS}}^{R}\right)  $$

where *A*_NGS_ is the original NGS sample and $A_{\text {NGS}}^{R}$ is a mapped NGS sample that each read in it is a reverse complementary mapping of a read in the original NGS sample. For example, the NGS sample in the left of Fig. 1a has reads {AACT, GACG, TTAT, ATAA, CGTC, AGTT}, and its corresponding $A_{\text {NGS}}^{R}$ can be inferred by mapping each read in *A*_NGS_ to its reverse complementary read and thus is {AGTT, CGTC, ATAA, TTAT, GACG, AACT}, which is exactly the same as *A*_NGS_. The NGS sample in the left of Fig. 1b has reads {ACTG, GTTA, ATAA}, and its $A_{\text {NGS}}^{R}$ should be {CAGT, TAAC, TTAT} accordingly, which is apparently different from *A*_NGS_.

Given an dissimilarity measure, such as $d_{2}^{s}$ or $d_{2}^{*}$, the Bias(*A*_NGS_) can then be calculated between *A*_NGS_ and $A_{\text {NGS}}^{R}$. We expect that Bias(*A*_NGS_) will increase as the sequencing depth of *A*_NGS_ decreases or the sequencing error rate increases, as shown in Fig. 1b. The advantage of defining Bias(*A*_NGS_) in this way is that we do not need to estimate sequencing depth or sequencing error rate explicitly, but this information has already been implicitly considered when we compare *A*_NGS_ with $A_{\text {NGS}}^{R}$.

Given *d*(*A*_*G*_,*B*_*G*_),*d*(*A*_NGS_,*B*_NGS_) will increase as Bias(*A*_NGS_) or Bias(*B*_NGS_) increases, as shown in Fig. 2i that samples of high sequencing depth and thus low bias (red points) have higher NGS $s_{2}^{s}$ (lower NGS $d_{2}^{s}$) than samples of low sequencing depth and thus high bias (blue points) even when their genome *s* of interest are the same. In addition, if Bias(*A*_NGS_) and Bias(*B*_NGS_) do not change, *d*(*A*_NGS_,*B*_NGS_) will increase as *d*(*A*_*G*_,*B*_*G*_) increases, as shown in Fig. 2a–h. Since NGS samples in the same subplot have the same number of reads and thus have similar Bias(*A*_NGS_) and Bias(*B*_NGS_), their pairwise *d*(*A*_NGS_,*B*_NGS_) value increases with *d*(*A*_*G*_,*B*_*G*_).

Because of this partial monotonic relationship between *d*(*A*_NGS_,*B*_NGS_) and *d*(*A*_*G*_,*B*_*G*_) given Bias(*A*_NGS_) and Bias(*B*_NGS_), Eq. (1) can be rewritten as:

3 $$  d(A_{G}, B_{G}) = G(d(A_{\text{NGS}}, B_{\text{NGS}}), Bias(A_{\text{NGS}}), \text{Bias}(B_{\text{NGS}}))  $$

where *G* is a general function. Therefore, the bias adjustment process can be characterized as a regression problem that is capable of predicting the real genome dissimilarity *d*(*A*_*G*_,*B*_*G*_) between any pair of NGS samples. To solve this supervised learning problem, we can first train our regression models on datasets of known *d*(*A*_*G*_,*B*_*G*_),*d*(*A*_NGS_,*B*_NGS_),Bias(*A*_NGS_), and Bias(*B*_NGS_). Then, for any new pair of NGS samples, we first calculate their *d*(*A*_NGS_,*B*_NGS_),Bias(*A*_NGS_), and Bias(*B*_NGS_) and use our model to predict its *d*(*A*_*G*_,*B*_*G*_). After bias adjustment, our sequence comparison can be based on the predicted unbiased *d*(*A*_*G*_,*B*_*G*_) instead of biased *d*(*A*_NGS_,*B*_NGS_).

### Model training and evaluation

#### Creating training samples

We trained 2 neural network regression models that are widely used to solve nonlinear regression problems for $d_{2}^{s}$ and $d_{2}^{*}$ separately using the 21 primate dataset. Instead of training on NGS samples we generated previously to plot Fig. 2, we generated a new dataset by simulating 8 NGS samples of different number of reads (1 M, 3 M, 5 M, 7 M, 9 M, 11 M, 13 M, and 15 M) for each genome again and mixed them together. The samples are denoted from $P^{1}_{\text {NGS}}$ to $P^{168}_{\text {NGS}}$, respectively. We describe how we trained the bias adjustment model for $d_{2}^{s}$ in the following section. The same training method was used for $d_{2}^{*}$ and can be easily generalized for other alignment-free methods.

For each pair of NGS samples $P^{i}_{\text {NGS}}$ and $P^{j}_{\text {NGS}}$, we calculated their NGS dissimilarity $\left (d_{2}^{s}\left (P^{i}_{\text {NGS}}, P^{j}_{\text {NGS}}\right)\right)$, their genome dissimilarity $\left (d_{2}^{s}\left (P^{i}_{G}, P^{j}_{G}\right)\right), \text {Bias}\left (P^{i}_{\text {NGS}}\right),$ and $\text {Bias}\left (P^{j}_{\text {NGS}}\right)$ using *k*mer length from 5 to 14 and Markovian order = *k*−2. For each *k*mer length, there are 168×167=28,056 pairs, so that *X*_*k*_ will be a matrix of dimension 28,056×3 and *y*_*k*_ will be a vector of length 28,056 as shown below. To ensure that our model can train $\text {Bias}\left (P^{i}_{\text {NGS}}\right)$ and $\text {Bias}\left (P^{j}_{\text {NGS}}\right)$ symmetrically, both $d_{2}^{s}\left (P^{i}_{\text {NGS}}, P^{j}_{\text {NGS}}\right)$ and $d_{2}^{s}\left (P^{j}_{\text {NGS}}, P^{i}_{\text {NGS}}\right)$ were included in the training samples, which was verified after model training and shown in Additional file 1: Figure S21. In order to build a regression model that is capable of adjusting the bias for different *k*mer lengths, we concatenated *X*_*k*_ from *X*_5_ to *X*_14_ vertically and concatenated *y*_*k*_ from *y*_5_ to *y*_14_. Therefore, our final $X = \left [X_{5}^{T}, X_{6}^{T} \dots X_{14}^{T}\right ]^{T}$ is a 280,560×3 matrix and $y = \left [y_{5}^{T}, y_{6}^{T}, \dots y_{14}^{T}\right ]^{T}$ is a 280,560×1 vector.

$$\begin{aligned} \underbrace{ \left[\begin{array}{ccc} d_{2}^{s}\left(P^{1}_{\text{NGS}}, P^{2}_{\text{NGS}}\right) & \text{Bias}\left(P^{1}_{\text{NGS}}\right) & \text{Bias}\left(P^{2}_{\text{NGS}}\right)\\ d_{2}^{s}\left(P^{1}_{\text{NGS}}, P^{3}_{\text{NGS}}\right) & \text{Bias}\left(P^{1}_{\text{NGS}}\right) & \text{Bias}\left(P^{3}_{\text{NGS}}\right)\\ d_{2}^{s}\left(P^{1}_{\text{NGS}}, P^{4}_{\text{NGS}}\right) & \text{Bias}\left(P^{1}_{\text{NGS}}\right) & \text{Bias}\left(P^{4}_{\text{NGS}}\right)\\ \vdots & \vdots & \vdots \\ d_{2}^{s}\left(P^{1}_{\text{NGS}}, P^{168}_{\text{NGS}}\right) & \text{Bias}\left(P^{1}_{NGS}\right) & \text{Bias}\left(P^{168}_{\text{NGS}}\right)\\ d_{2}^{s}\left(P^{2}_{\text{NGS}}, P^{1}_{\text{NGS}}\right) & \text{Bias}\left(P^{2}_{\text{NGS}}\right) & \text{Bias}\left(P^{1}_{\text{NGS}}\right)\\ d_{2}^{s}\left(P^{2}_{\text{NGS}}, P^{3}_{\text{NGS}}\right) & \text{Bias}\left(P^{2}_{\text{NGS}}\right) & \text{Bias}\left(P^{3}_{\text{NGS}}\right)\\ d_{2}^{s}\left(P^{2}_{\text{NGS}}, P^{4}_{\text{NGS}}\right) & \text{Bias}\left(P^{2}_{\text{NGS}}\right) & \text{Bias}\left(P^{4}_{\text{NGS}}\right) \\ \vdots & \vdots & \vdots \\ d_{2}^{s}\left(P^{168}_{\text{NGS}}, P^{167}_{\text{NGS}}\right) & \text{Bias}\left(P^{168}_{\text{NGS}}\right) & \text{Bias}\left(P^{167}_{\text{NGS}}\right) \end{array}\right] }_{X_{k}} \sim \underbrace{ \left[\begin{array}{c} d_{2}^{s}\left(P^{1}_{G}, P^{2}_{G}\right) \\ d_{2}^{s}\left(P^{1}_{G}, P^{3}_{G}\right) \\ d_{2}^{s}\left(P^{1}_{G}, P^{4}_{G}\right) \\ \vdots \\ d_{2}^{s}\left(P^{1}_{G}, P^{168}_{G}\right) \\ d_{2}^{s}\left(P^{2}_{G}, P^{1}_{G}\right) \\ d_{2}^{s}\left(P^{2}_{G}, P^{3}_{G}\right) \\ d_{2}^{s}\left(P^{2}_{G}, P^{4}_{G}\right) \\ \vdots \\ d_{2}^{s}\left(P^{168}_{G}, P^{167}_{G}\right) \end{array}\right] }_{y_{k}} \end{aligned} $$

#### Training samples augmentation

A data augmentation technique based on prior knowledge was used to further increase the training samples. If there is no bias in NGS samples, then the alignment-free dissimilarity based on NGS samples should be equal to the dissimilarity based on their genomes (*d*(*A*_NGS_,*B*_NGS_)=*d*(*A*_*G*_,*B*_*G*_) ⇔ Bias(*A*_NGS_)=Bias(*B*_NGS_)=0). Therefore, we defined a hyperparameter augmentation ratio as *r*, and randomly simulated *d*_1_ to *d*_*m*_ (*d*_*i*_∼*U*(0,1)) and concatenated *X*_*A*_ and *y*_*A*_ shown as below to our training samples *X* and *y*, respectively, to fit our model. The sizes of *X*_*A*_ and *y*_*A*_ were determined by the size of training samples and augmentation ratio *r* where *m*=|*X*|×*r*.

$$ \underbrace{ \left[\begin{array}{ccc} d_{1} & 0 & 0\\ d_{2} & 0& 0\\ d_{3} & 0& 0\\ \vdots & \vdots & \vdots \\ d_{m} & 0 & 0 \end{array}\right] }_{X_{A}} \sim \underbrace{ \left[\begin{array}{c} d_{1} \\ d_{2} \\ d_{3} \\ \vdots \\ d_{m} \end{array}\right] }_{y_{A}} $$

#### Hyperparameter tuning and evaluation

**Fig. 7:**
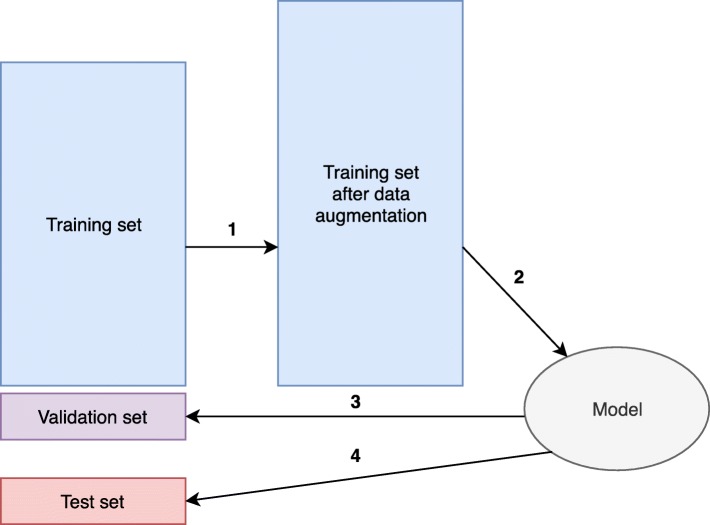
Diagram of hyperparameter tuning and evaluation. (1) Trainig set is augmented. (2) Training set after data augmentation is used to fit the model. (3) The trained model is used to predict *d*(*A*_*G*_,*B*_*G*_) for the validation set. For each combination of hyperparameters, we repeated steps 1–3 ten times to calculate the average *R*^2^, and the combination of hyperparameters with the highest average *R*^2^ was chosen. (4) After hyperparameter tuning, the final model is tested on the test set

A neural network regression model with ReLU activation (sklearn.neural_network.MLPRegressor), was trained and a grid search algorithm was implemented to find the optimal combination of hyperparameters such as hidden layer sizes, regularization term, and augmentation ratio. The workflow is described below and also shown in Fig. 7.

1. Twenty-eight thousand fifty-six (10%) samples were randomly selected as a held-out test set. The remaining 252,504 (90%) samples were used as a training set.
2. A given combination of hyperparameters was chosen. Steps 3–4 were repeated 10 times, and the average *R*^2^ under this combination of hyperparameters were calculated (10-fold cross-validation).
3. Ten percent of the samples from the training set were randomly chosen as a validation set, the other 90% of the samples were first augmented as aforementioned and then used to fit our model.
4. The trained model was used to predict *d*(*A*_*G*_,*B*_*G*_) for the validation set, and *R*^2^ was calculated.
5. Repeat steps 2–4 with different hyperparameters, and the optimal combination of hyperparameters with the highest average *R*^2^ was chosen (hyperparameter tuning).
6. The optimal combination of hyperparameters was chosen, and trained on all training set, and the final model was used to predict *d*(*A*_*G*_,*B*_*G*_) for the held-out test set and *R*^2^ was calculated (evaluation).

Finally, the combination of hyperparameters with the highest cross-validation score was chosen (1 hidden layer with 2000 neurons, regularization term 0.0001, and augmentation ratio 2) and tested on the held-out test data with an *R*^2^ value 0.98 for $d_{2}^{s}$ and 0.99 for $d_{2}^{*}$. The final models for $d_{2}^{s}$ and $d_{2}^{*}$ were then used to adjust the bias for primate, mammalian, vertebrate, and white oak NGS datasets. It should be mentioned that altough *d*(*A*_*G*_,*B*_*G*_) and *d*(*B*_*G*_,*A*_*G*_) were almost identical as shown in Additional file 1: Figure S21, we take the average of *d*(*A*_*G*_,*B*_*G*_) and *d*(*B*_*G*_,*A*_*G*_) as the final predicted dissimilarity between A and B to strictly satisfy the symmetry property.

### White oak continental origin prediction by *k*-NN and $d_{2}^{*}$

For each sequencing quantity (50, 100, and 300 Mbp), we first calculated the pairwise $d_{2}^{*}$ using *K*=12 and *M*=10 between each pair of samples in the dataset. Then, 92 samples were randomly divided into the reference set and query set. The number of samples in the reference set ranges from 91 (leave-one-out), 77, 60, 45, and 30 to 15. For each sample in the query set, we found its *k*-nearest (*k*=1–10) neighbors measured by $d_{2}^{*}$ in the reference set and predicted its continental origin by a majority vote. For each reference size, we split 100 times and the prediction accuracy was averaged over 100 splits and shown in Additional file 1: Table S1. We then randomly selected 30 samples from the 50 Mbp dataset, 31 samples from the 100 Mbp dataset, and the 300 Mbp dataset as a mixed dataset. The same prediction method was used, and accuracies with and without bias adjustment were shown in Table 1.

## Supplementary information

**Additional file 1** Supplementary methods, supplementary figures, supplementary tables.

**Additional file 2** Review history.
