# Supplementary material for: Afann: bias adjustment for alignment-free sequence comparison based on sequencing data using neural network regression
Source: Genome Biol. 2019 Dec 4;20:266. doi: 10.1186/s13059-019-1872-3 (PMC6891986; doi:10.1186/s13059-019-1872-3)
Supplement: Supplementary file 1 — Additional file 1 Supplementary methods, supplementary figures, supplementary tables. [file 13059_2019_1872_MOESM1_ESM.pdf]

Afann: bias adjustment for alignment-free sequence comparison based on sequencing data using neural network regression: Supplementary Material

Kujin Tang[1], Jie Ren[1], and Fengzhu Sun *[1]

[1]Quantitative and Computational Biology, Department of Biological Sciences, University of Southern California, CA, USA

Appendix A: Aligment-free distance/dissimilarity measures

Given two genomic sequences or NGS samples i and j and a given word length k, we first count the number of occurrences of all kmers in sequence i and sequence j, respectively. The full set of kmers of length k is defined as $\mathcal{A}^k$ where $\mathcal{A} = (A, T, C, G)$ for nucleotide sequences. For a given kmer w, its occurrences in i is defined as $N_w^{(i)}$ and the frequency or the relative abundance of this kmer is defined as $f_w^{(i)} = \frac{N_w^{(i)}}{\sum_w N_w^{(i)}}$.

Some dissimilarity measures such as d_2^* and d_2^s need an m-th order Markov model for the background sequence. The expected number of occurrences of word w, $\mathbb{E}N_w^{(i)}$, can be calculated from the stationary probability of the first m-mer $w[1:m]$ and the transition probabilities from the n-th m-mer $w[n:n+m-1]$ to the $(n+m)$-th nucleotide $w[n+m]$:

$$\mathbb{E}N_w^{(i)} = (L^{(i)} - k + 1)\mu(w[1:m]) \prod_{n=1}^{k-m} \pi(w[n:n+m-1], w[n+m])$$

where $L^{(i)}$ is the length of sequence i, μ is the stationary probability and π is the transition probability that can be estimated from the sequence data. The difference between the number of occurrences of kmer w and its expected occurrences is defined as $\tilde{N}_w^{(i)} = N_w^{(i)} - \mathbb{E}N_w^{(i)}$.

*To whom correspondence should be addressed. Tel: +1 (213)-740-2413; Fax: +1 (213)-740-8631; Email: fsun@usc.edu

Manhattan

The Manhattan distance (Ma) is defined as:

$$Ma = \sum_{w \in \mathcal{A}^k} |f_w^{(i)} - f_w^{(j)}|$$

Euclidean

The Euclidean distance (Eu) is defined as:

$$Eu = \sqrt{\sum_{w \in \mathcal{A}^k} |f_w^{(i)} - f_w^{(j)}|^2}$$

CVTree [**1**]

The *CVTree* dissimilarity is defined as:

$$CVTree = \frac{1}{2}\left(1 - \frac{\sum_{w \in \mathcal{A}^k} \hat{f}_w^{(i)} \hat{f}_w^{(j)}}{\sqrt{\sum_{w \in \mathcal{A}^k} (\hat{f}_w^{(i)})^2}\sqrt{\sum_{w \in \mathcal{A}^k} (\hat{f}_w^{(j)})^2}}\right)$$

where $\hat{f}_w^{(i)} = \frac{\tilde{N}_w^{(i)}}{\mathbb{E}N_w^{(i)}}$. *CVTree* calculates $\mathbb{E}N_w^{(i)}$ by assuming a $(k-2)$-th order Markov chain for genomic sequences.

d_2^* [**2**]

The d_2^* dissimilarity is defined as:

$$d_2^* = \frac{1}{2}\left(1 - \frac{\sum_{w \in \mathcal{A}^k} \bar{f}_w^{(i)} \bar{f}_w^{(j)}}{\sqrt{\sum_{w \in \mathcal{A}^k} (\bar{f}_w^{(i)})^2}\sqrt{\sum_{w \in \mathcal{A}^k} (\bar{f}_w^{(j)})^2}}\right)$$

where $\bar{f}_w^{(i)} = \frac{\tilde{N}_w^{(i)}}{\sqrt{\mathbb{E}N_w^{(i)}}}$.

d_2^s [2]

The d_2^s dissimilarity is defined as:

$$d_2^s = \frac{1}{2}\left(1 - \frac{\sum_{w\in\mathcal{A}^k} \tilde{f}_w^{(i)} \tilde{f}_w^{(j)}}{\sqrt{\sum_{w\in\mathcal{A}^k} (\tilde{f}_w^{(i)})^2}\sqrt{\sum_{w\in\mathcal{A}^k} (\tilde{f}_w^{(j)})^2}}\right)$$

where $\tilde{f}_w^{(i)} = \frac{\tilde{N}_w^{(i)}}{((\tilde{N}_w^{(i)})^2+(\tilde{N}_w^{(j)})^2)^{\frac{1}{4}}}$ and $\tilde{f}_w^{(j)} = \frac{\tilde{N}_w^{(j)}}{((\tilde{N}_w^{(i)})^2+(\tilde{N}_w^{(j)})^2)^{\frac{1}{4}}}$.

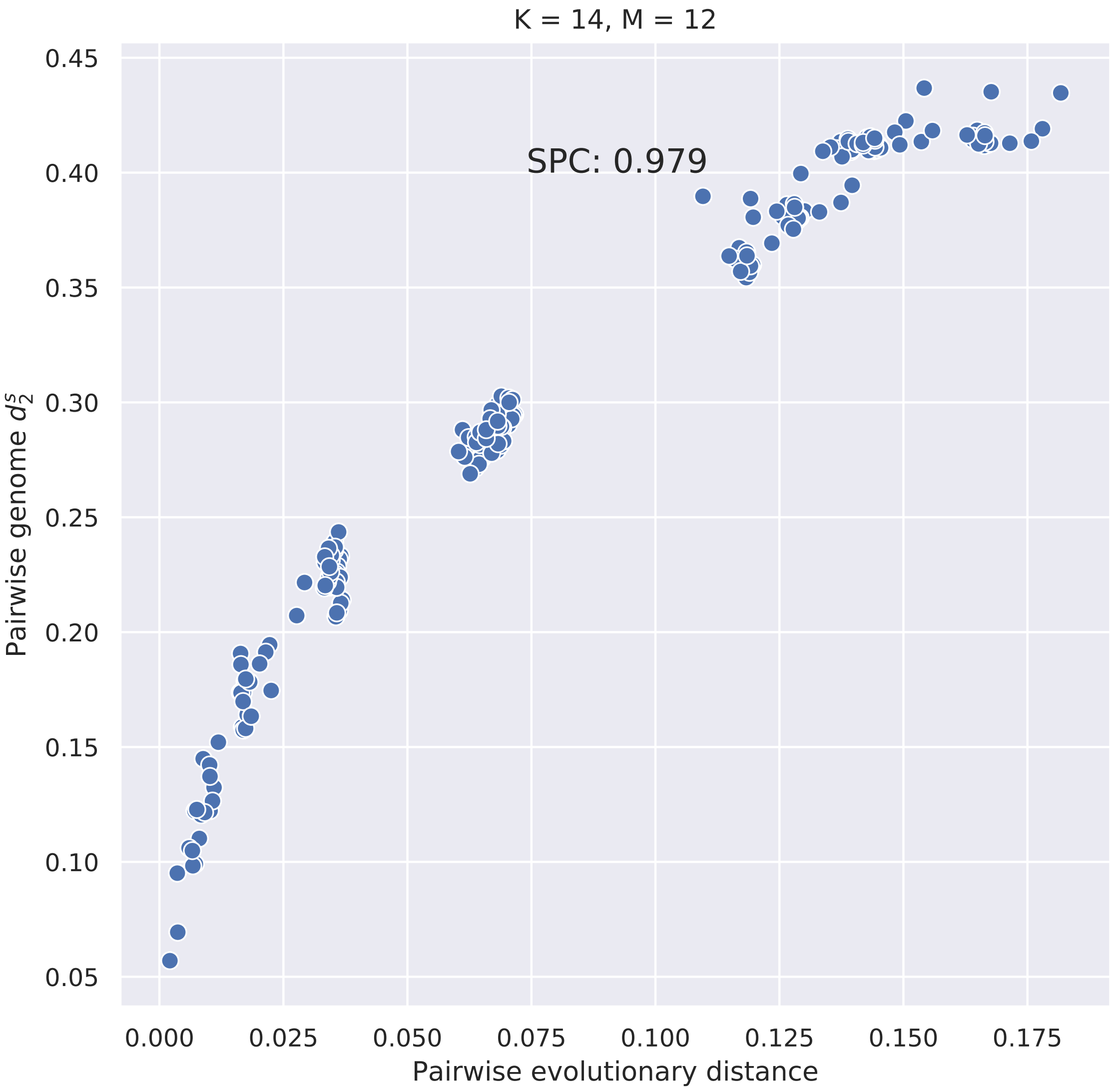

Figure S1: Relationship between pairwise d_2^s using $K = 14$ and evolutionary distances among 21 primates. X-axis is the pairwise primate evolutionary distances estimated by alignment-based method in [3] and Y-axis is the pairwise d_2^s caculated based on primate genomes using $K = 14$ and $M = 12$. The Spearman correlation coefficient (SPC) is 0.979.

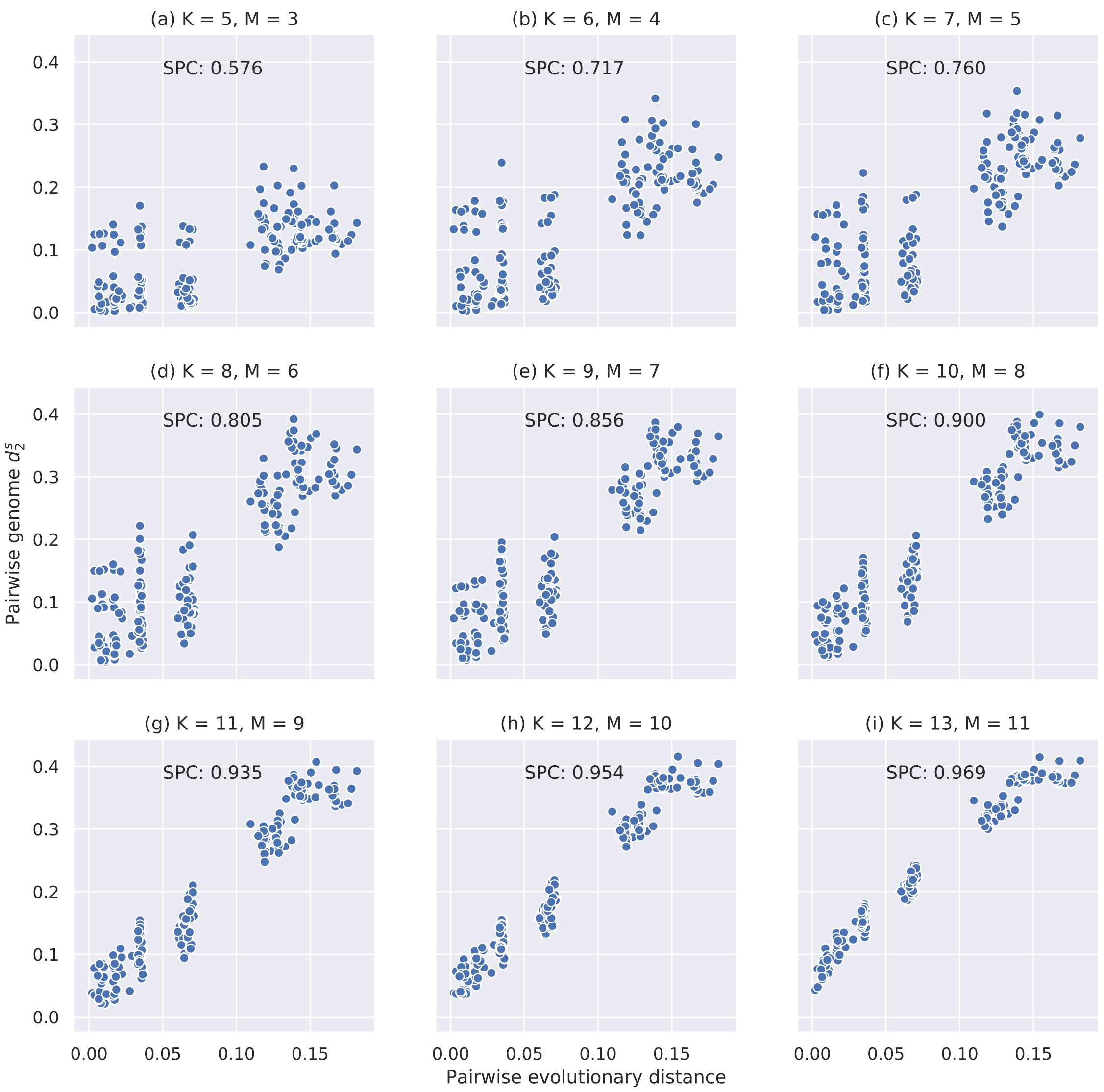

Figure S2: Relationship between pairwise d_2^s using $K = 5$ to $K = 13$ and evolutionary distances among 21 primates. X-axis is the pairwise primate evolutionary distances estimated by alignment-based method in [3] and Y-axis is the pairwise d_2^s caculated based on primate genomes using $K = 5$ to $M = 13$ and $M = K - 2$. The corresponding Spearman correlation coefficients (SPC) for each K are shown on the subplot.

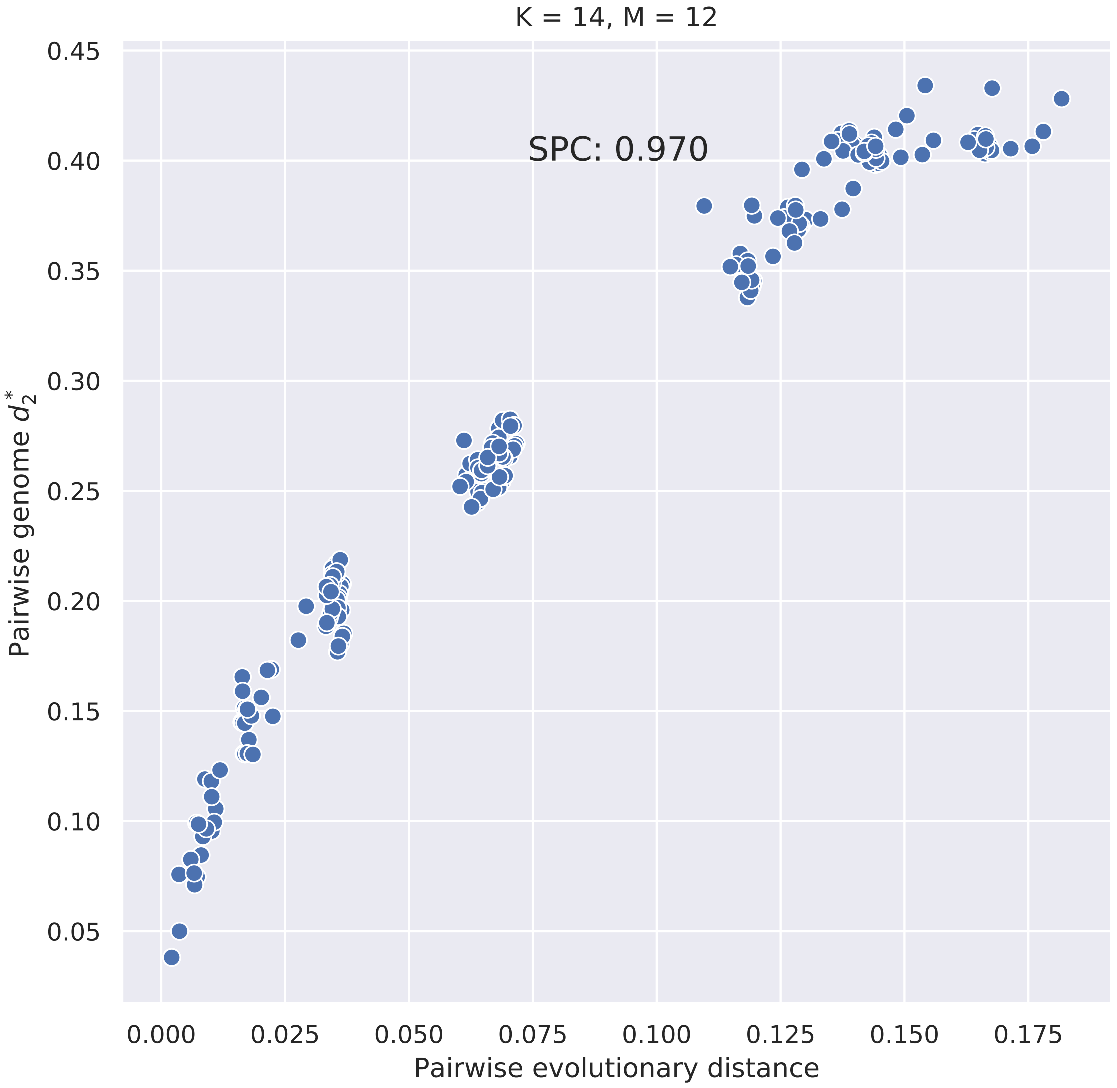

Figure S3: Relationship between pairwise d_2^* using $K = 14$ and evolutionary distances among 21 primates. X-axis is the pairwise primate evolutionary distances estimated by alignment-based method in [3] and Y-axis is the pairwise d_2^* caculated based on primate genomes using $K = 14$ and $M = 12$. The Spearman correlation coefficient (SPC) is 0.970.

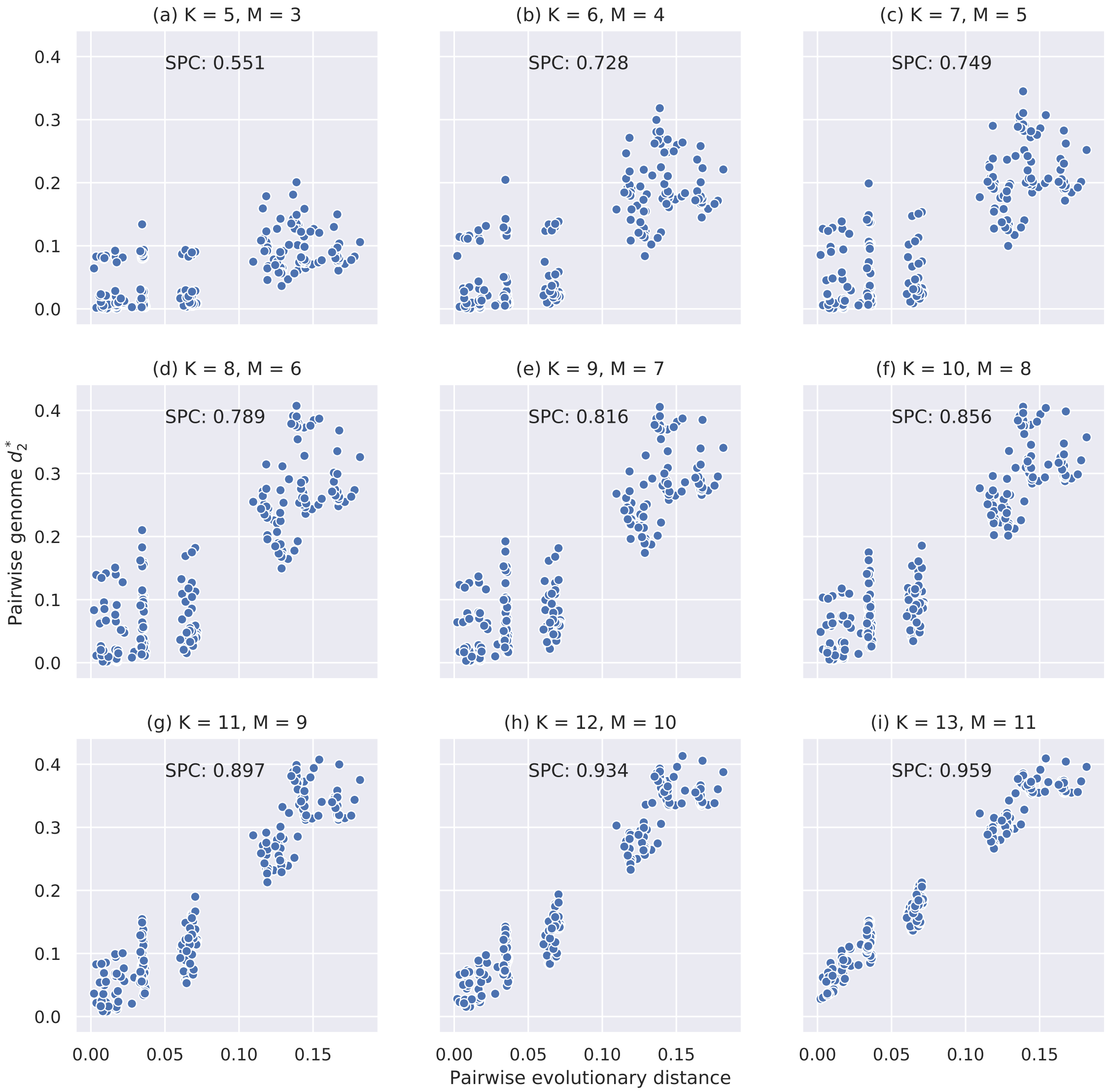

Figure S4: Relationship between pairwise d_2^* using $K = 5$ to $K = 13$ and evolutionary distances among 21 primates. X-axis is the pairwise primate evolutionary distances estimated by alignment-based method in [3] and Y-axis is the pairwise d_2^* caculated based on primate genomes using $K = 5$ to $M = 13$ and $M = K - 2$. The corresponding Spearman correlation coefficients (SPC) for each K are shown on the subplot.

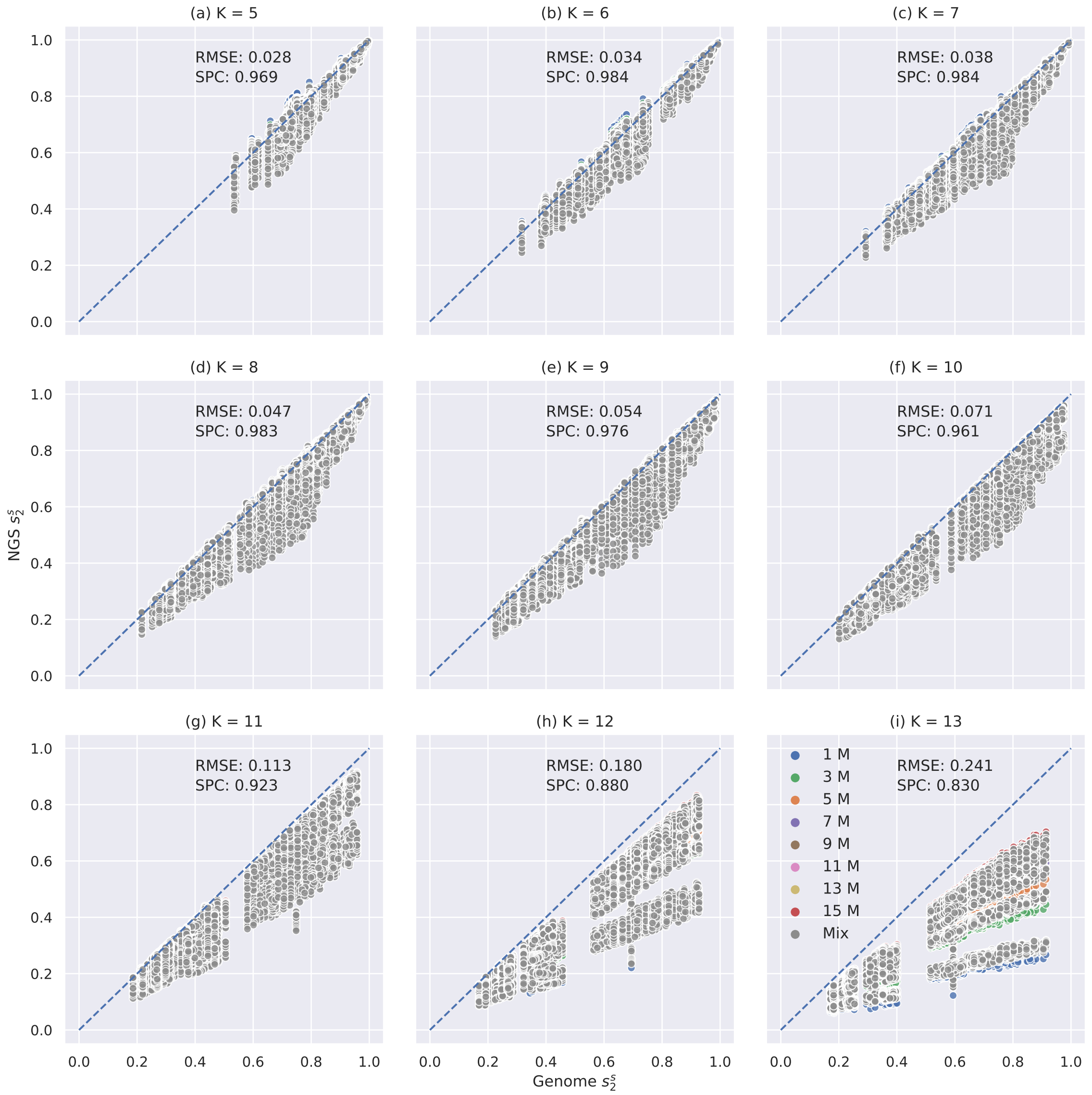

Figure S5: Relationship between pairwise s_2^s estimated by primate genomes using $K = 5$ to $K = 13$, $M = K - 2$ and NGS samples of different numbers of reads without bias adjustment. X-axis is the pairwise s_2^s estimated by genomes and Y-axis is the pairwise s_2^s estimated based on mixed NGS samples. (a)-(h) show relationship between s_2^s estimated based on mixed NGS using different K and M and s_2^s estimated based on primate genomes. NGS samples of different numbers of reads are colored accordingly. 'Mix' means two NGS samples have different numbers of reads (e.g between 1 M and 5 M or between 7 M and 11 M) and is colored in grey. The root mean squared error (RMSE) and Spearman correlation coefficients (SPC) between pairwise s_2^s estimated based on NGS samples and genomes are shown on each subplot.

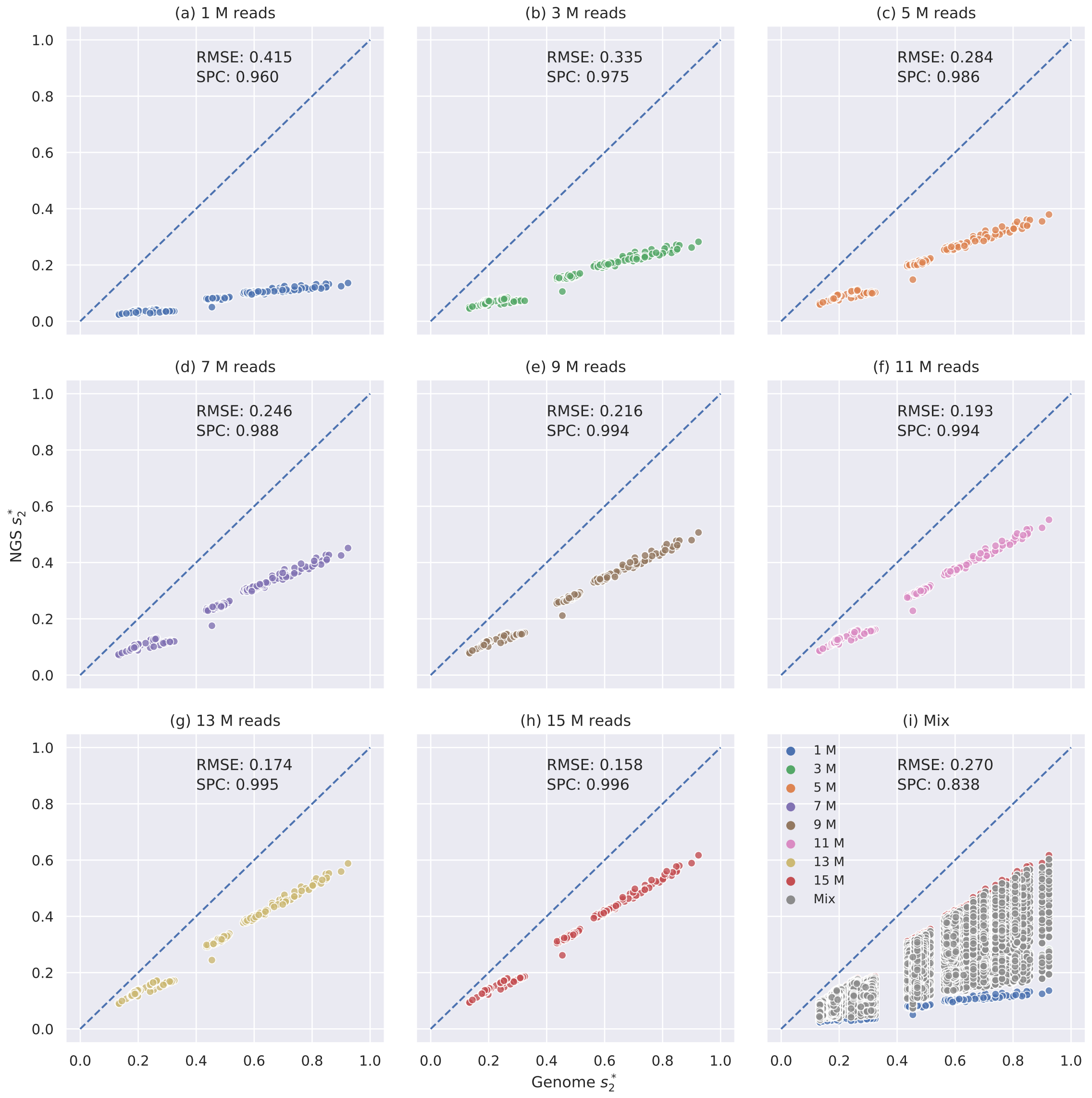

Figure S6: Relationship between pairwise s_2^* estimated by primate genomes and NGS samples using $K = 14$ and $M = 12$ of different numbers of reads without bias adjustment. X-axis is the pairwise s_2^* estimated by genomes and Y-axis is the pairwise s_2^* estimated based on NGS samples. (a)-(h) show the relationship between s_2^s estimated based on primate genomes and s_2^* estimated based on NGS samples of only 1 M, 3M, 5 M, 7M, 9 M, 11 M, 13 M or 15 M reads, respectively. (i) shows pairwise s_2^* estimated based on mixed NGS samples. NGS samples of different numbers of reads are colored accordingly. 'Mix' means two NGS samples have different numbers of reads (e.g between 1 M and 5 M or between 7 M and 11 M) and is colored in grey. The root mean squared error (RMSE) and Spearman correlation coefficients (SPC) between pairwise s_2^* estimated based on NGS samples and genomes are shown on each subplot.

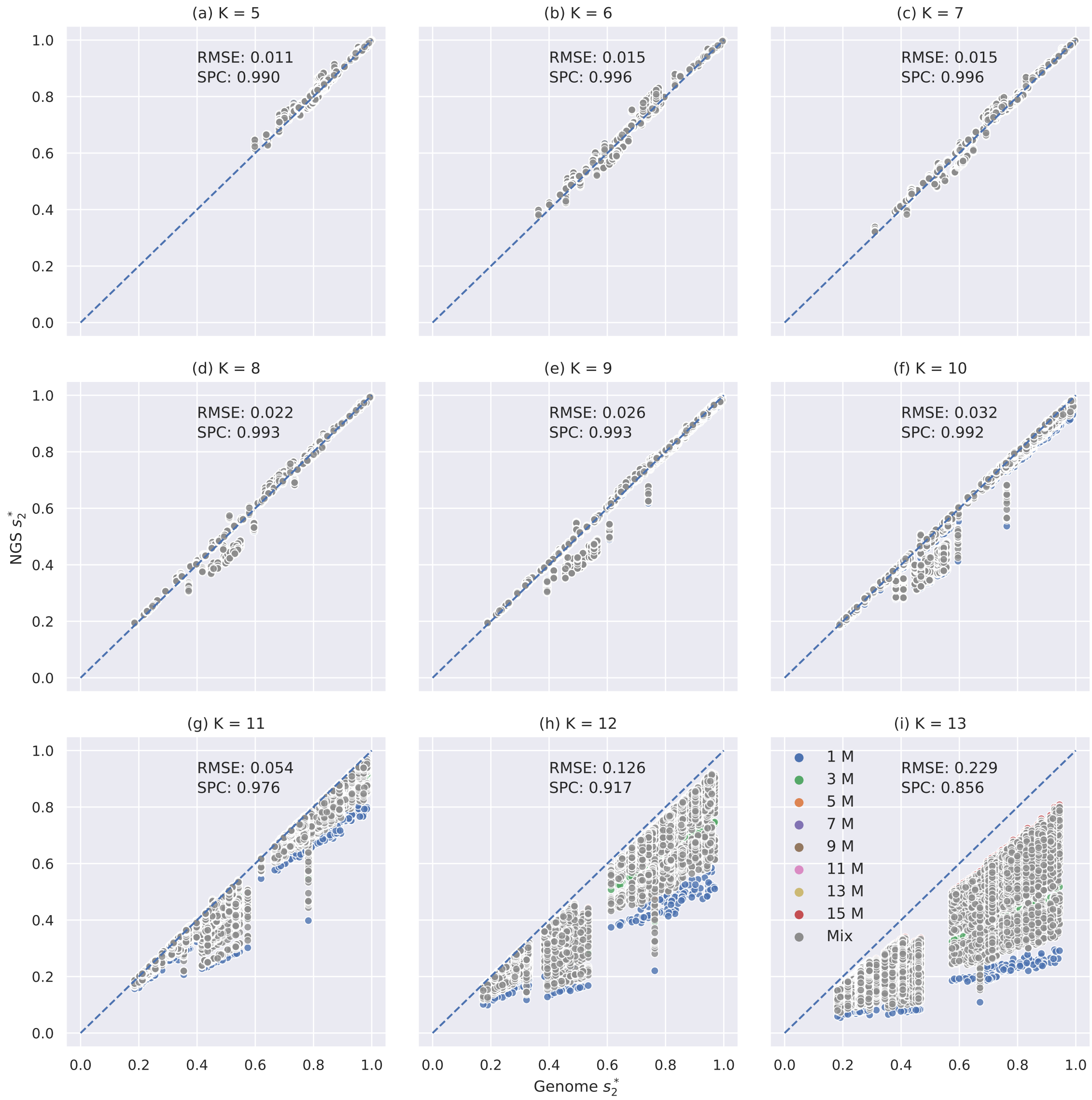

Figure S7: Relationship between pairwise s_2^* estimated by primate genomes using $K = 5$ to $K = 13$, $M = K - 2$ and NGS samples of different numbers of reads without bias adjustment. X-axis is the pairwise s_2^* estimated by genomes and Y-axis is the pairwise s_2^* estimated based on mixed NGS samples. (a)-(h) show relationship between s_2^* estimated based on mixed NGS using different K and M and s_2^* estimated based on primate genomes. NGS samples of different numbers of reads are colored accordingly. 'Mix' means two NGS samples have different numbers of reads (e.g between 1 M and 5 M or between 7 M and 11 M) and is colored in grey. The root mean squared error (RMSE) and Spearman correlation coefficients (SPC) between pairwise s_2^* estimated based on NGS samples and genomes are shown on each subplot.

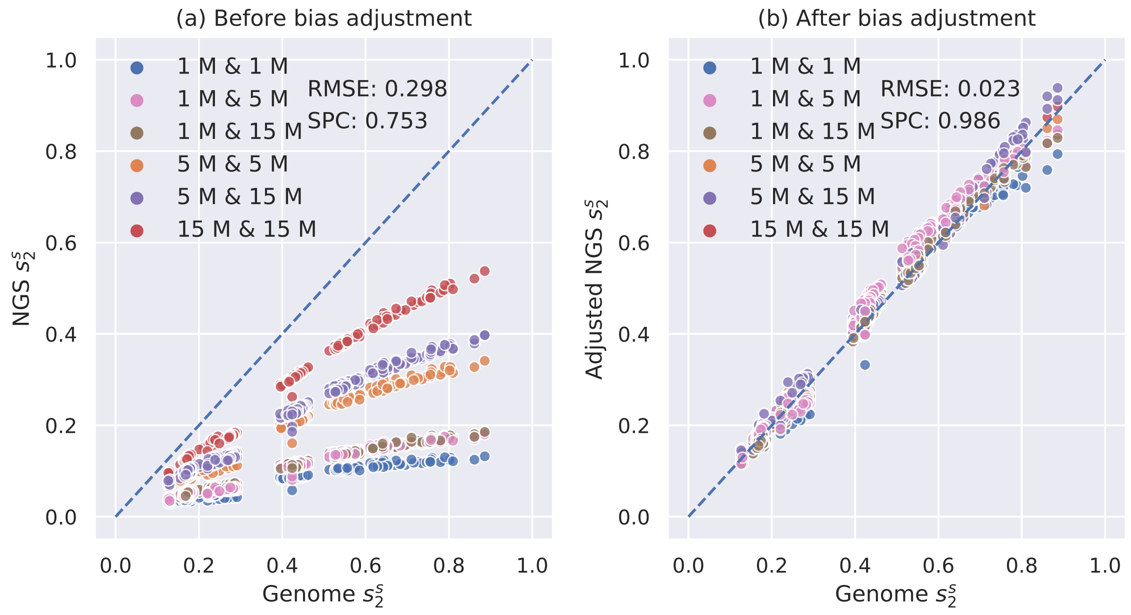

Figure S8: Relationship between pairwise s_2^s estimated using $K = 14$ and $M = 12$ based on 21 primate genomes and NGS samples of 1 M, 5 M and 15 M reads (samples of other sequencing depths were not shown in this figure for less crowded visualization). (a) relationship before bias adjustment. (b) relationship after bias adjustment for NGS s_2^s. '1 M & 1 M' represents the s_2^s between two NGS samples of 1 M reads and '1 M & 15 M' represents the s_2^s bewteen one NGS sample of 1 M reads and the other NGS sample of 15 M reads. The root mean squared error (RMSE) was decreased and the Spearman correlation coeffient (SPC) between pairwise genome s_2^s and NGS s_2^s was increased after bias adjustment.

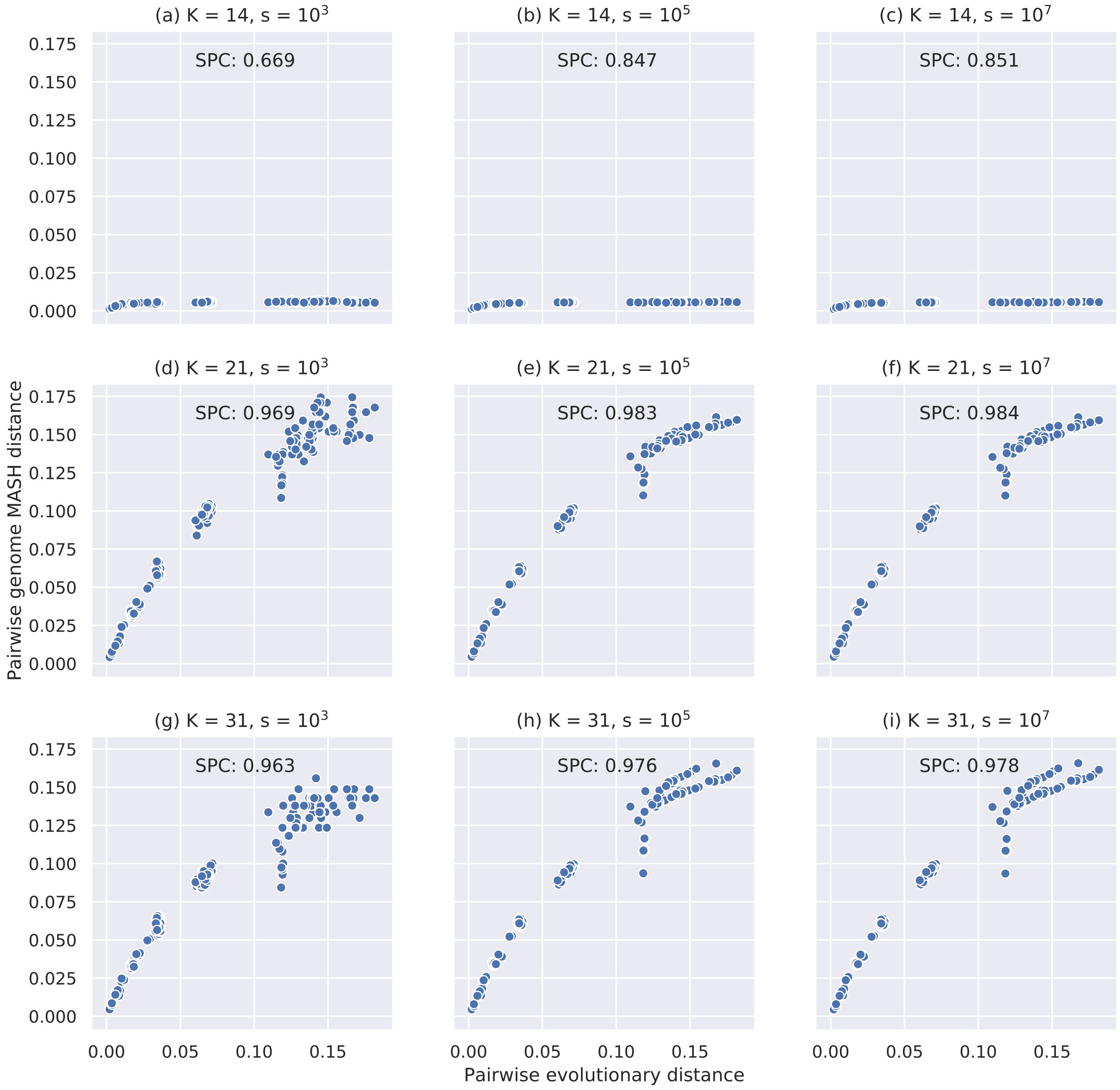

Figure S9: Relationship between pairwise Mash distances using $K = 14$, $K = 21$, $K = 31$ and sketch size $s = 10^3$, $s = 10^5$, $s = 10^7$ and evolutionary distances among 21 primates. X-axis is the pairwise primate evolutionary distances estimated by alignment-based method in [3] and Y-axis is the pairwise Mash distances calculated based on primate genomes. The corresponding Spearman correlation coefficients (SPC) for each combination of K and s are shown on the subplot. Mash distances with $K = 21$ and $s = 10^7$ and the evolutionary distances have the highest Spearman correlation coefficient 0.984.

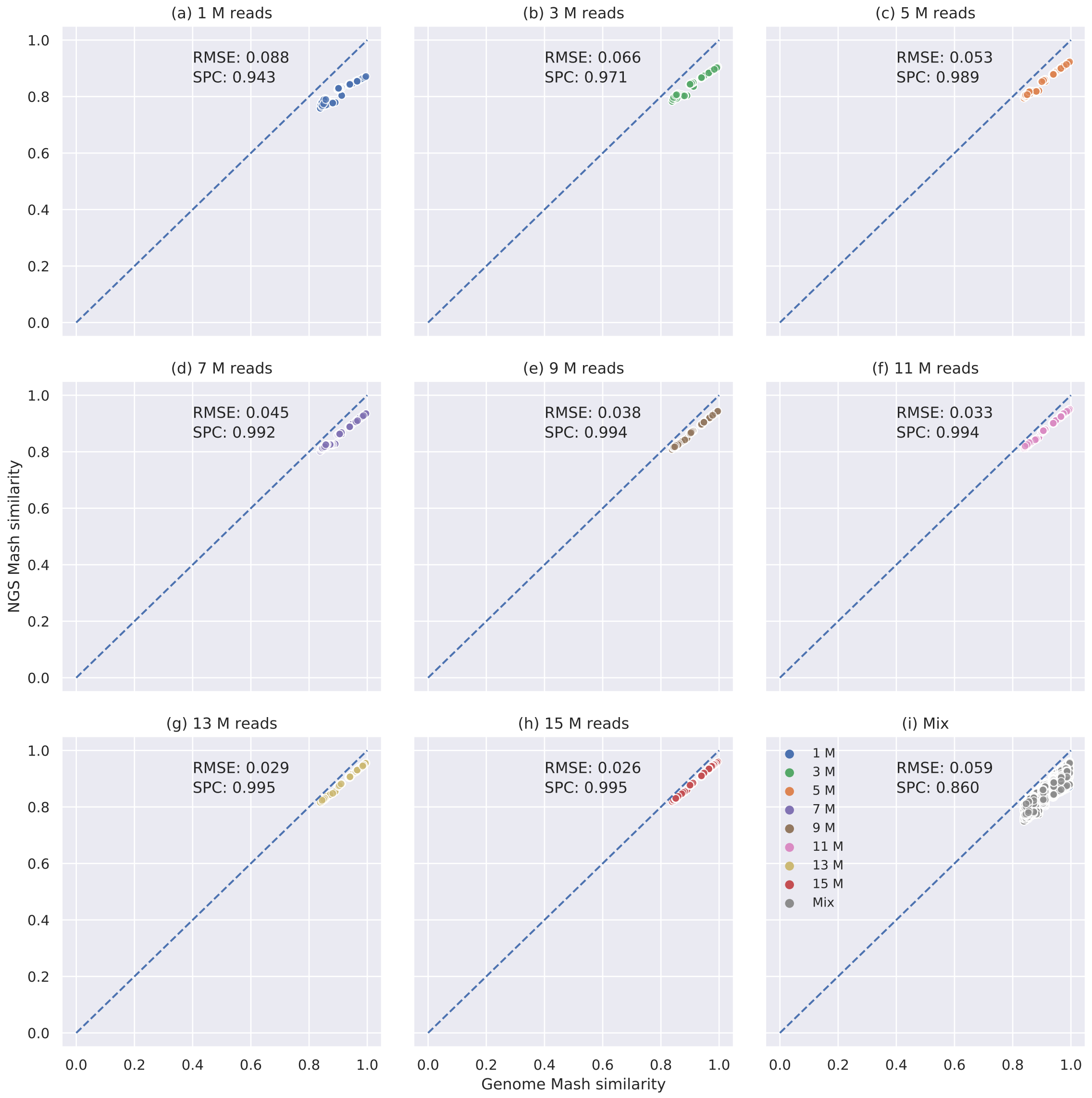

Figure S10: Relationship between pairwise Mash similarity estimated by primate genomes using $K = 21$ and sketch size $s = 10^7$ and NGS samples of different numbers of reads. X-axis is the pairwise Mash similarity estimated by genomes and Y-axis is the pairwise Mash similarity estimated based on NGS samples. (a)-(h) show the relationship between Mash similarity estimated based on primate genomes and Mash similarity estimated based on NGS samples of only 1 M, 3M, 5 M, 7M, 9 M, 11 M, 13 M or 15 M reads, respectively. (i) shows pairwise Mash similarity estimated based on mixed NGS samples. NGS samples of different numbers of reads are colored accordingly. 'Mix' means two NGS samples have different numbers of reads (e.g between 1 M and 5 M or between 7 M and 11 M) and is colored in grey. The root mean squared error (RMSE) and Spearman correlation coefficients (SPC) between pairwise Mash similarity estimated based on NGS samples and genomes are shown on each subplot.

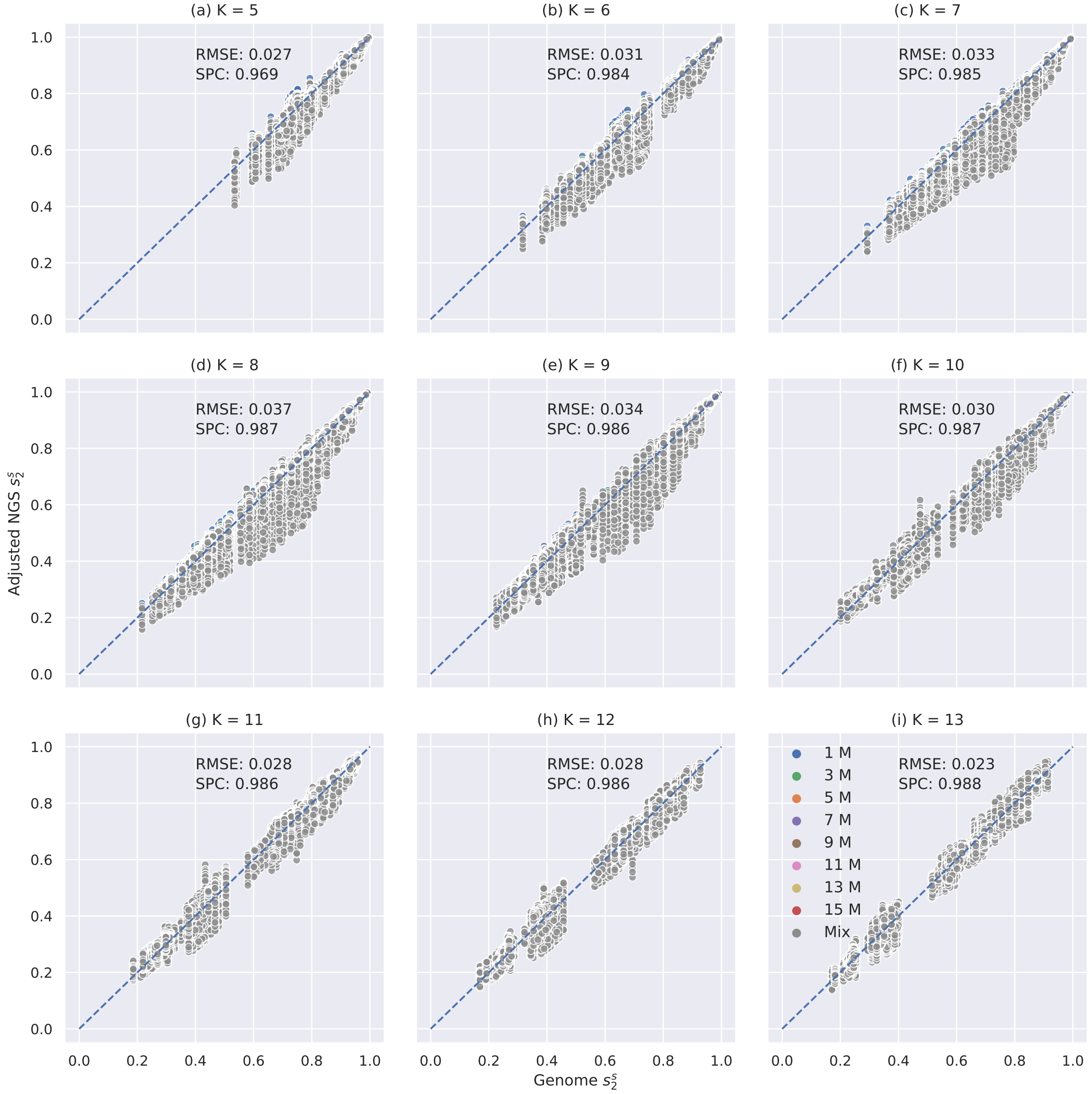

Figure S11: Relationship between pairwise s_2^s estimated by primate genomes using $K = 5$ to $K = 13$, $M = K - 2$ and NGS samples of different numbers of reads with bias adjustment. X-axis is the pairwise s_2^s estimated by genomes and Y-axis is the pairwise s_2^s estimated based on mixed NGS samples after bias adjustment. (a)-(h) show relationship between s_2^s estimated by primate genomes and adjusted s_2^s based on mixed NGS using different K and M. NGS samples of different numbers of reads are colored accordingly. 'Mix' means two NGS samples have different numbers of reads (e.g between 1 M and 5 M or between 7 M and 11 M) and is colored in grey. The root mean squared error (RMSE) and Spearman correlation coefficients (SPC) between pairwise adjusted s_2^s based on NGS samples and genomes are shown on each subplot.

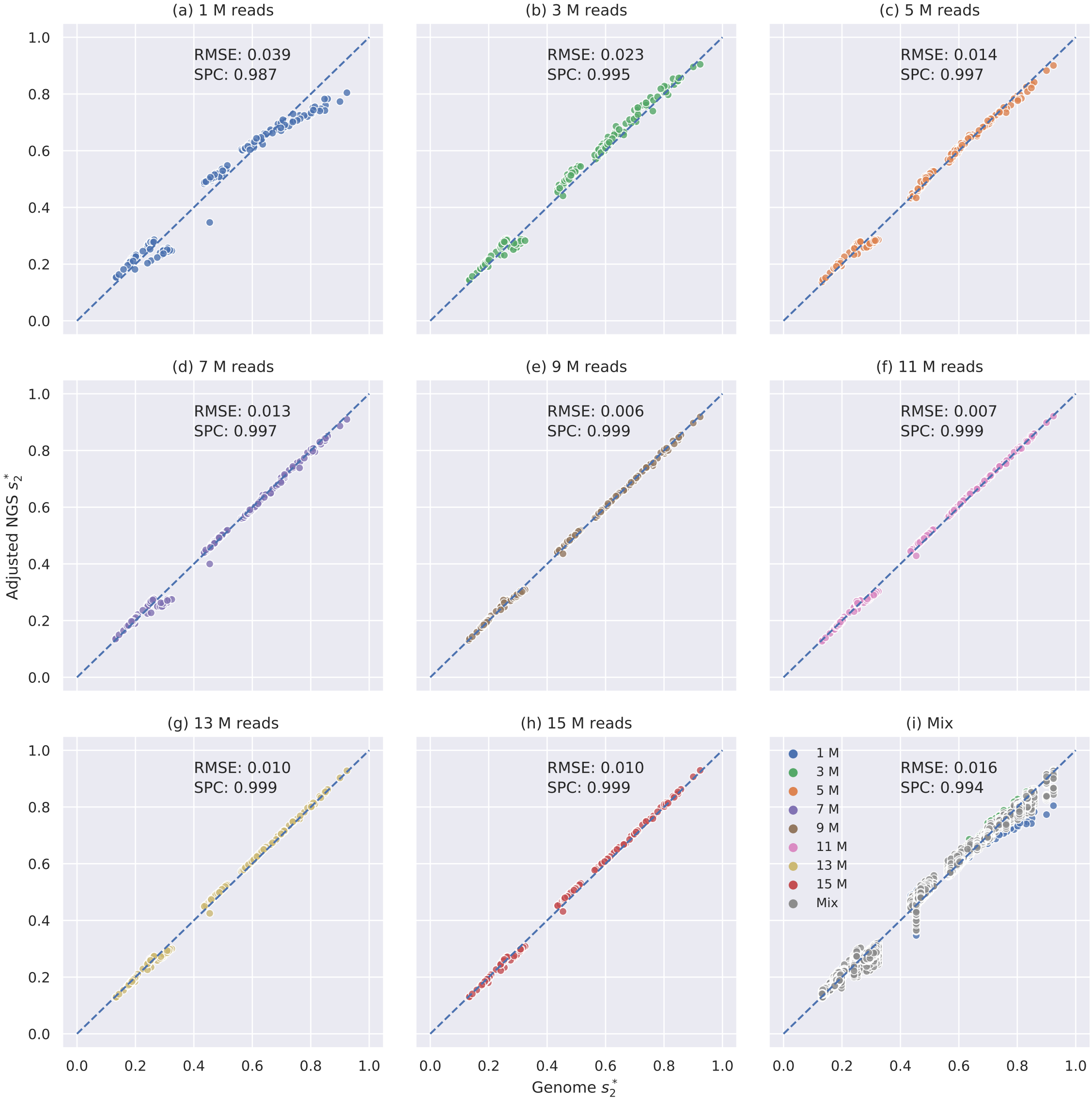

Figure S12: Relationship between pairwise s_2^* estimated by primate genomes and NGS samples using $K = 14$ and $M = 12$ of different numbers of reads with bias adjustment. X-axis is the pairwise s_2^* estimated by genomes and Y-axis is the pairwise s_2^* estimated based on NGS samples after bias adjustment. (a)-(h) show the relationship between s_2^* estimated by primate genomes and adjusted s_2^* based on NGS samples of only 1 M, 3M, 5 M, 7M, 9 M, 11 M, 13 M or 15 M reads, respectively. (i) shows pairwise adjusted s_2^* based on mixed NGS samples. NGS samples of different numbers of reads are colored accordingly. 'Mix' means two NGS samples have different numbers of reads (e.g between 1 M and 5 M or between 7 M and 11 M) and is colored in grey. The root mean squared error (RMSE) and Spearman correlation coefficients (SPC) between pairwise s_2^* estimated based on NGS samples and genomes are shown on each subplot.

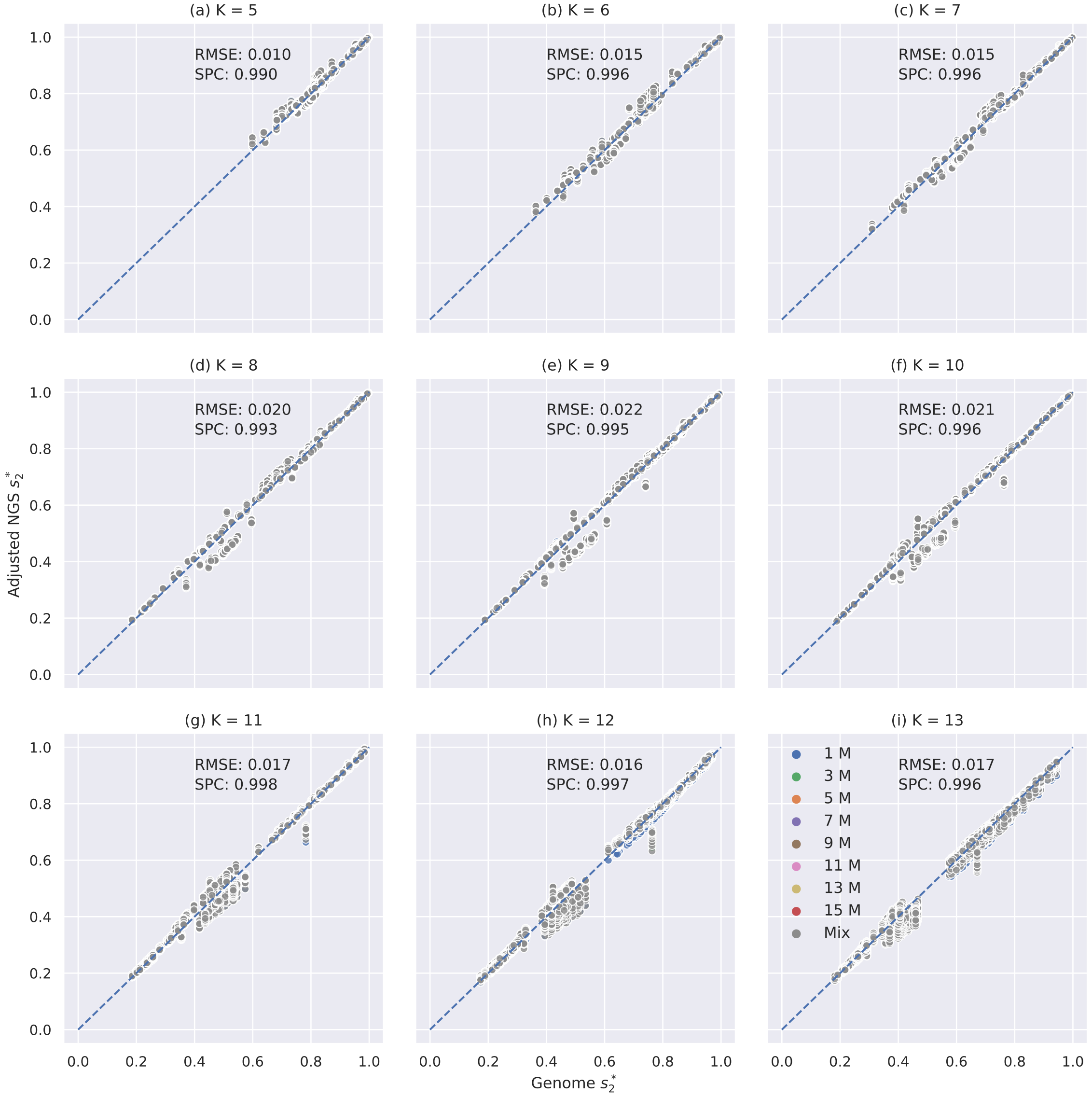

Figure S13: Relationship between pairwise s_2^* estimated by primate genomes using $K = 5$ to $K = 13$, $M = K - 2$ and NGS samples of different numbers of reads with bias adjustment. X-axis is the pairwise s_2^* estimated by genomes and Y-axis is the pairwise s_2^* estimated based on mixed NGS samples after bias adjustment. (a)-(h) show the relationship between adjusted s_2^* based on mixed NGS using different K and M and s_2^* estimated by primate genomes. NGS samples of different numbers of reads are colored accordingly. 'Mix' means two NGS samples have different numbers of reads (e.g between 1 M and 5 M or between 7 M and 11 M) and is colored in grey. The root mean squared error (RMSE) and Spearman correlation coefficients (SPC) between pairwise adjusted s_2^* based on NGS samples and genomes are shown on each subplot.

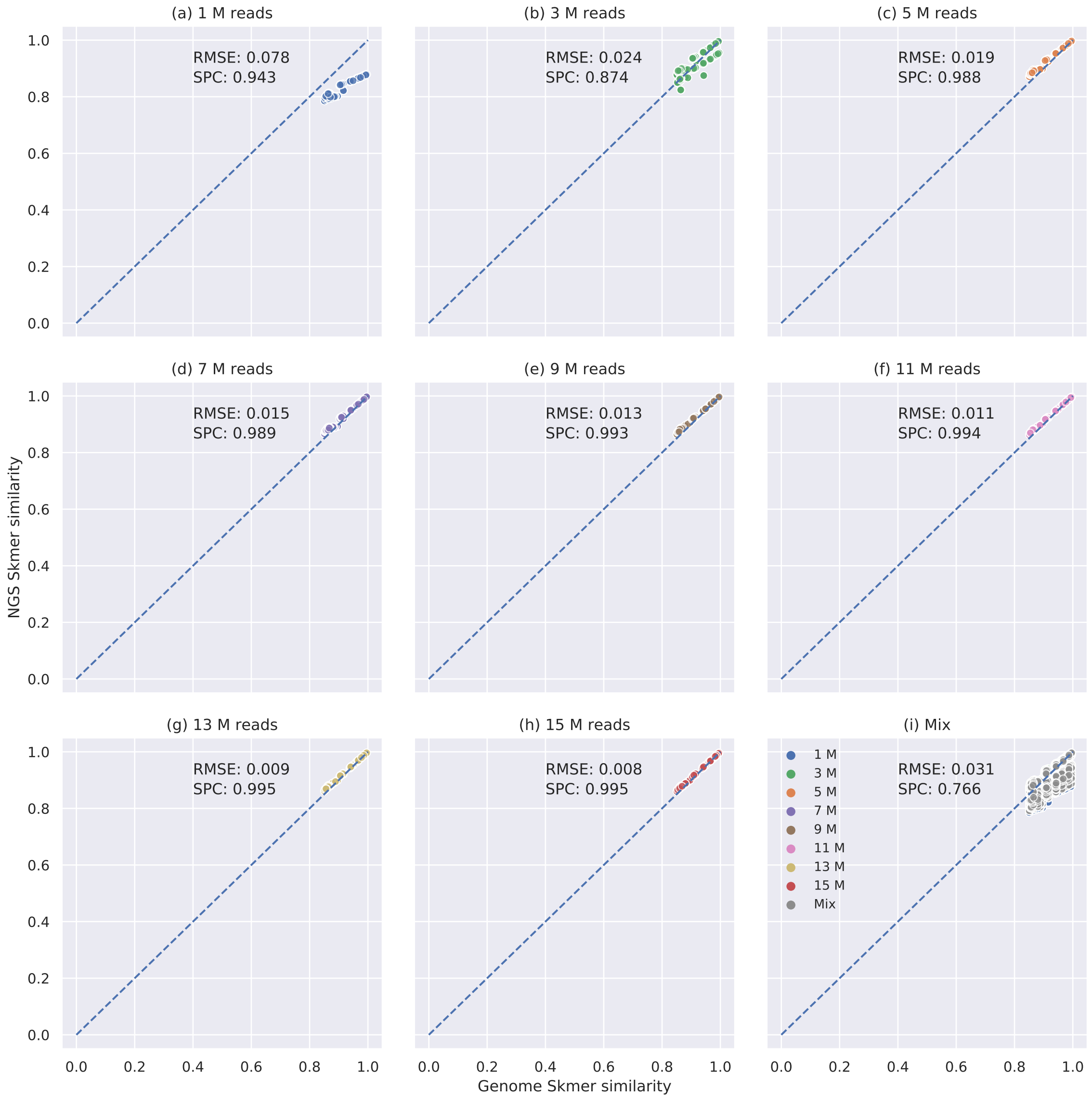

Figure S14: Relationship between pairwise Skmer similarity estimated by primate genomes using $K = 21$ and sketch size $s = 10^7$ and NGS samples of different numbers of reads. X-axis is the pairwise Skmer similarity estimated by genomes and Y-axis is the pairwise Skmer similarity estimated based on NGS samples. (a)-(h) show the relationship between Skmer similarity estimated based on primate genomes and Skmer similarity estimated based on NGS samples of only 1 M, 3M, 5 M, 7M, 9 M, 11 M, 13 M or 15 M reads, respectively. (i) shows pairwise Skmer similarity estimated based on mixed NGS samples. NGS samples of different numbers of reads are colored accordingly. 'Mix' means two NGS samples have different numbers of reads (e.g between 1 M and 5 M or between 7 M and 11 M) and is colored in grey. The root mean squared error (RMSE) and Spearman correlation coefficients (SPC) between pairwise Skmer similarity estimated based on NGS samples and genomes are shown on each subplot.

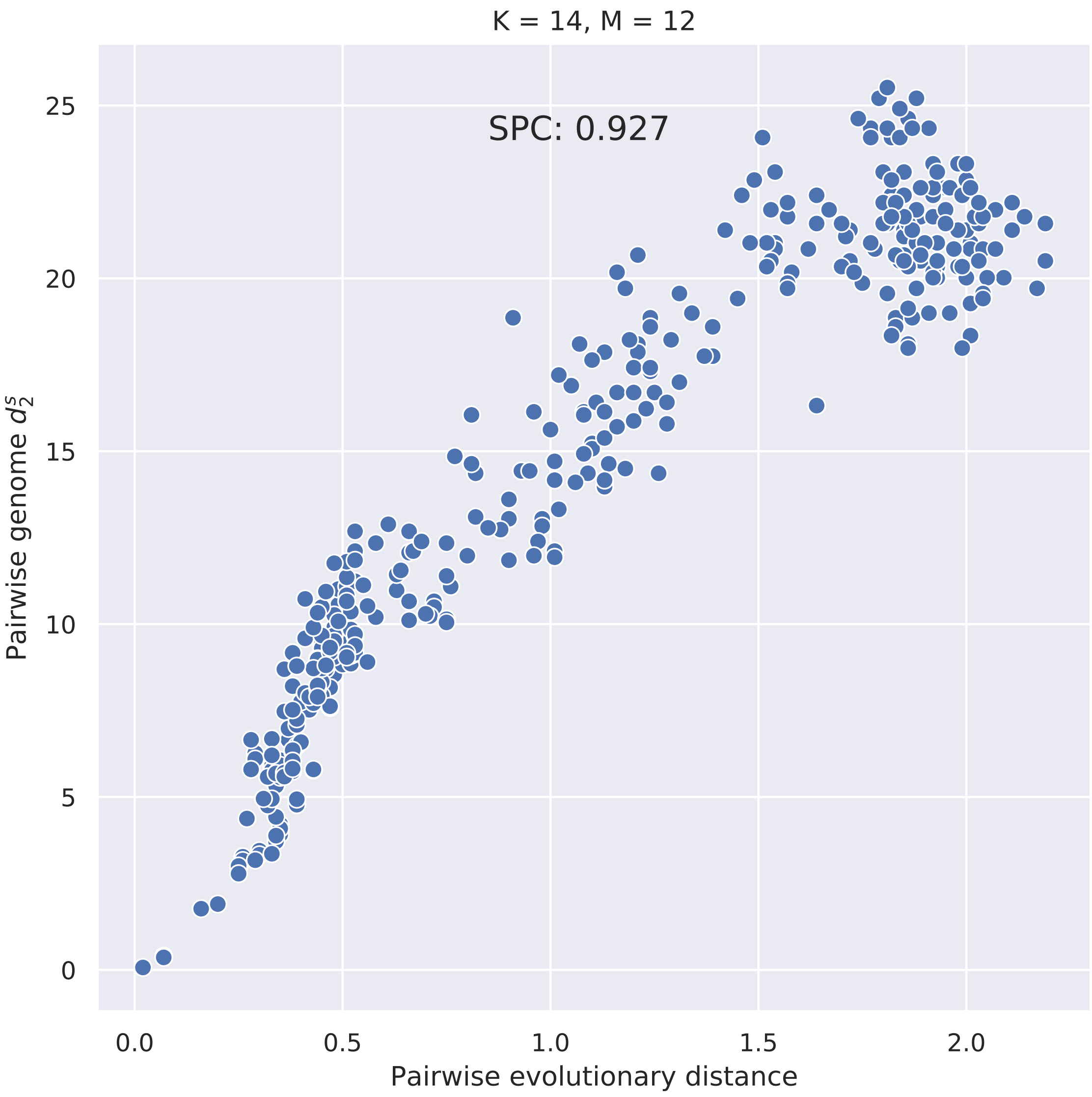

Figure S15: Relationship between pairwise d_2^s using $K = 14$ and evolutionary distances among 28 mammals. X-axis is the pairwise mammalian evolutionary distances estimated by alignment-based method in [4] and Y-axis is the pairwise d_2^s caculated based on mammalian genomes using $K = 14$ and $M = 12$ and has been transformed by $(log(1 - 2 \times d_2^s))^2$ for better visualization. The Spearman correlation coefficient (SPC) is 0.927.

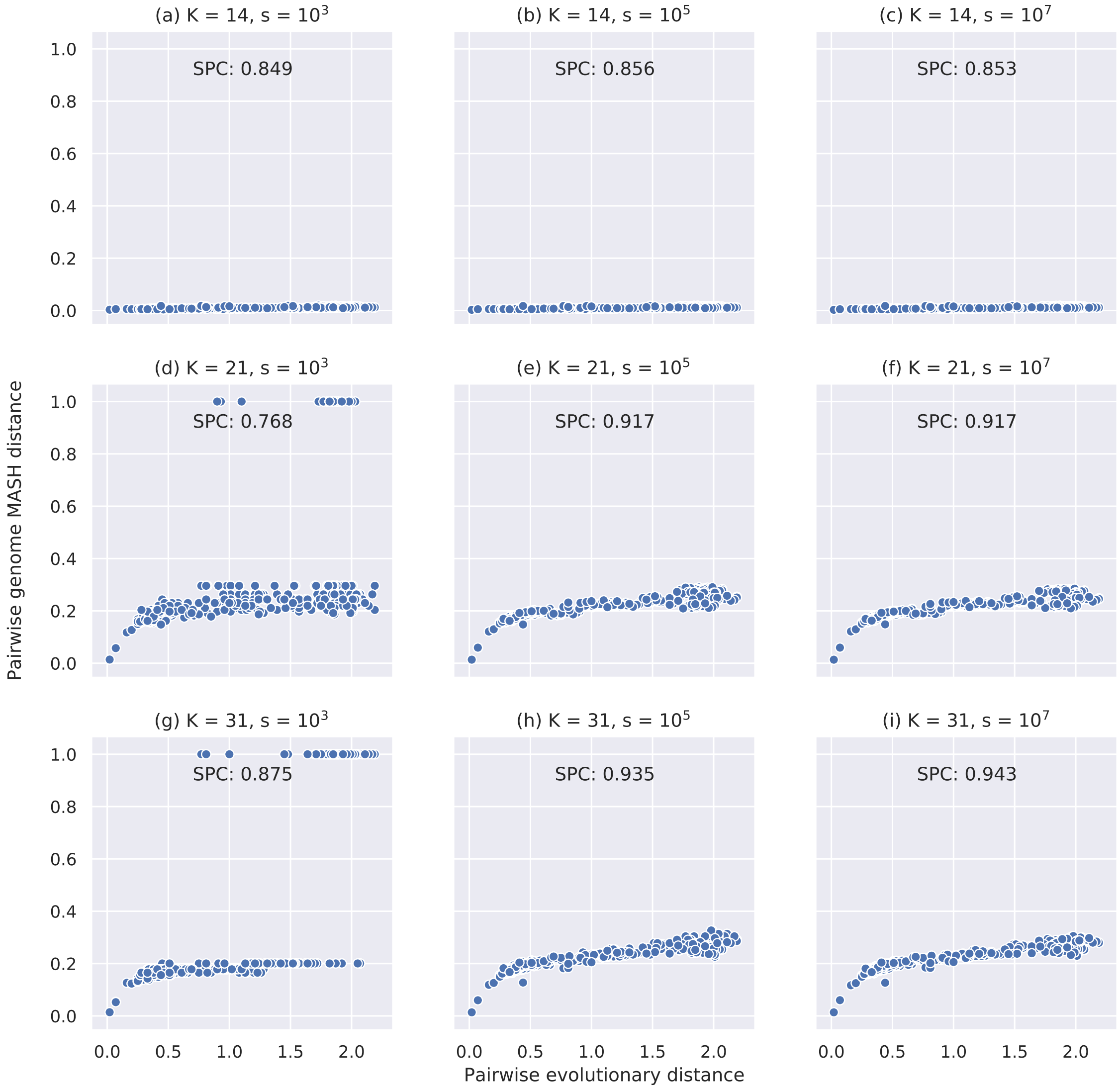

Figure S16: Relationship between pairwise Mash distance using $K = 14$, $K = 21$, $K = 31$ and sketch size $s = 10^3$, $s = 10^5$, $s = 10^7$ and evolutionary distances among 28 mammals. X-axis is the pairwise mammalian evolutionary distances estimated by alignment-based method in [4] and Y-axis is the pairwise Mash distances calculated based on mammalian genomes. The corresponding Spearman correlation coefficients (SPC) for each combination of K and s are shown on the subplot. Mash distances with $K = 31$ and $s = 10^7$ and the evolutionary distances have the highest Spearman correlation coefficient 0.943.

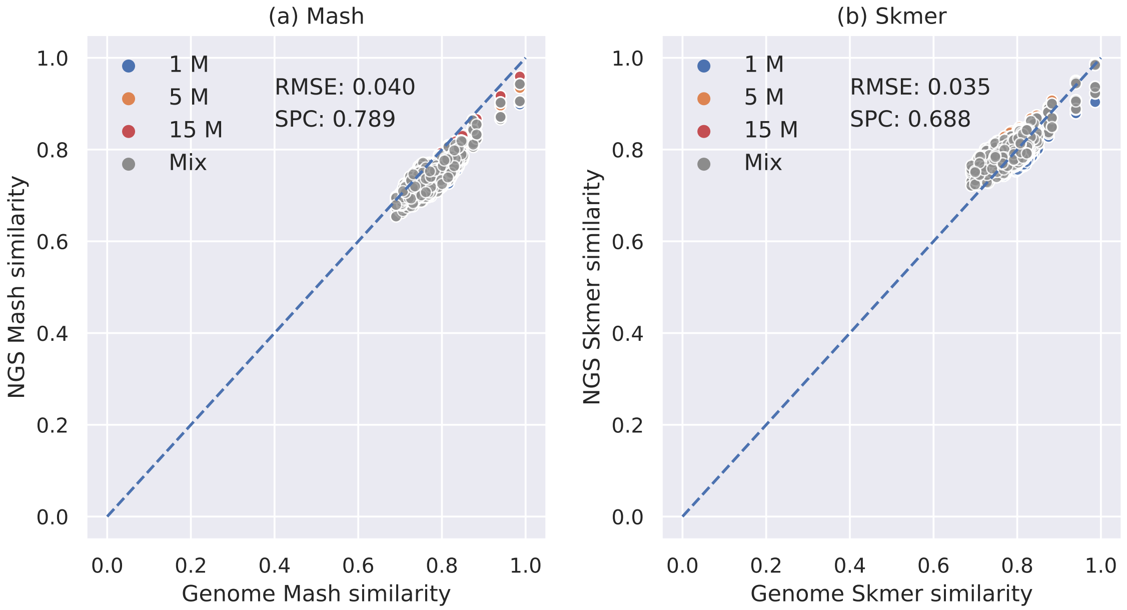

Figure S17: Relationship between pairwise Mash and Skmer similarity estimated using $K = 31$ and sketch size $s = 10^7$ based on 28 mammalian genomes and NGS samples of different numbers of reads. (a) relationship of Mash similarity. (b) relationship of Skmer similarity.

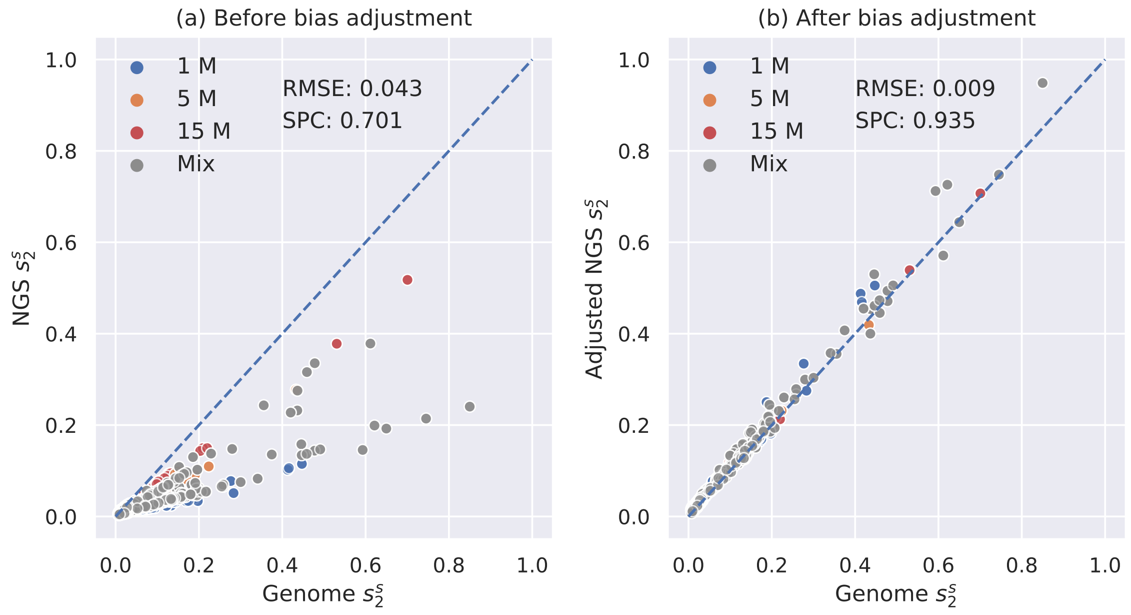

Figure S18: Relationship between pairwise s_2^s estimated using $K = 14$ and $M = 12$ based on 67 vertebrate genomes and NGS samples of different numbers of reads. (a) relationship before bias adjustment. (b) relationship after bias adjustment for NGS s_2^s. The root mean squared error was decreased and the Spearman correlation coeffient between pairwise genome s_2^s and NGS s_2^s was increased after bias adjustment.

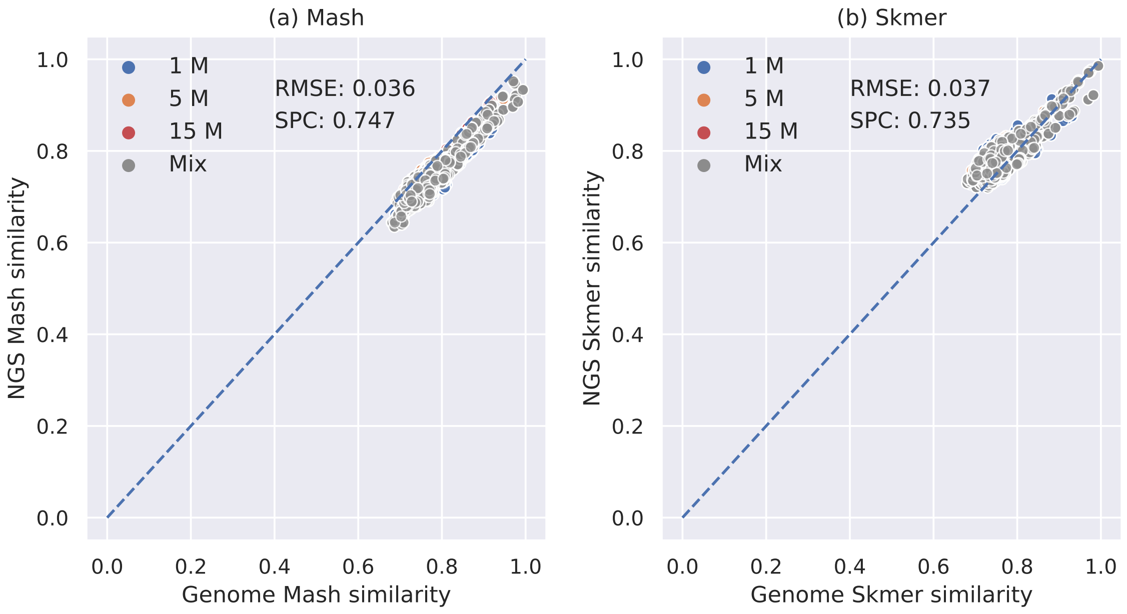

Figure S19: Relationship between pairwise Mash and Skmer similarity estimated using $K = 31$ and sketch size $s = 10^7$ based on 67 vertebrate genomes and NGS samples of different numbers of reads. (a) relationship of Mash similarity. (b) relationship of Skmer similarity.

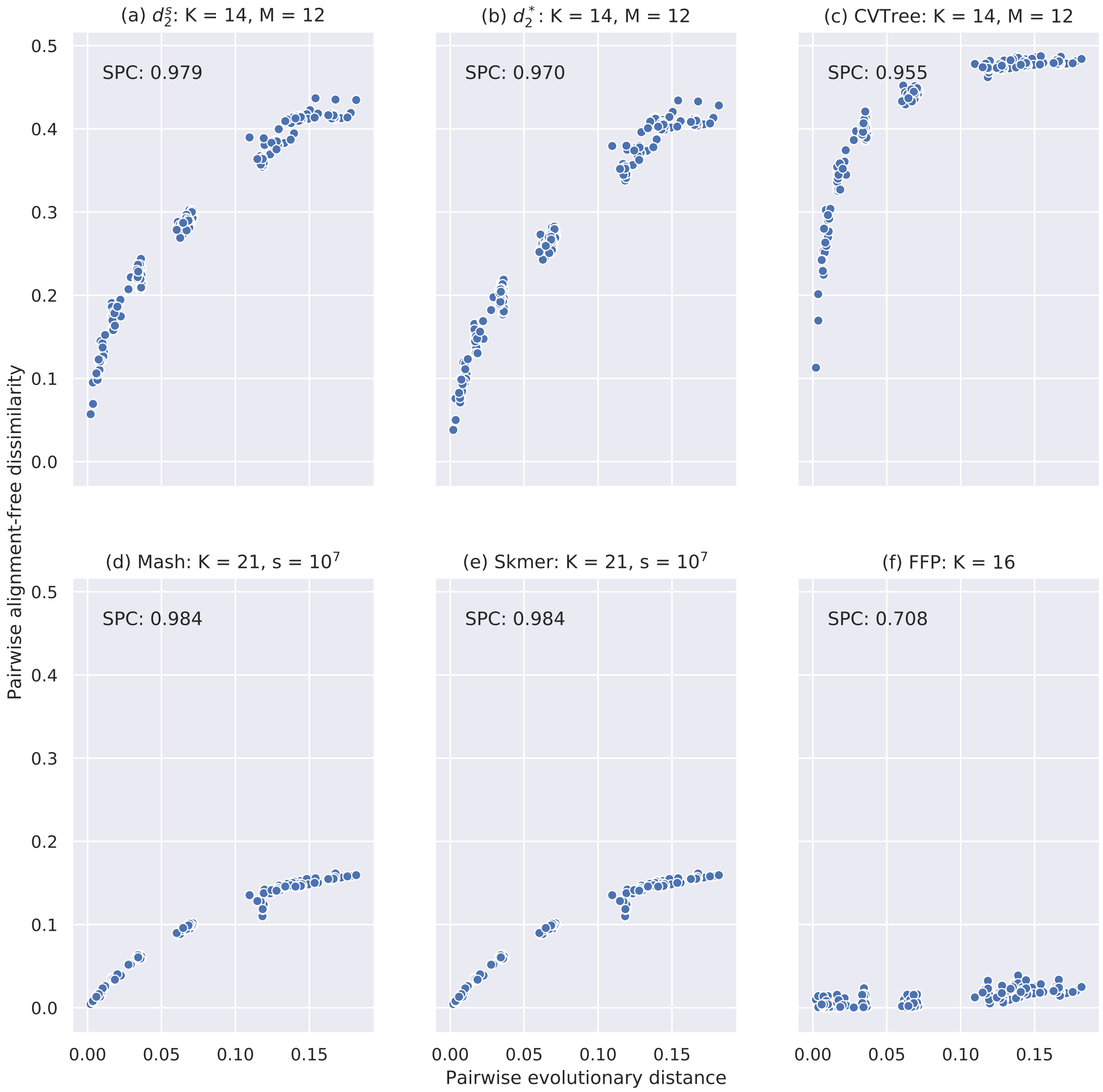

Figure S20: Relationship between different pairwise alignment-free dissimilarity and evolutionary distances among 21 primates: (a) d_2^s with $K = 14$ and $M = 12$. (b) d_2^* with $K = 14$ and $M = 12$. (c) $CVTree$ with $K = 14$ and $M = 12$. (d) Mash with $K = 21$ and $s = 10^7$. (e) Skmer with $K = 21$ and $s = 10^7$. (f) FFP with $K = 16$. X-axis is the pairwise primate evolutionary distances estimated by alignment-based method in [3] and Y-axis is the pairwise alignment-free dissimilarity. The Spearman correlation coefficients (SPC) are shown on each subplot.

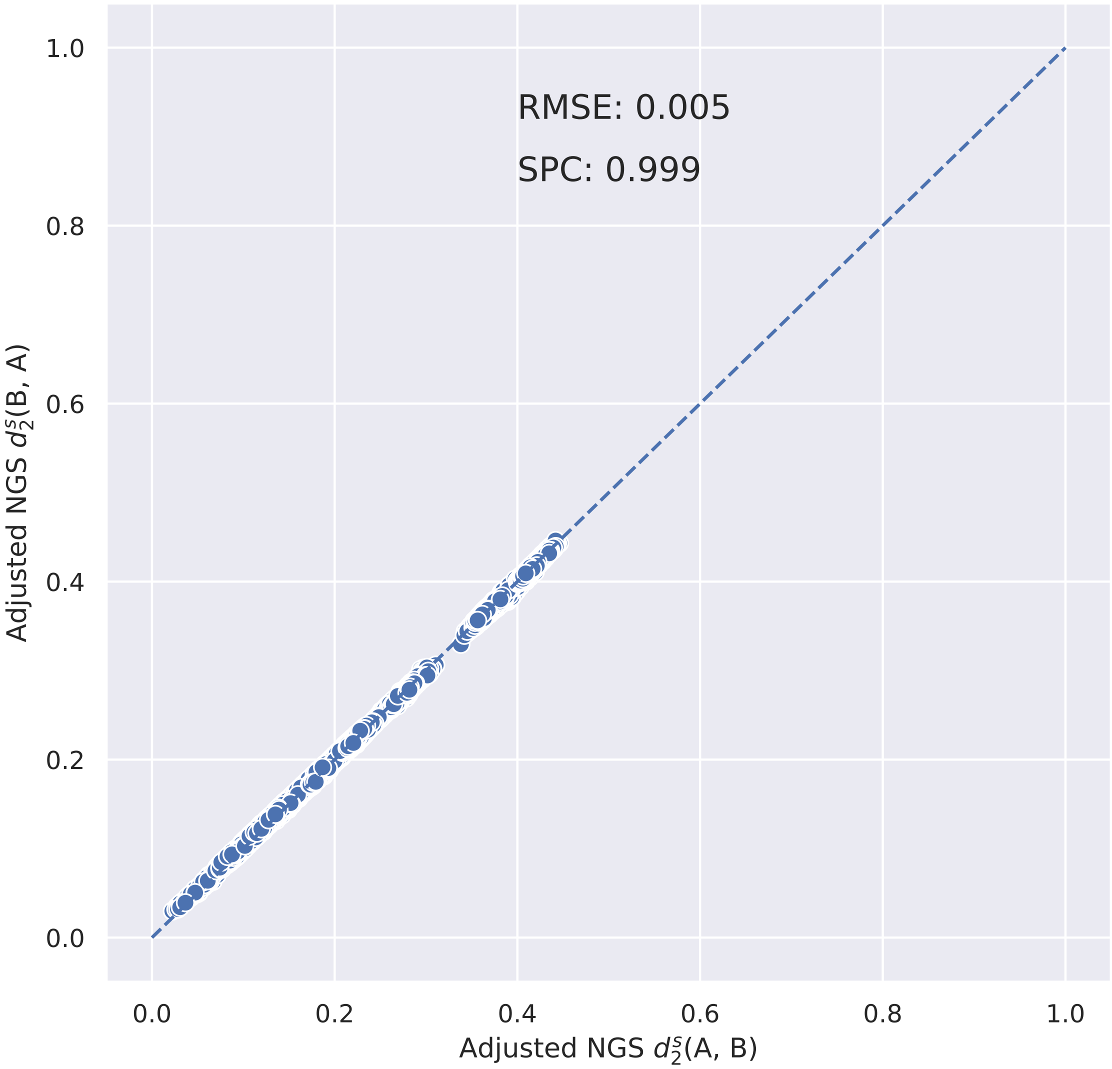

Figure S21: Relationship between pairwise adjusted $d_2^s(A, B)$ and adjusted $d_2^s(B, A)$ based on 21 primates dataset. For any pair of NGS samples A and B, X-axis is the adjusted $d_2^s(A, B)$ and Y-axis is the adjusted $d_2^s(B, A)$. The Spearman correlation coefficient is 0.999 which proves that our model successfully trained $d_2^s(A, B)$ and $d_2^s(B, A)$ symmetrically.

Query size	Reference size	k=1	k=2	k=3	k=4	k=5	k=6	k=7	k=8	k=9	k=10
Samples of 50 Mbp											
1	91	1.00	1.00	1.00	1.00	1.00	1.00	0.93	0.97	0.88	0.94
17	75	1.00	1.00	0.99	0.99	0.97	0.97	0.95	0.96	0.94	0.96
32	60	0.99	0.99	0.97	0.97	0.94	0.95	0.93	0.95	0.93	0.95
47	45	0.98	0.98	0.95	0.96	0.93	0.94	0.93	0.95	0.91	0.93
62	30	0.95	0.95	0.92	0.93	0.90	0.93	0.90	0.92	0.89	0.91
77	15	0.89	0.89	0.85	0.87	0.81	0.82	0.79	0.77	0.73	0.67
Samples of 100 Mbp											
1	91	1.00	1.00	1.00	1.00	1.00	1.00	0.98	1.00	0.96	1.00
17	75	1.00	1.00	0.99	0.99	0.98	0.98	0.97	0.99	0.97	0.99
32	60	1.00	1.00	0.98	0.99	0.97	0.98	0.96	0.97	0.95	0.96
47	45	0.99	0.99	0.97	0.98	0.96	0.97	0.95	0.97	0.95	0.97
62	30	0.98	0.98	0.95	0.96	0.94	0.96	0.93	0.95	0.90	0.91
77	15	0.93	0.93	0.90	0.91	0.86	0.84	0.81	0.78	0.70	0.67
Samples of 300 Mbp											
1	91	1.00	1.00	1.00	1.00	1.00	1.00	1.00	1.00	1.00	1.00
17	75	1.00	1.00	1.00	1.00	1.00	1.00	0.99	1.00	1.00	1.00
32	60	1.00	1.00	0.99	1.00	0.99	1.00	0.98	0.99	0.98	0.99
47	45	1.00	1.00	0.99	0.99	0.98	0.99	0.98	0.99	0.97	0.98
62	30	0.99	0.99	0.97	0.98	0.96	0.97	0.94	0.95	0.91	0.92
77	15	0.96	0.96	0.92	0.93	0.86	0.86	0.81	0.79	0.74	0.71

Table S1: Prediction accuracy using k-NN on 92 white oak dataset based on d_2^* for differnt sequence quantity, query sizes, reference sizes and different numbers of neighbors k used. For each query size and reference size, the dataset was randomly split 100 times and an average prediction accuracy was calculated over 100 splits.

Query size	Reference size	k=1	k=2	k=3	k=4	k=5	k=6	k=7	k=8	k=9	k=10
$K = 12,\ s = 10^3$											
1	91	0.44	0.44	0.40	0.45	0.49	0.47	0.53	0.55	0.53	0.58
17	75	0.47	0.47	0.41	0.48	0.47	0.50	0.52	0.52	0.52	0.53
32	60	0.45	0.45	0.44	0.48	0.47	0.49	0.50	0.51	0.52	0.53
47	45	0.43	0.43	0.44	0.46	0.46	0.48	0.49	0.50	0.49	0.50
62	30	0.42	0.42	0.45	0.47	0.46	0.47	0.47	0.47	0.46	0.46
77	15	0.41	0.41	0.42	0.43	0.43	0.42	0.42	0.43	0.43	0.43
$K = 12,\ s = 10^5$											
1	91	0.60	0.60	0.47	0.45	0.51	0.47	0.48	0.56	0.66	0.67
17	75	0.62	0.62	0.52	0.51	0.55	0.56	0.58	0.63	0.64	0.61
32	60	0.59	0.59	0.56	0.56	0.58	0.60	0.60	0.60	0.60	0.59
47	45	0.59	0.59	0.57	0.58	0.57	0.59	0.58	0.58	0.58	0.57
62	30	0.55	0.55	0.55	0.56	0.54	0.55	0.54	0.53	0.53	0.52
77	15	0.52	0.52	0.49	0.50	0.48	0.48	0.47	0.47	0.46	0.45
$K = 12,\ s = 10^7$											
1	91	0.54	0.54	0.58	0.55	0.61	0.60	0.58	0.75	0.74	0.61
17	75	0.62	0.62	0.57	0.55	0.58	0.62	0.57	0.63	0.62	0.58
32	60	0.59	0.59	0.57	0.57	0.59	0.60	0.58	0.59	0.58	0.56
47	45	0.59	0.59	0.59	0.58	0.58	0.58	0.56	0.58	0.57	0.56
62	30	0.56	0.56	0.56	0.55	0.55	0.54	0.54	0.54	0.52	0.51
77	15	0.54	0.54	0.52	0.51	0.48	0.48	0.47	0.47	0.46	0.46
$K = 21,\ s = 10^3$											
1	91	0.63	0.63	0.57	0.63	0.57	0.58	0.54	0.60	0.56	0.59
17	75	0.63	0.63	0.61	0.63	0.61	0.62	0.58	0.61	0.58	0.62
32	60	0.63	0.63	0.62	0.63	0.62	0.61	0.60	0.61	0.60	0.62
47	45	0.60	0.60	0.59	0.60	0.58	0.59	0.59	0.61	0.60	0.62
62	30	0.59	0.59	0.58	0.58	0.57	0.59	0.58	0.58	0.59	0.60
77	15	0.53	0.53	0.53	0.55	0.54	0.54	0.54	0.53	0.53	0.53
$K = 21,\ s = 10^5$											
1	91	0.82	0.82	0.72	0.72	0.53	0.55	0.46	0.48	0.48	0.48
17	75	0.83	0.83	0.68	0.68	0.57	0.58	0.51	0.53	0.52	0.54
32	60	0.79	0.79	0.64	0.65	0.55	0.57	0.53	0.56	0.55	0.58
47	45	0.75	0.75	0.59	0.61	0.54	0.57	0.54	0.59	0.58	0.61
62	30	0.65	0.65	0.54	0.58	0.54	0.59	0.58	0.61	0.62	0.64
77	15	0.59	0.59	0.55	0.57	0.56	0.58	0.57	0.56	0.54	0.52
$K = 21,\ s = 10^7$											
1	91	0.92	0.92	0.73	0.75	0.53	0.54	0.48	0.48	0.43	0.43
17	75	0.85	0.85	0.61	0.63	0.49	0.52	0.47	0.48	0.47	0.50
32	60	0.80	0.80	0.58	0.61	0.51	0.54	0.50	0.54	0.53	0.57

47	45	0.74	0.74	0.55	0.58	0.51	0.55	0.55	0.59	0.59	0.62
62	30	0.68	0.68	0.52	0.57	0.52	0.58	0.57	0.62	0.61	0.64
77	15	0.58	0.58	0.55	0.59	0.57	0.58	0.58	0.57	0.55	0.55
$K = 31$, $s = 10^3$											
1	91	0.66	0.66	0.64	0.67	0.65	0.62	0.62	0.62	0.58	0.58
17	75	0.66	0.66	0.68	0.67	0.66	0.65	0.62	0.62	0.59	0.59
32	60	0.66	0.66	0.67	0.65	0.63	0.63	0.60	0.61	0.59	0.60
47	45	0.63	0.63	0.62	0.62	0.59	0.59	0.57	0.57	0.56	0.56
62	30	0.61	0.61	0.58	0.59	0.57	0.57	0.56	0.57	0.55	0.56
77	15	0.57	0.57	0.55	0.56	0.53	0.54	0.52	0.52	0.52	0.49
$K = 31$, $s = 10^5$											
1	91	0.98	0.98	0.82	0.85	0.77	0.77	0.62	0.60	0.59	0.59
17	75	0.92	0.92	0.76	0.78	0.69	0.70	0.62	0.63	0.61	0.61
32	60	0.86	0.86	0.72	0.73	0.64	0.64	0.60	0.61	0.60	0.61
47	45	0.82	0.82	0.68	0.68	0.61	0.63	0.60	0.62	0.61	0.64
62	30	0.75	0.75	0.65	0.67	0.62	0.65	0.63	0.67	0.65	0.67
77	15	0.63	0.63	0.57	0.60	0.58	0.60	0.57	0.56	0.55	0.54
$K = 31$, $s = 10^7$											
1	91	0.96	0.96	0.78	0.84	0.67	0.72	0.53	0.52	0.53	0.52
17	75	0.93	0.93	0.77	0.80	0.66	0.69	0.60	0.61	0.60	0.61
32	60	0.88	0.88	0.68	0.72	0.61	0.63	0.57	0.59	0.57	0.59
47	45	0.83	0.83	0.67	0.69	0.61	0.63	0.60	0.63	0.62	0.65
62	30	0.75	0.75	0.63	0.64	0.60	0.62	0.60	0.63	0.63	0.65
77	15	0.64	0.64	0.58	0.60	0.59	0.61	0.59	0.60	0.59	0.57

Table S2: Prediction accuracy using k-NN on 92 white oak dataset of mixed sequence quantity based on Mash using different kmer lengths K and sketch sizes s for different query sizes, reference sizes and different numbers of neighbors k used. For each query size and reference size, the dataset was randomly split 100 times and an average prediction accuracy was calculated over 100 splits.

Query size	Reference size	k=1	k=2	k=3	k=4	k=5	k=6	k=7	k=8	k=9	k=10
$K = 12,\ s = 10^3$											
1	91	0.44	0.44	0.44	0.44	0.44	0.44	0.44	0.44	0.44	0.44
17	75	0.35	0.35	0.35	0.35	0.35	0.35	0.35	0.35	0.35	0.35
32	60	0.36	0.36	0.36	0.36	0.36	0.36	0.36	0.36	0.36	0.36
47	45	0.36	0.36	0.36	0.36	0.36	0.36	0.36	0.36	0.36	0.36
62	30	0.36	0.36	0.36	0.36	0.36	0.36	0.36	0.36	0.35	0.35
77	15	0.36	0.36	0.35	0.36	0.34	0.35	0.34	0.35	0.35	0.36
$K = 12,\ s = 10^5$											
1	91	0.39	0.39	0.39	0.39	0.39	0.39	0.39	0.39	0.39	0.39
17	75	0.37	0.37	0.37	0.37	0.37	0.37	0.37	0.37	0.37	0.37
32	60	0.37	0.37	0.37	0.37	0.37	0.37	0.37	0.37	0.37	0.37
47	45	0.35	0.35	0.35	0.35	0.35	0.35	0.35	0.35	0.35	0.35
62	30	0.36	0.36	0.36	0.36	0.36	0.36	0.36	0.36	0.36	0.36
77	15	0.36	0.36	0.36	0.36	0.36	0.36	0.36	0.36	0.36	0.37
$K = 12,\ s = 10^7$											
1	91	0.38	0.38	0.38	0.38	0.38	0.38	0.38	0.38	0.38	0.38
17	75	0.35	0.35	0.35	0.35	0.35	0.35	0.35	0.35	0.35	0.35
32	60	0.37	0.37	0.37	0.37	0.37	0.37	0.37	0.37	0.37	0.37
47	45	0.35	0.35	0.35	0.35	0.35	0.35	0.35	0.35	0.35	0.35
62	30	0.36	0.36	0.36	0.36	0.36	0.36	0.36	0.36	0.36	0.36
77	15	0.36	0.36	0.36	0.36	0.36	0.36	0.35	0.36	0.36	0.36
$K = 21,\ s = 10^3$											
1	91	0.77	0.77	0.66	0.72	0.70	0.74	0.69	0.67	0.57	0.58
17	75	0.67	0.67	0.61	0.64	0.61	0.60	0.57	0.57	0.55	0.55
32	60	0.65	0.65	0.61	0.61	0.58	0.58	0.57	0.57	0.57	0.56
47	45	0.62	0.62	0.59	0.61	0.57	0.57	0.56	0.55	0.54	0.53
62	30	0.58	0.58	0.55	0.54	0.53	0.52	0.51	0.50	0.51	0.51
77	15	0.51	0.51	0.48	0.47	0.48	0.51	0.51	0.52	0.54	0.53
$K = 21,\ s = 10^5$											
1	91	0.87	0.87	0.88	0.83	0.67	0.66	0.56	0.61	0.59	0.48
17	75	0.86	0.86	0.84	0.81	0.72	0.70	0.60	0.60	0.55	0.51
32	60	0.83	0.83	0.78	0.76	0.68	0.64	0.57	0.55	0.49	0.46
47	45	0.79	0.79	0.68	0.63	0.57	0.53	0.47	0.45	0.44	0.43
62	30	0.69	0.69	0.58	0.53	0.48	0.45	0.42	0.45	0.48	0.49
77	15	0.55	0.55	0.45	0.45	0.46	0.51	0.51	0.53	0.52	0.54
$K = 21,\ s = 10^7$											
1	91	0.94	0.94	0.87	0.84	0.74	0.74	0.72	0.75	0.65	0.51
17	75	0.88	0.88	0.83	0.81	0.73	0.71	0.64	0.61	0.52	0.47
32	60	0.85	0.85	0.79	0.75	0.68	0.64	0.54	0.51	0.46	0.43

47	45	0.80	0.80	0.70	0.66	0.59	0.53	0.45	0.43	0.41	0.43
62	30	0.74	0.74	0.60	0.52	0.49	0.46	0.45	0.46	0.50	0.53
77	15	0.58	0.58	0.46	0.45	0.48	0.52	0.52	0.53	0.54	0.56
$K = 31,\ s = 10^3$											
1	91	0.85	0.85	0.90	0.91	0.88	0.92	0.77	0.79	0.79	0.77
17	75	0.78	0.78	0.83	0.84	0.78	0.78	0.74	0.75	0.74	0.74
32	60	0.77	0.77	0.79	0.80	0.73	0.74	0.71	0.71	0.70	0.69
47	45	0.74	0.74	0.72	0.73	0.69	0.70	0.68	0.68	0.66	0.65
62	30	0.69	0.69	0.66	0.66	0.62	0.61	0.57	0.55	0.53	0.53
77	15	0.59	0.59	0.54	0.51	0.51	0.49	0.49	0.51	0.52	0.52
$K = 31,\ s = 10^5$											
1	91	1.00	1.00	1.00	1.00	0.97	0.97	0.95	0.95	0.93	0.86
17	75	0.99	0.99	0.96	0.95	0.92	0.91	0.87	0.84	0.80	0.78
32	60	0.98	0.98	0.94	0.93	0.88	0.86	0.81	0.78	0.75	0.72
47	45	0.96	0.96	0.91	0.89	0.82	0.78	0.72	0.70	0.67	0.65
62	30	0.88	0.88	0.78	0.73	0.67	0.62	0.58	0.58	0.55	0.53
77	15	0.71	0.71	0.57	0.53	0.53	0.51	0.50	0.52	0.53	0.55
$K = 31,\ s = 10^7$											
1	91	1.00	1.00	1.00	0.98	0.95	0.95	0.93	0.93	0.91	0.91
17	75	1.00	1.00	0.98	0.97	0.94	0.94	0.90	0.89	0.85	0.82
32	60	0.98	0.98	0.94	0.93	0.88	0.86	0.81	0.80	0.75	0.72
47	45	0.97	0.97	0.90	0.88	0.82	0.79	0.74	0.71	0.68	0.64
62	30	0.90	0.90	0.79	0.75	0.69	0.66	0.58	0.58	0.57	0.55
77	15	0.73	0.73	0.60	0.55	0.56	0.54	0.53	0.56	0.57	0.56

Table S3: Prediction accuracy using k-NN on 92 white oak dataset of mixed sequence quantity based on Skmer using different kmer lengths K and sketch sizes s for different query sizes, reference sizes and different numbers of neighbors k used. For each query size and reference size, the dataset was randomly split 100 times and an average prediction accuracy was calculated over 100 splits.

	Counting (min)	Calculation (min)	Total Time (min)	Memory (GB)
Cafe-d_2^s	2397.3	4023.8	6421.1	28.9
Afann-d_2^s	78.4	27.3	105.7	6.4
Cafe-d_2^*	2397.3	4039.2	6436.5	28.9
Afann-d_2^*	78.4	13.3	91.7	4.1
Afann-d_2^*-fast	78.4	0.9	79.3	45.1
Cafe-$CVTree$	2397.3	3921.8	6319.1	28.9
Afann-$CVTree$	78.4	14.5	92.9	4.1
Afann-$CVTree$-fast	78.4	1.1	79.5	45.1
Mash^{min}	63.5	0.1	63.6	0.6
Mash^{opt}	100.3	1.5	101.8	4.4
Skmer^{min}	NA	NA	69.4	0.6
Skmer^{opt}	NA	NA	121.6	1.1
FFP	94.9	0.1	95.0	0.08

Table S4: Kmer counting time, dissimilarity calculation time and total time as well as memory usage used by Cafe and Afann to calculate the pairwise d_2^s, d_2^* and $CVTree$ using $K = 14$ and $M = 12$ among a dataset of 21 primate genomes. Afann-d_2^*-fast and Afann-$CVTree$-fast stand for the fast mode of d_2^* and $CVTree$ supported in Afann. Running time and memory usage of Mash, Skmer and FFP were also included. Mash^{min} and Skmer^{min} used $K = 14$ and $s = 10^3$ which require the minimum computing power. Mash^{opt} and Skmer^{opt} used $K = 21$ and $s = 10^7$ which have the optimal performance among Mash and Skmer using different combinations of kmer lengths and sketch sizes as shown in Figure S9. FFP used $K = 16$.

Species	Assembly accession number	Total sequence length (Mb)
Daubentonia madagascariensis	GCA_000241425.1	2855.37
Nasalis larvatus	GCA_000772465.1	3011.97
Eulemur macaco	GCA_001262655.1	2119.88
Homo sapiens	GCF_000001405.39	3099.73
Pongo abelii	GCF_002880775.1	3441.24
Callithrix jacchus	GCF_000004665.1	2914.96
Nomascus leucogenys	GCF_000146795.2	2962.06
Gorilla gorilla gorilla	GCF_000151905.2	3029.54
Carlito syrichta	GCF_000164805.1	3453.86
Otolemur garnettii	GCF_000181295.1	2519.72
Saimiri boliviensis boliviensis	GCF_000235385.1	2608.59
Pan paniscus	GCF_000258655.2	3286.64
Papio anubis	GCF_000264685.3	2948.40
Macaca fascicularis	GCF_000364345.1	2946.84
Galeopterus variegatus	GCF_000696425.1	3187.66
Macaca mulatta	GCF_003339765.1	3236.21
Colobus angolensis palliatus	GCF_000951035.1	2970.12
Mandrillus leucophaeus	GCF_000951045.1	3061.99
Aotus nancymaae	GCF_000952055.2	2861.68
Macaca nemestrina	GCF_000956065.1	2948.70
Chlorocebus sabaeus	GCF_000409795.2	2789.66

Table S5: Species names, assembly accession numbers and total sequence lengths of 21 primate genomes with known pairwise evolutionary distances estimated by alignment-based method in [3].

Species	Assembly accession number	Total sequence length (Mb)
Homo sapiens	GCF_000001405.39	3099.73
Pan troglodytes	GCF_002880755.1	3309.56
Macaca mulatta	GCF_003339765.1	2969.97
Otolemur garnettii	GCF_000181295.1	2519.72
Tupaia belangeri	GCA_000181375.1	2137.23
Rattus norvegicus	GCF_000001895.5	2909.70
Mus musculus	GCF_000001635.26	2730.86
Cavia porcellus	GCF_000151735.1	2723.22
Oryctolagus cuniculus	GCF_000003625.3	2737.46
Sorex araneus	GCF_000181275.1	2423.16
Erinaceus europaeus	GCF_000296755.1	2715.72
Canis lupus familiaris	GCF_000002285.3	2410.98
Felis catus	GCF_000181335.3	2455.54
Equus caballus	GCF_002863925.1	2474.91
Bos taurus	GCF_002263795.1	2983.31
Dasypus novemcinctus	GCF_000208655.1	3631.52
Loxodonta africana	GCF_000001905.1	3196.74
Echinops telfairi	GCF_000313985.1	2947.02
Monodelphis domestica	GCF_000002295.2	3598.44
Ornithorhynchus anatinus	GCF_004115215.1	1995.61
Gallus gallus	GCF_000002315.6	1046.93
Anolis carolinensis	GCF_000090745.1	1799.14
Xenopus tropicalis	GCF_000004195.3	1511.72
Tetraodon nigroviridis	GCA_000180735.1	342.40
Takifugu rubripes	GCF_901000725.2	391.47
Gasterosteus aculeatus	GCA_006229165.1	467.45
Oryzias latipes	GCF_002234675.1	734.06
Danio rerio	GCF_000002035.6	1412.46

Table S6: Species names, assembly accession numbers and total sequence lengths of 28 mammlian genomes with known pairwise evolutionary distances estimated by alignment-based method in [4].

Run accession number	Number of bases (Mbp)	Continental origin
SRR2053033	685.23	North America
SRR2053034	917.57	North America
SRR2053035	1268.47	North America
SRR2053036	694.58	North America
SRR2053037	1024.22	North America
SRR2053038	869.04	North America
SRR2053039	626.5	North America
SRR2053040	803.69	North America
SRR2053041	616.33	North America
SRR2053042	1079	North America
SRR2053043	906.57	North America
SRR2053044	742.13	North America
SRR2053045	790.02	North America
SRR2053046	781.9	North America
SRR2053047	885.39	North America
SRR2053048	491.75	North America
SRR2053049	883.87	North America
SRR2053050	915.02	North America
SRR2053051	944.57	North America
SRR2053052	642.98	North America
SRR2053053	894.36	North America
SRR2053054	643.77	North America
SRR2053055	1493.05	North America
SRR2053056	638.61	North America
SRR2053057	554.35	North America
SRR2053058	717.96	North America
SRR2053059	688.58	North America
SRR2053061	857.74	North America
SRR2053062	767	North America
SRR2053063	845.86	North America
SRR2053064	927.41	North America
SRR2053065	390.22	North America
SRR2053067	1170.13	North America
SRR2053068	1209.42	North America
SRR2053069	955.16	North America
SRR2053070	845.2	North America
SRR2053071	524.75	North America
SRR2053072	1245.94	Asia
SRR2053073	1688.66	Europe

SRR2053074	959.47	North America
SRR2053075	1260.95	North America
SRR2053076	934.44	North America
SRR2053077	1764.53	Europe
SRR2053078	818.77	North America
SRR2053079	586.82	North America
SRR2053081	651.53	Asia
SRR2053083	732.85	Asia
SRR2053084	715.3	Asia
SRR2053085	867.69	Asia
SRR2053086	908.06	Asia
SRR2053087	892.59	Asia
SRR2053088	859.19	Asia
SRR2053089	463.45	Asia
SRR2053090	549.38	Asia
SRR2053091	1170.22	Asia
SRR2053092	612.1	Asia
SRR2053093	1134.81	Asia
SRR2053094	410.8	Asia
SRR2053095	593.49	Asia
SRR2053096	542.02	Asia
SRR2053097	482.64	Asia
SRR2053098	671.66	Asia
SRR2053099	1414.04	Asia
SRR2053100	378.52	Asia
SRR2053101	638.39	Asia
SRR2053102	637.86	Asia
SRR2053103	1539.57	Asia
SRR2053104	1048.44	Asia
SRR2053105	840.4	Asia
SRR2053106	519.54	Asia
SRR2053107	475.99	Asia
SRR2053108	434.93	Asia
SRR2053109	533.56	Europe
SRR2053110	1275.52	Europe
SRR2053111	858.59	Europe
SRR2053112	828.15	Europe
SRR2053113	388.95	Europe
SRR2053114	1048.06	Europe
SRR2053115	1851.59	Europe
SRR2053116	643.04	Europe

SRR2053117	676.9	Europe
SRR2053118	783.79	Europe
SRR2053119	699.55	Asia
SRR2053120	768.01	Asia
SRR2053121	823.06	Asia
SRR2053122	664.5	Asia
SRR2053126	1032.69	Europe
SRR2053127	643.54	Europe
SRR2053128	1203.51	Europe
SRR2053129	1278.05	North America
SRR2053130	1849.31	Asia
SRR2053131	1581	Europe

Table S7: Run accession numbers, number of bases and continental origins of 92 white oak NGS samples from NCBI BioProject PRJNA269970. Continental origins are defined based on samples' coordinates according to [5].

Species	Class	Assembly accession	Total sequence length (Mb)
Cyprinus carpio	Fish	GCF_000951615.1	1713.66
Carassius auratus	Fish	GCF_003368295.1	1820.64
Cyprinodon nevadensis	Fish	GCA_000776015.1	1011.85
Seriola lalandi	Fish	GCF_002814215.1	732.51
Channa argus	Fish	GCA_004786185.1	644.13
Astyanax mexicanus	Fish	GCF_000372685.2	1335.24
Neolamprologus brichardi	Fish	GCF_000239395.1	847.91
Latimeria chalumnae	Fish	GCF_000225785.1	2860.59
Oryzias latipes	Fish	GCF_002234675.1	734.06
Oncorhynchus mykiss	Fish	GCF_002163495.1	2179.00
Poecilia reticulata	Fish	GCF_000633615.1	731.62
Xiphophorus maculatus	Fish	GCF_002775205.1	704.32
Nothobranchius furzeri	Fish	GCF_001465895.1	1242.52
Maylandia zebra	Fish	GCF_000238955.4	957.49
Salmo salar	Fish	GCF_000233375.1	2966.89
Pyxicephalus adspersus	Amphibian	GCA_004786255.1	1563.37
Nanorana parkeri	Amphibian	GCF_000935625.1	2053.87
Rana catesbeiana	Amphibian	GCA_002284835.2	6250.35
Rhinella marina	Amphibian	GCA_900303285.1	2551.76
Rhinatrema bivittatum	Amphibian	GCF_901001135.1	5319.24
Xenopus laevis	Amphibian	GCF_001663975.1	2718.43
Xenopus tropicalis	Amphibian	GCF_000004195.3	1440.40
Terrapene carolina	Reptile	GCF_002925995.2	2571.27
Crotalus viridis	Reptile	GCA_003400415.2	1340.20
Malaclemys terrapin	Reptile	GCA_001728815.2	2439.75
Vipera berus	Reptile	GCA_000800605.1	1532.39
Chrysemys picta	Reptile	GCF_000241765.3	2365.77
Dermochelys coriacea	Reptile	GCA_006547105.1	2154.17
Varanus komodoensis	Reptile	GCA_004798865.1	1507.95
Hydrophis melanocephalus	Reptile	GCA_004320005.1	1402.64
Emydocephalus ijimae	Reptile	GCA_004319985.1	1625.20
Hydrophis hardwickii	Reptile	GCA_004023765.1	1296.39
Salvator merianae	Reptile	GCA_003586115.2	2068.17
Lacerta viridis	Reptile	GCA_900245905.1	1439.84
Gopherus agassizii	Reptile	GCA_002896415.1	2184.97
Laticauda colubrina	Reptile	GCA_004320045.1	2024.69
Crocodylus porosus	Reptile	GCF_001723895.1	2049.54
Phylloscopus trochiloides	Bird	GCA_001655095.1	1003.33
Aquila chrysaetos	Bird	GCF_000766835.1	1192.74

Hirundo rustica	Bird	GCA_003692655.1	1213.74
Limosa lapponica	Bird	GCA_002844005.1	1034.77
Saxicola maurus	Bird	GCA_900205225.1	1020.37
Strix occidentalis	Bird	GCA_002372975.1	1255.54
Himantopus himantopus	Bird	GCA_003993805.1	1116.81
Patagioenas fasciata	Bird	GCA_002029285.1	1089.15
Lagopus muta	Bird	GCA_004320205.1	1002.56
Tympanuchus cupido	Bird	GCA_001870855.1	983.78
Lonchura striata	Bird	GCF_002197715.1	1060.17
Zosterops lateralis	Bird	GCA_001281735.1	1036.00
Buceros rhinoceros	Bird	GCF_000710305.1	1065.78
Chlamydotis undulata	Bird	GCA_003400225.1	1307.64
Phoenicopterus ruber	Bird	GCA_000687265.1	1132.18
Canis lupus	Mammal	GCF_003254725.1	2439.83
Sus scrofa	Mammal	GCF_000003025.6	2501.91
Mus musculus	Mammal	GCF_000001635.26	2730.86
Odocoileus hemionus	Mammal	GCA_004115125.1	2343.70
Rhinolophus ferrumequinum	Mammal	GCA_004115265.2	2075.79
Eulemur fulvus	Mammal	GCA_004027275.1	2748.89
Panthera tigris	Mammal	GCF_000464555.1	2391.08
Enhydra lutris	Mammal	GCF_002288905.1	2455.28
Homo sapiens	Mammal	GCF_000001405.39	3099.71
Solenodon paradoxus	Mammal	GCA_004363575.1	2109.88
Cebus capucinus	Mammal	GCF_001604975.1	2717.70
Marmota marmota	Mammal	GCF_001458135.1	2510.59
Neophocaena asiaeorientalis	Mammal	GCF_003031525.1	2284.63
Macaca fuscata	Mammal	GCA_003118495.1	2930.71
Ursus arctos	Mammal	GCF_003584765.1	2328.66

Table S8: Species names, classes, assembly accession numbers and total sequence lengths of 67 vertebrate genomes downloaded from NCBI.

References

[1] Ji Qi, Hong Luo, and Bailin Hao. CVTree: a phylogenetic tree reconstruction tool based on whole genomes. *Nucleic Acids Research*, 32(suppl_2):W45–W47, 2004.

[2] Gesine Reinert, David Chew, Fengzhu Sun, and Michael S Waterman. Alignment-free sequence comparison (i): statistics and power. *Journal of Computational Biology*, 16(12):1615–1634, 2009.

[3] Polina Perelman, Warren E Johnson, Christian Roos, Hector N Seuánez, Julie E Horvath, Miguel AM Moreira, Bailey Kessing, Joan Pontius, Melody Roelke, Yves Rumpler, et al. A molecular phylogeny of living primates. *PLOS Genetics*, 7(3):e1001342, 2011.

[4] Webb Miller, Kate Rosenbloom, Ross C Hardison, Minmei Hou, James Taylor, Brian Raney, Richard Burhans, David C King, Robert Baertsch, Daniel Blankenberg, et al. 28-way vertebrate alignment and conservation track in the UCSC genome browser. *Genome Research*, 17(12):1797–1808, 2007.

[5] Kujin Tang, Jie Ren, Richard Cronn, David L Erickson, Brook G Milligan, Meaghan Parker-Forney, John L Spouge, and Fengzhu Sun. Alignment-free genome comparison enables accurate geographic sourcing of white oak DNA. *BMC Genomics*, 19(1):896, 2018.
