# Supplementary material for: Afann: bias adjustment for alignment-free sequence comparison based on sequencing data using neural network regression
Source: Genome Biol. 2019 Dec 4;20:266. doi: 10.1186/s13059-019-1872-3 (PMC6891986; doi:10.1186/s13059-019-1872-3)
Supplement: Supplementary file 2 — Additional file 2 Review history. [file 13059_2019_1872_MOESM2_ESM.docx]

**Review history**

**First round**

**Reviewer 1**

In this paper, the authors proposed a novel regression-based approach to adjusting the bias in comparing two genomes by using their corresponding NGS samples without assembly and alignment. They hypothesized that the dissimilarity of two genomes can be accurately predicted by a regression model of three factors: the dissimilarity calculated by using NGS samples and the features of two NGS samples. To obtain the regression function, a neural-network model is proposed and the model parameters are estimated by using a supervised-learning based approach. The experiment performed on two independent datasets showed that the proposed approach can improve the prediction performance. The authors also demonstrated the generalization capability of the proposed approach by applying it to independent datasets. Overall, this paper is well-written, well-structured and easy to follow. Before the paper can be accepted for publication, there are a couple of issues needed to be addressed.

Major issues

1.	There exist a dozen alignment-free based methods. The authors need to compare the proposed approach with other alignment-free based approaches. It is not sufficient to only compare the results obtained before and after bias adjustment.

2.	It is not clear to me whether using the down-sampling procedure would improve the performance of the proposed approach when comparing two samples with different numbers of reads, that is, in the "Mix" case. For example, if we want to compare two samples with 15M and 9M reads, respectively. Figure 3(i) shows that the proposed approach without down-sampling has RMSE = 0.022 and SPC = 0.989. However, if we first down-sample the 15M sample to 9M and then compare the two samples, Figure 3(e) suggests that the proposed method achieved a better performance (RMSE = 0.011, SPC = 0.999). While the authors claimed that the down-sampling procedure would discard too many reads, leading to worse performance, I could only draw the opposite conclusion based on the result presented in Figure 3.

Minor issues

1.	Although the authors ensured that d(A, B) = d(B, A) by training on both samples with the same target, how close are they (d(A, B) and d(B, A)) in testing data?

2.	In Figures 2 and 3, the detailed results of the 'Mix' case should be reported to show the strength of implicit estimation of sequence depth and sequence error.

3.	In the section "the accuracy on predicting continental origins of white oak NGS samples using k-NN is markedly increased", the usage of the words "training" and "test sets" is somehow confusing. In the Conclusion section, the authors said that the neural-network regression model is pre-trained and applied to the white-oak dataset without changing parameters. Thus, using the words of training and test would make the reader confused since the neural-network model is not trained in the white-oak dataset.

4.	In the section "Running time and memory", the authors reported the running time of the methods using Cafe and CVTree. However, no accuracy results of these experiments are reported.

5.	In the References section, "dna" should be "DNA".

**Reviewer 2**

Please consider the following questions:

-Is the Software likely to be of broad utility? Is it easy to install and use?

**The software is easy to install and use. However, the tool needs to prove its general use by further testing on diverse and larger datasets.

-Does the software represent a significant advance over previously published tools (as demonstrated by direct comparison with available related software)?

**The software is an improvement over previously published software from the same lab. The comparison is limited as the new tool (Afann) is only compared with another tool (Cafe) from the same authors. The current analysis compares the average prediction accuracies before and after adjusting the bias. The comparison analysis with other state-of-the-art methods is missing.

-Please indicate briefly the novel features and/or advantages of the software and/or please reference the relevant publications and which alternatives, if any, it should be compared with.

**The dissimilarity analysis by taking bias into account is handled in a novel way. Performance comparison with other methods is missing. It is recommended to compare the proposed method with other alignment-free (e.g. Skmer, AAF, Phy-Mer) and alignment-based methods. The other methods need not have the same dissimilarity measures and can be compared based on the average prediction accuracy.

-Are the methods appropriate to the aims of the study, are they well described, and are necessary controls included?

**Yes, necessary controls are included, and methods are well described.

-Are the conclusions adequately supported by the data shown?

**The conclusions are supported by the data shown. However, we recommend this software to be tested on a more diverse and significantly larger dataset. For now, all datasets are composed of reads coming from similar sequences from closely-related species. It is suspected that this tool's behaviour will change if you include diverse sequences in a single dataset. This is important because most applications will *not* be to analyze sequences originating from the same test class. Thus, a mix of sequences from the different test classes (at different taxonomic levels) in a single dataset would be more realistic dataset to test.

-Are sufficient details provided to allow replication and comparison with related analyses that may have been performed?

** The results are reproducible with the proposed method.

-Is the paper of broad interest to others in the field, or of outstanding interest to a broad audience of biologists?

** The problem itself is of broad interest. A general-purpose alignment-free analysis tool that can adjust the bias would be very helpful. As recommended above, further testing is required to ensure the reliability of the proposed method.

Detailed comments:

- Line 70: The manuscript states that "The alignment-free dissimilarity between two NGS samples is determined by three factors which are alignment-free dissimilarity estimated based on their genomes and the bias caused by random sampling of each NGS sample.". This sentence needs clarification, as at first sight it would appear to mention only two factors. Later in the manuscript, "the bias caused by the random sampling of each NGS sample" is considered as two factors. ???

- The manuscript stated the use of 21 primates (lines 134,135) from reference 17 (Perelman et al., 2011, PLOS Genetics). The referred paper used 54 nuclear genes in 191 taxa including 186 primates representing 61 genera; there is no mention of a dataset of 21 genomes. This seems like a wrong reference.

- What is the biological or computational rationale behind the selection of these particular 21 primates?

- Sequence details such as length, accession numbers, etc. are missing.

- NCBI has more than 200 complete primate genomes, why not to test on a larger dataset?

- We recommend to mention 10-fold cross-validation in the paragraph discussing Table 1 results (for now, cross-validation text appears later in the method section, and one can misinterpret that the accuracies reported for each test case are for a single run).

- All three datasets (Primates, Mammals, White oak) have sequences from quite closely related species. It is not clear how this method would perform to other more diverse datasets. We recommend testing the method with a much larger set of sequences (in the thousands, not the tens), and include sequences that are very far from each other. It would also be interesting to test if the method can still tackle the bias when the sequence reads are associated with different test classes in the same dataset. A variety of sequences should be considered in the same dataset e.g. a dataset composed of sequences from Insects, Mammals, Birds, and others. Also, a larger dataset would reveal the method's scalability and respective time performance.

- The reported improvement in time efficiency is limited to the considered dissimilarity measures and an alignment-free method (Cafe) only. The best reported total time is ~22 minutes for a dataset of 92 white oak NGS samples of 300 Mbp.

There are much faster alignment-free methods [e.g., Kameris, ML-DSP, FFP] available, which are scalable although not tested yet on the NGS data. So, the claim "increases the speed of ds2 and d*2 calculation in existing tools by 100x" should clarify that the stated improvement in "Afann" is in comparison to the "Café" only.

The authors should compare their results with some of the state-of-the-art alignment-free software such as:

ML-DSP

ML-DSP: Machine learning with Digital Signal Processing for ultrafast, accurate and scalable DNA sequence classification at all taxonomic levels.G.Randhawa, K.Hill, L.Kari. BMC Genomics, 2019, 20:267.

KAMERIS

An open-source k-mer based machine learning tool for fast and accurate subtyping of HIV-I genomes S. Solis-Reyes, M.Avino, A. Poon, L.Kari. PLOS ONE 13(11):

FFP:

Sims GE, Jun SR, Wu GA, Kim SH. Alignment-free genome comparison with Feature Frequency Profiles (FFP) and optimal resolutions. In: Proceedings of the National Academy of Sciences of the USA. USA: National Academy of Sciences: 2009. p. 2677-82.

REGA

Pineda-Peña AC, Faria NR, Imbrechts S, Libin P, Abecasis AB, Deforche K, et al. Automated subtyping of HIV-1 genetic sequences for clinical and surveillance purposes: performance evaluation of the new REGA version 3 and seven other tools. Infection, Genetics and Evolution. 2013;19:337-348

SQEUAL

Pond SLK, Posada D, Stawiski E, Chappey C, Poon AF, Hughes G, et al. An evolutionary model-based algorithm for accurate phylogenetic breakpoint mapping and subtype prediction in HIV-1. PLoS Computational Biology. 2009;5(11):e1000581.

CASTOR

Remita MA, Halioui A, Diouara AAM, Daigle B, Kiani G, Diallo AB. A machine learning approach for viral genome classification. BMC Bioinformatics. 2017;18(208).

**Response to reviewers**

Reviewer #1: In this paper, the authors proposed a novel regression-based approach to adjusting the bias in comparing two genomes by using their corresponding NGS samples without assembly and alignment. They hypothesized that the dissimilarity of two genomes can be accurately predicted by a regression model of three factors: the dissimilarity calculated by using NGS samples and the features of two NGS samples.

To obtain the regression function, a neural-network model is proposed and the model

parameters are estimated by using a supervised-learning based approach. The

experiment performed on two independent datasets showed that the proposed

approach can improve the prediction performance. The authors also demonstrated the

generalization capability of the proposed approach by applying it to independent

datasets. Overall, this paper is well-written, well-structured and easy to follow. Before

the paper can be accepted for publication, there are a couple of issues needed to be

addressed.

*Response: We greatly appreciate the positive comments from the reviewer.*

Major issues

1.There exist a dozen alignment-free based methods. The authors need to compare the proposed approach with other alignment-free based approaches. It is not sufficient to only compare the results obtained before and after bias adjustment.

*Response: In this revision, we included the results of another state-of-the-art alignment-free method Mash and its variation Skmer which adjusts Mash distance based on NGS samples on all datasets.*

*Primate dataset:*

*Line 174-186, Figure S9*

*Line 238-258, Figure S13*

*Mammalian dataset:*

*Line 277-279, Figure S15*

*White Oak dataset:*

*Line 308-313, Table S2*

*Vertebrate dataset:*

*Line 362-364, Figure S17*

*The comparisons showed that our method outperforms Mash and Skmer on all datasets in terms of Spearman correlation coefficient between the alignment-free measure and the ground truth, and prediction accuracy for predicting species continent origin using K-nearest neighbor.*

2.It is not clear to me whether using the down-sampling procedure would improve the performance of the proposed approach when comparing two samples with different numbers of reads, that is, in the "Mix" case. For example, if we want to compare two samples with 15M and 9M reads, respectively. Figure 3(i) shows that the proposed approach without down-sampling has RMSE = 0.022 and SPC = 0.989. However, if we first down-sample the 15M sample to 9M and then compare the two samples, Figure 3(e) suggests that the proposed method achieved a better performance (RMSE = 0.011, SPC = 0.999). While the authors claimed that the down-sampling procedure would discard too many reads, leading to worse performance, I could only draw the opposite conclusion based on the result presented in Figure 3.

*Response: We thank the reviewer for carefully checking the figure. Down-sampling will certainly lead to the loss of information, but whether it will significantly lower the performance depends on whether the remaining reads are sufficient to represent the genomic signatures of its genome. Figure 3(e) shows that 9 M reads (0.45x) are sufficient to represent primate genomes with SPC 0.999, which proves that alignmentfree methods can be used on NGS samples of low sequencing depth. In this case, we wouldn’t say that down-sampling from 15 M to 9 M reads would discard too many reads since 9 M reads are sufficient. However, down-sampling from 15 M to 1 M reads would definitely be discarding too many reads since the SPC becomes 0.985. The reason why SPC in Figure 3(i) is lower than SPC in Figure 3(e) is that Figure3(e) shows an overall RMSE and SPC for all kinds of mix between 1M, 3M, …, 15M whereas Figure 3(e) shows only NGS samples of 9 M reads. As we can see from Figure 3(a) that 1 M samples have large RMSE and small SPC which lowers the performance in Figure 3(i). In practice, if there are NGS samples of 1M, 3M, …, 15 M reads and we want to compare them by down-sampling, we have to down-sample all of them to 1 M reads instead of 9 M reads, that will lead to Figure 3(a) with SPC 0.985 which is lower than Figure 3(i).*

Minor issues

1.Although the authors ensured that d(A, B) = d(B, A) by training on both samples with the same target, how close are they (d(A, B) and d(B, A)) in testing data?

*Response: In this revision, we calculated the SPC and RMSE between d(A, B) and d(B, A) and they are almost identical with SPC 0.999 and RMSE 0.005 and the results are presented as Figure S19 in supplementary material.*

*In our paper, we actually predicts the dissimilarity between A and B as the average of d(A, B) and d(B, A) so that the adjusted dissimilarity strictly satisfy the symmetry property. We failed to mention this implementation detail in the previous manuscriptand we added a description on this in Line 591-592 and the Line 629-632.*

2.In Figures 2 and 3, the detailed results of the 'Mix' case should be reported to show the strength of implicit estimation of sequence depth and sequence error.

*Response: The reason why we didn’t divide ‘Mix’ is that there will be 8 * 7 / 2 = 28 combinations and detailed results will make the figure crowded. For better visualization, we chose samples of 1 M, 5 M, 15 M reads and plotted all combinations between them in Figure S8 in supplementary material. A description is added in Line*

*171-173 and Line 219-220.*

3.In the section "the accuracy on predicting continental origins of white oak NGS samples using k-NN is markedly increased", the usage of the words "training" and "test sets" is somehow confusing. In the Conclusion section, the authors said that the neural-network regression model is pre-trained and applied to the white-oak dataset without changing parameters. Thus, using the words of training and test would make the reader confused since the neural-network model is not trained in the white-oak dataset.

*Response: We have changed “training” and “test sets” in the k-NN part to “reference” and “query sets” to avoid confusion.*

4.In the section "Running time and memory", the authors reported the running time of the methods using Cafe and CVTree. However, no accuracy results of these experiments are reported.

*Response: d2*, d2s and CVTree are 3 different dissimilarity measures. Café and Afann both implement all three methods. There is no difference between the results calculated by Café or Afann and we just want to state that Afann can calculate the same dissimilarity measures much faster than Café. The comparison between the performance of d2*, d2s and CVTree has been studied by several other previous studies and it is not a part of this study.*

5.In the References section, "dna" should be "DNA".

*Response: Corrected.*

Reviewer #2: ===

This box is for your report. All comments included here will be passed to the authors.

===

Please consider the following questions:

-Is the Software likely to be of broad utility? Is it easy to install and use?

**The software is easy to install and use. However, the tool needs to prove its general use by further testing on diverse and larger datasets.

*Response: We constructed a new diverse dataset composed of 67 vertebrate genomes from 5 different classes including fish, amphibians, reptiles, birds and mammals.*

*Line 347-361 and Figure 6 in the revised manuscript and Figure S16 in the supplementary material demonstrated that our method can greatly increase the performance after bias adjustment.*

-Does the software represent a significant advance over previously published tools (as

demonstrated by direct comparison with available related software)?

**The software is an improvement over previously published software from the same lab. The comparison is limited as the new tool (Afann) is only compared with another tool (Cafe) from the same authors. The current analysis compares the average prediction accuracies before and after adjusting the bias. The comparison analysis with other state-of-the-art methods is missing.

*Response: In this revision, we included the results of another state-of-the-art alignment-free method Mash and its variation Skmer which adjusts Mash distance based on NGS samples on all datasets.*

*Primate dataset:*

*Line 174-186, Figure S9*

*Line 238-258, Figure S13*

*Mammalian dataset:*

*Line 277-279, Figure S15*

*White Oak dataset:*

*Line 308-313, Table S2*

*Vertebrate dataset:*

*Line 362-364, Figure S17*

*The comparisons showed that our method outperforms Mash and Skmer on all datasets in terms of Spearman correlation coefficient between the alignment-free measure and the ground truth, and prediction accuracy for predicting species continent origin using K-nearest neighbor.*

-Please indicate briefly the novel features and/or advantages of the software and/or please reference the relevant publications and which alternatives, if any, it should be compared with.

**The dissimilarity analysis by taking bias into account is handled in a novel way. Performance comparison with other methods is missing. It is recommended to compare the proposed method with other alignment-free (e.g. Skmer, AAF, Phy-Mer) and alignment-based methods. The other methods need not have the same dissimilarity measures and can be compared based on the average prediction accuracy.

*Response: Please see our response to the above question.*

-Are the conclusions adequately supported by the data shown?

**The conclusions are supported by the data shown. However, we recommend this software to be tested on a more diverse and significantly larger dataset. For now, all datasets are composed of reads coming from similar sequences from closely-related species. It is suspected that this tool's behaviour will change if you include diverse sequences in a single dataset. This is important because most applications will *not* be to analyze sequences originating from the same test class. Thus, a mix of sequences from the different test classes (at different taxonomic levels) in a single dataset would be more realistic dataset to test.

*Response: We constructed a new diverse dataset composed of 67 vertebrate genomes from 5 different classes including fish, amphibians, reptiles, birds and mammals. (Line 528-537)*

*Line 347-361 and Figure 6 in the revised manuscript and Figure S16 in the supplementary material demonstrated that our method can greatly increase the performance after bias adjustment.*

-Is the paper of broad interest to others in the field, or of outstanding interest to a broad audience of biologists?

** The problem itself is of broad interest. A general-purpose alignment-free analysis tool that can adjust the bias would be very helpful. As recommended above, further testing is required to ensure the reliability of the proposed method.

*Response: Please see our response to the above question.*

Detailed comments:

- Line 70: The manuscript states that "The alignment-free dissimilarity between two NGS samples is determined by three factors which are alignment-free dissimilarity estimated based on their genomes and the bias caused by random sampling of each NGS sample.". This sentence needs clarification, as at first sight it would appear to mention only two factors. Later in the manuscript, "the bias caused by the random sampling of each NGS sample" is considered as two factors. ???

*Response: Thanks for the suggestion. It is now more clearly stated in Line 65-68 as “The alignment-free dissimilarity between two NGS samples A and B is determined by three factors which are alignment-free dissimilarity estimated based on their genomes, the bias caused by random sampling of NGS sample A and the bias caused by random*

*sampling of NGS sample B.”*

- The manuscript stated the use of 21 primates (lines 134,135) from reference 17 (Perelman et al., 2011, PLOS Genetics). The referred paper used 54 nuclear genes in 191 taxa including 186 primates representing 61 genera; there is no mention of a dataset of 21 genomes. This seems like a wrong reference.

- What is the biological or computational rationale behind

the selection of these particular 21 primates?

- Sequence details such as length, accession numbers, etc.

are missing.

- NCBI has more than 200 complete primate genomes, why not to

test on a larger dataset?

*Response: The reason why we used these 21 primates was that*

*in our previous study (Yang et al., 2017, Nucleic Acids Res) , we checked the 186 primates in (Perelman et al., 2011, PLOS Genetics) and found that only 21 of them have representative genomes on NCBI. Therefore, we used these 21 primates with known evolutionary distances to validate our methods. We added a detailed description about this in Line 499-505.*

*Information about species name, assembly accession number, length of 21 primate genome sequences is now in Table S4.*

*Information about species name, assembly accession number, length of 28 mammalian genome sequences is now in Table S5.*

*Information about run accession number, length and continental origin of 92 white oak NGS samples is now in Table S6.*

*Information about species name, class, assembly accession number, length of 67 vertebrate genome sequences is now in Table S7.*

- We recommend to mention 10-fold cross-validation in the paragraph discussing Table 1 results (for now, cross-validation text appears later in the method section, and one can misinterpret that the accuracies reported for each test case are for a single run).

*Response: Thanks for the suggestion. We have described our procedure more clearly in the legend of Table 1.*

- All three datasets (Primates, Mammals, White oak) have sequences from quite closely related species. It is not clear how this method would perform to other more diverse datasets. We recommend testing the method with a much larger set of sequences (in the thousands, not the tens), and include sequences that are very far from each other. It would also be interesting to test if the method can still tackle the bias when the sequence reads are associated with different test classes in the same dataset. A variety of sequences should be considered in the same dataset e.g. a dataset composed of sequences from Insects, Mammals, Birds, and others. Also, a larger dataset would reveal the method's scalability and respective time performance.

*Response: We constructed a new diverse dataset composed of 67 vertebrate genomes from 5 different classes including fish, amphibians, reptiles, birds and mammals. (Line 528-537*)

*Line 347-361 and Figure 6 in the revised manuscript and Figure S16 in the supplementary material demonstrated that our method can greatly increase the performance after bias adjustment.*

- The reported improvement in time efficiency is limited to the considered dissimilarity measures and an alignment-free method (Cafe) only. The best reported total time is ~22 minutes for a dataset of 92 white oak NGS samples of 300 Mbp. There are much faster alignment-free methods [e.g., Kameris, ML-DSP, FFP]

available, which are scalable although not tested yet on the NGS data. So, the claim "increases the speed of ds2 and d*2 calculation in existing tools by 100x" should clarify that the stated improvement in "Afann" is in comparison to the "Café" only.

The authors should compare their results with some of the state-of-the-art alignmentfree

software such as:

ML-DSP

ML-DSP: Machine learning with Digital Signal Processing for ultrafast, accurate and

scalable DNA sequence classification at all taxonomic levels.G.Randhawa, K.Hill,

L.Kari. BMC Genomics, 2019, 20:267.

KAMERIS

An open-source k-mer based machine learning tool for fast and accurate subtyping of

HIV-I genomes S. Solis-Reyes, M.Avino, A. Poon, L.Kari. PLOS ONE 13(11):

FFP:

Sims GE, Jun SR, Wu GA, Kim SH. Alignment-free genome comparison with Feature

Frequency Profiles (FFP) and optimal resolutions. In: Proceedings of the National

Academy of Sciences of the USA. USA: National Academy of Sciences: 2009. p. 2677-

82.

REGA

Pineda-Peña AC, Faria NR, Imbrechts S, Libin P, Abecasis AB, Deforche K, et al.

Automated subtyping of HIV-1 genetic sequences for clinical and surveillance

purposes: performance evaluation of the new REGA version 3 and seven other tools.

Infection, Genetics and Evolution. 2013;19:337-348

SQEUAL

Pond SLK, Posada D, Stawiski E, Chappey C, Poon AF, Hughes G, et al. An

evolutionary model-based algorithm for accurate phylogenetic breakpoint mapping and

subtype prediction in HIV-1. PLoS Computational Biology. 2009;5(11):e1000581.

CASTOR

Remita MA, Halioui A, Diouara AAM, Daigle B, Kiani G, Diallo AB. A machine learning

approach for viral genome classification. BMC Bioinformatics. 2017;18(208).

*Response: We have changed the statement so it now clearly states that the speed and memory improvement is in the comparison to Café.*

*We also compared the running time and memory usage to Mash, Skmer (Table 2, Table S2) and FFP (Table S2). It shows that Café is much slower than these methods whereas Afann is comparable to them.*

*For the other tools the reviewer listed, they are either too specific for viral genome classification or there’s no existing implementation that can be tested on Unix. The focus of our paper is to develop a method for correcting the bias for alignment-free sequence comparison based on NGS, so comparison with many of the other methods is beyond the scope of this paper. For large-scale comparison of alignment-free methods, we refer the readers to the most recent benchmark study of Zielezinski(https://genomebiology.biomedcentral.com/articles/10.1186/s13059-019-*

*1755-7).*

**Second round**

**Reviewer 1**

The authors have addressed all the issues I raised.

**Reviewer 2**

Afann: bias adjustment for alignment-free sequence comparison based on NGS using neural network regression

Though the authors addressed most of the issues, one major concern remains open. Both reviewers asked for a comparison with other alignment-free methods. Authors revised and added comparison with Mash and Skmer. The comparison is not fair because of the following reasons:

a. The authors tested Mash and Skmer at k=31 and Afann at k=12. A higher k-value doesn’t necessarily mean better results and all methods should be compared at the same value of k.

b. As k=31 is used for Mash and Skmer, that is the reason for them taking more time and memory. Naturally, using a lower k-value will take less time and memory, and vice-versa. Only a performance comparison at the same value of k for all the methods will be fair in showing which method outperforms the rest.

Suggested revisions: Please compare all the methods using the same k-value for comparison, to be fair and update the results accordingly.

**Response to reviewers**

**Reviewer 2**

We greatly appreciate your time and efforts in further reviewing the first revision of our manuscript and give valuable suggestions. The reviewers thought that it is unfair to compare with Mash and Skmer using K = 31 instead of the same shorter kmer length (K = 12, 14) as d2s and d2*. They mentioned that K = 31 does not necessarily have better results than K = 12 or 14 for Mash and Skmer, and K = 31 uses more time and memory usage. They wanted us to compare Mash and Skmer using the same kmer length as d2s and d2*. In our previous revision, we briefly mentioned that we used K = 31 to achieve the best performance of Mash and Skmer without detailed explanation.

We are sorry about the ambiguity and confusion, and we appreciate that reviewers

pointed it out.

Our response consists of three parts. In the first part, we explain why we used K = 31 for Mash and Skmer instead of K = 14 or K = 12 for both theoretical and empirical reasons. In the second part, we describe the changes in our latest revision. We tested the performance of Mash and Skmer using different kmer lengths (including the same kmer length as d2s and d2*) and different sketch sizes and showed that d2s and d2* adjusted by Afann have better performance than the best performance of Mash and Skmer on comparing NGS samples for all datasets. In the third part, we showed that Afann still has comparable speed as Mash and Skmer when the same kmer length is used although the performance of Mash and Skmer with K = 14 or 12 is far worse than d2s and d2*

1.The reason why we chose K = 31 for Mash and Skmer in our previous revision instead of using the same K = 14 or K = 12 as d2* and d2s:

Theoretically, the optimal kmer length for Mash and Skmer is longer than d2* and d2s. Because d2* and d2s are based on cosine distance where each kmer is represented by a numerical value but Mash and Skmer are based on Jaccard distance where each kmer is represented by either 0 or 1. As a consequence, each kmer is more informative in d2* and d2s than in Mash and Skmer. Therefore, K = 12 and K = 14 are appropriate for d2s and d2* but are too short for Mash and Skmer and will result in bad performance due to low sensitivity. The reason why we used the same kmer length for Mash and Skmer is that Skmer internally uses Mash and they have the same distance on comparing genomes. The only difference is that Skmer adjusts Mash distance on NGS samples. Empirically, kmer length smaller than 21 is not recommended for Mash and Skmer:

a.The following statement is from Skmer Github (https://github.com/shahabsarmashghi/

Skmer):

“Default k-mer size is set to 31 which is the maximum length allowed by Mash, and can be changed using -k option. We do not recommend using k-mers smaller than ~21, as k-mers without any shared evolutionary history start to seem similar just out of random chance. The sketch size can also be changed using -s option from its default value 10000000. Decreasing the sketch size will reduce the size of library on disk, but also compromises the accuracy of distance estimation.”

b.In the original publication of Skmer (Skmer: assembly-free and alignment-free sample identification using genome skims), the authors also used K = 31 and s = 10^7 for both Skmer and Mash on all different datasets. “For Skmer, we use the default parameters described above. For Mash, similar to Skmer, we used k=31 (selected empirically; Additional file 1: Figure S14) and sketch size 10^7”

c.When we ran Mash using K = 14 on the primate dataset, we received the following warning message.

“WARNING: For the k-mer size used (14), the random match probability (0.927885) is above the specified warning threshold (0.01) for the sequence "/home/cmbpanasas2/ kujin/PrimateGenomes/tarsiussyrichta.fna" of size 3453864774 (and 20 others). Distances to these sequences may be underestimated as a result. To meet the threshold of 0.01, a k-mer size of at least 20 is required. See: -k, -w.”

Therefore, we thought the comparison would not be fair for Mash and Skmer if we used K = 12 or K = 14 because they are too short and it would lead to bad performance of Mash and Skmer. Instead, we wanted to compare our method with the best performance of Mash and Skmer. So we chose K = 31 based on both the theoretical and empirical reasons mentioned above.

2.Revision:

In order to compare with the best performance of Mash and Skmer on different datasets. We tried different kmer lengths and sketch sizes for Mash and Skmer on each dataset.

For primate dataset (Line 174 – Line 192, Line 244 – Line 261):

We first compared Mash distances based on primate genomes with their evolutionary distances using K = 14 (same as d2* and d2s), K = 21 (default of Mash), K = 31 (maximum kmer length allowed by Mash) and sketch size s = 10^3 (default of Mash), s= 10^5, s = 10^7 (default of Skmer). Results are shown in Figure S9 and Mash using K = 21 and s = 10^7 has the highest spearman correlation with the evolutionary distances. (We only tested Mash because Skmer is exactly the same as Mash on comparing genomes)

Therefore, we chose K = 21 and s = 10^7 and tested Mash and Skmer on primate NGS samples and results are shown in Figure S10 and Figure S14. Our results showed that adjusted d2s (0.989) and d2* (0.994) have much higher Spearman correlation coefficients than Mash (0.860) and Skmer (0.766).

For mammal dataset (Line 280 – Line 290):

Similarly, we first compared Mash distances based on mammalian genomes with their evolutionary distances using K = 14, K = 21, K = 31 and sketch size s = 10^3, s = 10^5, s = 10^7. Results are shown in Figure S16 and Mash using K = 31 and s = 10^7 has the highest spearman correlation.

Therefore, we chose K = 31 and s = 10^7 and tested Mash and Skmer on mammalian NGS samples and results are shown in Figure S17. Our results showed that adjusted d2s (0.967) has much higher Spearman correlation coefficients than Mash (0.789) and Skmer (0.688).

For oak dataset (Line 318 – Line 324) We tested Mash and Skmer on oak NGS dataset using K = 12 (same as d2* and d2s), K = 21, K = 31 and sketch size s = 10^3, s = 10^5, s = 10^7. Results are shown in Table S2, Table S3. Both Mash and Skmer have highest average accuracy with K = 31 and s = 10^7 for 1-NN and 2-NN. The prediction accuracies for 1-NN and 2-NN are the same and are higher than that for k-NN with k > 2. Our results showed that adjusted d2* (0.96) has much higher average prediction accuracy than Mash (0.64) and Skmer (0.73) for 1-NN and 2-NN.

For vertebrate dataset (Line 374 – Line 376):

The true evolutionary distances are unknown for this dataset, so we chose K = 31 and s = 10^7 for Mash and Skmer based on the theoretical and empirical reasons we mentioned before. Results are shown in Figure S19. Our results showed that adjusted d2s (0.935) and d2* (0.959) have much higher Spearman correlation coefficients than Mash (0.747) and Skmer (0.735).

For all datasets, d2* and d2s have better performance than Mash and Skmer in terms of Spearman correlation coefficients and prediction accuracy. In addition, Mash and Skmer using K = 31 have the best performance in mammal and oak datasets among different kmer lengths which proves that our choice of K = 31 in the previous revision was reasonable.

3.Running time and memory usage (Line 403 – Line 420):

We compared the running time and memory usage of Afann with Mash and Skmer. Results are shown in Table 2, Table S4. Even though K = 12 and K = 14 are too short for Mash and Skmer and they did show worse performance than longer K in this study, we still included their running time to show the speed of Mash and Skmer using the same kmer length as d2s and d2*. Our results showed that Afann still has comparable speed as Mash and Skmer when the same K is used.

We did not claim Afann outperforms Mash and Skmer in terms of speed. We showed that Afann is much faster than our previous software Café and it can now run in a comparable amount of time as Mash and Skmer. The most important advantage of Afann is that it has better performance on comparing NGS samples over existing tools. In addition, users do not need to worry about its speed because it is as fast as other fast alignment-free tools such as Mash and Skmer

**3rd round**

**Reviewer 2**

I have looked at the revisions and the authors addressed all the issues in detail. I am satisfied with their reasoning and new experiments. I don't have further comments and recommend the manuscript for acceptance.
